# Rapid autopsies to enhance metastatic research: the UPTIDER post-mortem tissue donation program

**DOI:** 10.1038/s41523-024-00637-3

**Published:** 2024-04-24

**Authors:** Tatjana Geukens, Maxim De Schepper, Wouter Van Den Bogaert, Karen Van Baelen, Marion Maetens, Anirudh Pabba, Amena Mahdami, Sophia Leduc, Edoardo Isnaldi, Ha-Linh Nguyen, Imane Bachir, Maysam Hajipirloo, Gitte Zels, Josephine Van Cauwenberge, Kristien Borremans, Vincent Vandecaveye, Birgit Weynand, Peter Vermeulen, Eleonora Leucci, Maria Francesca Baietti, George Sflomos, Laura Battista, Cathrin Brisken, Patrick W. B. Derksen, Thijs Koorman, Daan Visser, Colinda L. G. J. Scheele, Daniela S. Thommen, Sigrid Hatse, Sarah-Maria Fendt, Evy Vanderheyden, Thomas Van Brussel, Rogier Schepers, Bram Boeckx, Diether Lambrechts, Giuseppe Marano, Elia Biganzoli, Ann Smeets, Ines Nevelsteen, Kevin Punie, Patrick Neven, Hans Wildiers, François Richard, Giuseppe Floris, Christine Desmedt

**Affiliations:** 1 Laboratory for Translational Breast Cancer Research, Department of Oncology, KU Leuven Leuven, Belgium; 2 Department of General Medical Oncology, University Hospitals Leuven, Leuven, Belgium; 3 Department of Pathology, University Hospitals Leuven, Leuven, Belgium; 4 Department of Forensic Medicine, University Hospitals Leuven, Leuven, Belgium; 5 Department of Gynecology and Obstetrics, University Hospitals Leuven, Leuven, Belgium; 6 Department of Anesthesiology, Institut Jules Bordet, Brussels, Belgium; 7 Department of Radiology, University Hospitals Leuven, Leuven, Belgium; 8 Centre for Oncological Research (CORE), University of Antwerp, Antwerp, Belgium; 9 TRACE and Laboratory for RNA Cancer Biology, Department of Oncology, KU Leuven Leuven, Belgium; 10 ISREC - Swiss Institute for Experimental Cancer Research, School of Life Sciences, Ecole Polytechnique Fédérale de Lausanne (EPFL), Lausanne, Switzerland; 11 The Breast Cancer Now Toby Robins Breast Cancer Research Centre, The Institute of Cancer Research, London, UK; 12 Department of Pathology, University Medical Center, Utrecht, The Netherlands; 13 Laboratory of Intravital Microscopy and Dynamics of Tumor Progression, Department of Oncology, VIB-KU Leuven Center for Cancer Biology, Leuven, Belgium; 14 Division of Molecular Oncology and Immunology, Netherlands Cancer Institute, Amsterdam, The Netherlands; 15 Laboratory of Experimental Oncology, Department of Oncology, KU Leuven Leuven, Belgium; 16 Laboratory of Cellular Metabolism and Metabolic Regulation, VIB-KU Leuven Center for Cancer Biology, VIB, Leuven, Belgium; 17 Laboratory of Cellular Metabolism and Metabolic Regulation, Department of Oncology, KU Leuven and Leuven Cancer Institute (LKI), Leuven, Belgium; 18 Laboratory for Translational Genetics, Department of Human Genetics, KU Leuven, Leuven, Belgium, and VIB Center for Cancer Biology, Leuven, Belgium; 19 Unit of Medical Statistics, Biometry and Epidemiology, Department of Biomedical and Clinical Sciences (DIBIC) “L. Sacco” & DSRC, LITA Vialba campus, Università degli Studi di Milano, Milan, Italy; 20 Department of Surgical Oncology, University Hospitals Leuven, Leuven, Belgium

**Keywords:** Breast cancer, Tumour heterogeneity, Cancer microenvironment, Cancer models, Metastasis

## Abstract

Research on metastatic cancer has been hampered by limited sample availability. Here we present the breast cancer post-mortem tissue donation program UPTIDER and show how it enabled sampling of a median of 31 (range: 5-90) metastases and 5-8 liquids per patient from its first 20 patients. In a dedicated experiment, we show the mild impact of increasing time after death on RNA quality, transcriptional profiles and immunohistochemical staining in tumor tissue samples. We show that this impact can be counteracted by organ cooling. We successfully generated ex vivo models from tissue and liquid biopsies from distinct histological subtypes of breast cancer. We anticipate these and future findings of UPTIDER to elucidate mechanisms of disease progression and treatment resistance and to provide tools for the exploration of precision medicine strategies in the metastatic setting.

## Introduction

While our understanding of breast cancer biology is extensive in the early setting, it is limited in metastatic disease. It is essential however to evaluate tissues in the metastatic setting too, as the biology and the mechanisms driving disease progression and treatment resistance may diverge from those in early stages^1,2^. At the phenotypic level, for example, discrepancies between the estrogen receptor (ER), progesterone receptor (PR) and human epidermal growth factor receptor 2 (HER2) status of the primary tumor and its respective metastases are frequently observed^3^. At the genomic level, metastases show not only a higher number of genomic alterations, but also enrichment for different driver events, often associated with treatment resistance^1,4–10^. On the organ level, different metastatic lesions are surrounded by distinct metabolic and immune micro-environments^2,8,11–13^. This lack of common features impairs systemic treatment successes, and invariably therapeutic resistance will develop. It also complicates predictive biomarker profiling at one point in time, as a single biopsy might not reflect the susceptibility to treatment of the different tumor subclones co-existing at any given moment^14^.

The limited sample availability from patients with metastatic cancer is a key hurdle in assessing this intra-patient tumor heterogeneity. Some (inter)national and institutional initiatives have been set up to specifically collect and molecularly characterize metastatic samples, thereby not only yielding important repertoires for science but also providing evidence for the benefits of genomic-based treatment decisions^9,10,15–18^. Although these efforts are crucial, they often investigate only a small number of samples per patient and thus still provide incomplete information to answer biological questions in the metastatic setting.

Enhancing access to samples in late-stage cancer is crucial to advance research and ultimately patient care. One way of doing this, is through research autopsies, a procedure in which samples are collected promptly after death for the purpose of translational research^19–21^. Advantages of this approach are the possibility of sampling to completion, multiregional sampling (to reflect spatial heterogeneity within each lesion as well as between lesions), and high-volume sampling to allow different downstream analyses to be performed. Important knowledge already generated through such autopsies was recently reviewed elsewhere and included insights into clonal disease progression, mechanisms of metastatic spread including cross-seeding, and genomic mechanisms behind underlying treatment resistance^19,22–24^. Additionally, autopsies have the potential to enable the creation of much needed in vivo and in vitro tumor models^19^. Setting up and running such programs remains however logistically challenging, and only few projects have provided comprehensive information on their procedures and outcomes^25–30^. Sharing experiences is of utmost importance, as it can facilitate the set-up of other platforms in- and outside the oncology field and enthuse researchers for future collaborations. Here, we therefore present our breast cancer tissue donation program UPTIDER (UZ/KU Leuven Program for Post-mortem Tissue Donation to Enhance Research, NCT04531696), which so far performed autopsies on 20 patients with metastatic breast cancer, yielding over 3,000 tumor tissue samples. We also present the first results of scientific interest, focusing on sample quality regarding RNA and protein expression with increasing time after death and the development of tumor models from currently underrepresented subtypes of breast cancer.

## Results

### Set-up and conduct of a collaborative breast cancer tissue donation program

**Fig. 1:**
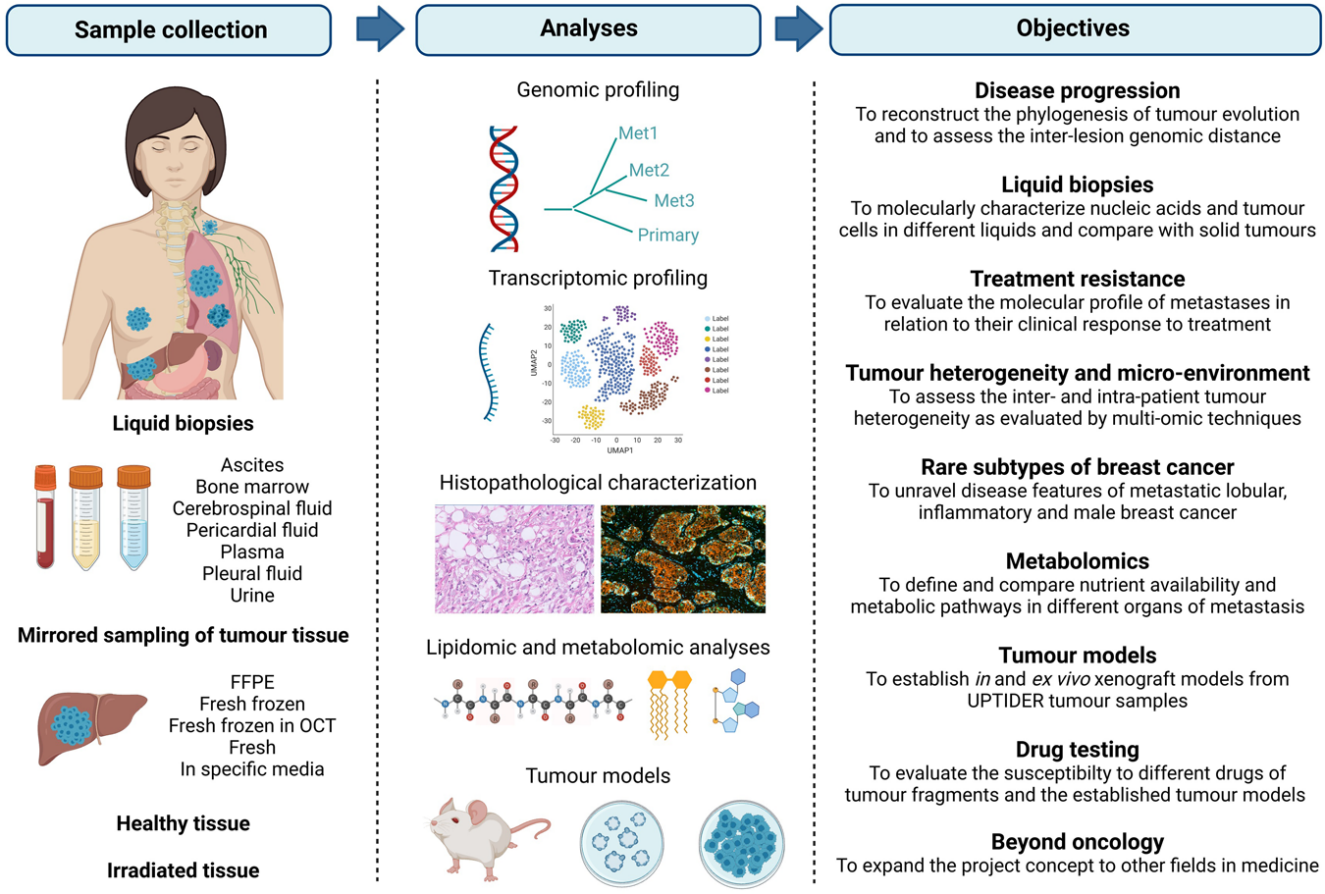
Research strategy for the UPTIDER program. As defined through discussions with academic and clinical collaborators. Objectives as presented on the right are expected to evolve over time as clinical and scientific insights progress. Created with BioRender.com.

A core multidisciplinary research team led the set-up of our rapid autopsy program UPTIDER. First steps in the process were defining the scientific questions of interest and possible strategies to answer those questions, thus moving away from a pure “biobank” approach (Fig. 1). Early discussions with collaborating academic partners were crucial in these steps, to assure maximal leverage of the unique sample repositories. Sample processing strategies were then tailored to the specific downstream techniques planned to be performed on each specimen type (Fig. 1) and are further detailed in the Methods section. From a clinical point of view, three departments were involved in the set-up. The department of forensic medicine (including the morgue) provided the expertise for specific technical aspects of the tissue donation procedure. The department of pathology provided expertise in clinical autopsy, sample processing capacity, and histopathological analyses. Moreover, the clinical oncological and palliative care teams assured realistic patient inclusion strategies.

Key logistical procedures that were established next, were as follows. An electronic case report form (eCRF) was designed in REDCap® for capturing relevant patient and tumor characteristics. Dynamic structured query language (SQL) was implemented to allow the registration of individual lesion-level treatment responses given the importance of heterogeneity described above. A LabCollector®-based platform was set up to register and manage patient samples. Samples planned to be collected during the autopsy based on latest imaging and on specific research interests, are first listed on a coded sheet (“*tissue donation plan”*). The predetermined donation plan includes the expected sample category (pathological versus non-malignant versus irradiated tissue), sample location encoded using the ICD-O-3 codes or liquid type, and sample processing strategy^31^. This donation plan is imported into our lab management system, resulting in the preregistration of hundreds of samples at once, while adding additional (“ad hoc”) samples is still possible during the autopsy. Each sample receives a unique barcode (QR code) and a patient-linked ID allowing robust tracking. Lastly, practical logistical aspects were tackled. Funding was secured first through a research grant from the affiliated hospital, citing cruciality and reported feasibility, and later through a university grant. A company for 24 h/7d transport of participating patients passing away at home or in hospice was contracted. Required sample processing infrastructure (centrifuges, −80 °C freezer, sample registration and handling stations) was purchased and installed in the morgue.

**Fig. 2:**
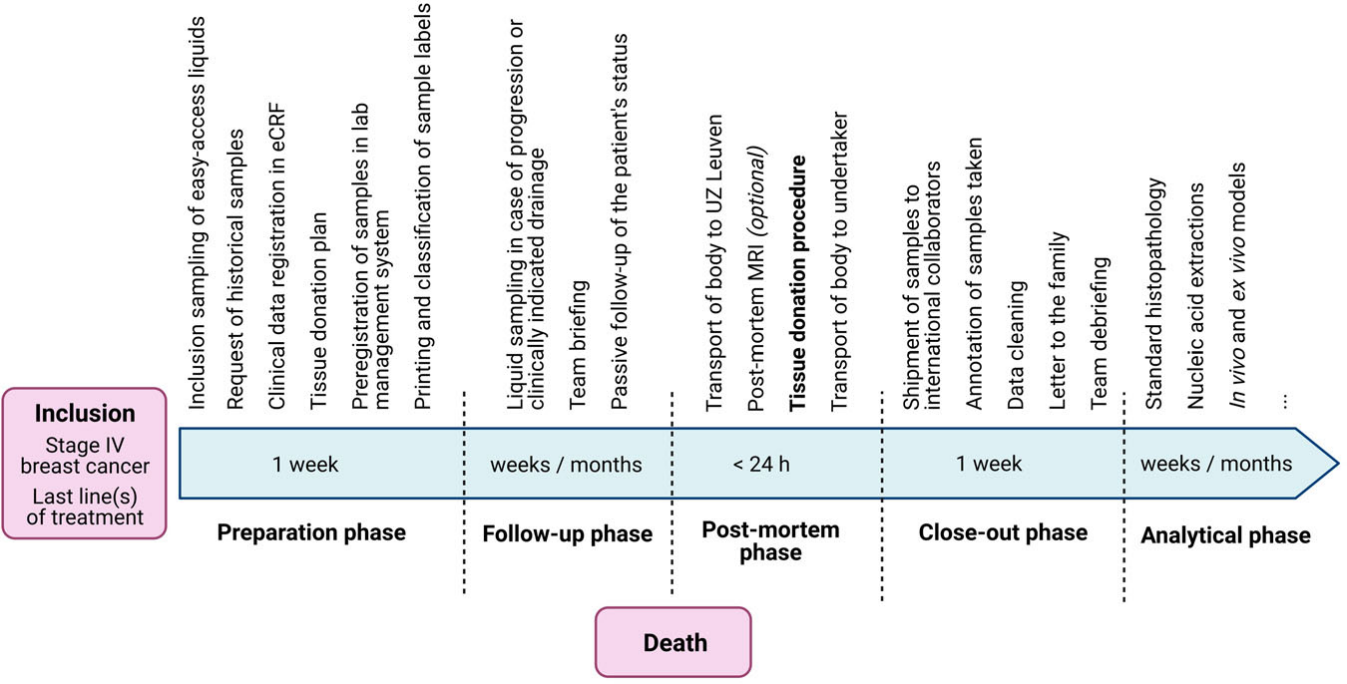
Study design and workflow of the UPTIDER program. FFPE formalin-fixed paraffin-embedded, OCT Optimal Cutting Temperature compound, eCRF electronic Case Report Form, MRI Magnetic Resonance Imaging. Created with BioRender.com.

The workflow of the project itself, from patient consenting down until the actual moment of tissue donation, was set up as follows (Fig. 2). Patients with stage IV breast cancer (either de novo or relapsed) followed in our institution University Hospitals Leuven were informed by their treating physician about UPTIDER during later line(s) of treatment or occasionally when metastatic patients themselves expressed the desire to contribute to science post-mortem. UPTIDER additionally allowed the inclusion of patients residing in Belgium or in neighboring countries but close to the Belgian border, who are referred to our center specifically for the study. Upon signature of the informed consent form (ICF), inclusion sampling of easy-access liquid biopsies (blood, urine, saliva) was performed. Any available pre-mortem tissue samples and/or extracted nucleic acids were requested from clinical or study archives. These samples will, whenever possible, undergo the same downstream analyses as the samples collected at autopsy to allow longitudinal evaluation of disease features. The tissue donation plan was prepared in the patient-specific manner as described above. The patient then remained under passive follow-up in the study, with possible repetition of the liquid sampling in case of progression and/or treatment switch. Additional samples, such as pleural fluid or ascites, were collected only in case of a clinically indicated drainage. At the moment of passing away, the UPTIDER team was notified (24 h/7d) and immediate transport of the patient’s body to University Hospitals Leuven was organized. If feasible, a whole-body MRI was performed immediately before start of the tissue donation to further guide the autopsy and for later translational research. The actual tissue donation procedure is described in detail in the Methods section. In brief, all body fluid types were collected first and stored as supernatant aliquots and cell pellets (Fig. 1). Next, organs were examined in a patient-specific order, and samples from all identified metastases and selected non-tumor tissues were stored in multiple conditions, depending on the analyses planned to be performed on them.

### Successful inclusion of patients with diverse breast cancer subtypes

From ethical committee (EC) approval on November 30th 2020 up until data cut-off on January 15th 2023, 28 patients had consented in writing to participate in the study. Two of these included patients withdrew consent due to psychosocial reasons, and their data is not presented in this manuscript. Other patients were approached carefully, out of those thirteen received an ICF but decided not to participate, with the most common reason for refusal being patient or family objection against the autopsy procedure. We saw a declining rate of these refusals over time, as physicians gained more experience with informing the patients about the project (using the wording ‘tissue donation program’ instead of ‘autopsy procedure’ and selecting more carefully the patients they approach) and as discussions with the family became easier after lifting of COVID-19 restrictions. The 26 remaining patients included individuals with less common clinical and histological subtypes of breast cancer: 6 patients with invasive lobular carcinoma (ILC), 5 patients with both ILC and invasive carcinoma of no special type (NST) (either mixed (*n* = 4), or in two distinct primary tumors (*n* = 1)), 1 patient with metaplastic carcinoma and 3 patients with inflammatory breast cancer (Supplementary Table 1). Treatment for patients with breast cancer has evolved in the last years and recently approved targeted treatments received by the patients included cyclin-dependent kinase 4/6 (CDK4/6) inhibitors, immune checkpoint inhibitors, poly ADP ribose polymerase (PARP) inhibitors, phosphoinositide 3-kinase (PI3K) inhibitors, antibody-drug conjugates and investigational drugs. In terms of pre-mortem samples, archived primary breast tumor samples were retrieved from the pathology department for all patients, archived metastatic samples for 15 patients, and plasma or extracted cell-free DNA from the time of first diagnosis and/or at subsequent timepoints for 12 patients (from a separate biobanking program in our institution). Thus, the inclusion of a substantial number of patients with breast cancer in a relatively short period of time, with unique features and historical pre-mortem samples available, was feasible.

### Extensive liquid and tissue sampling during the rapid autopsies

**Fig. 3:**
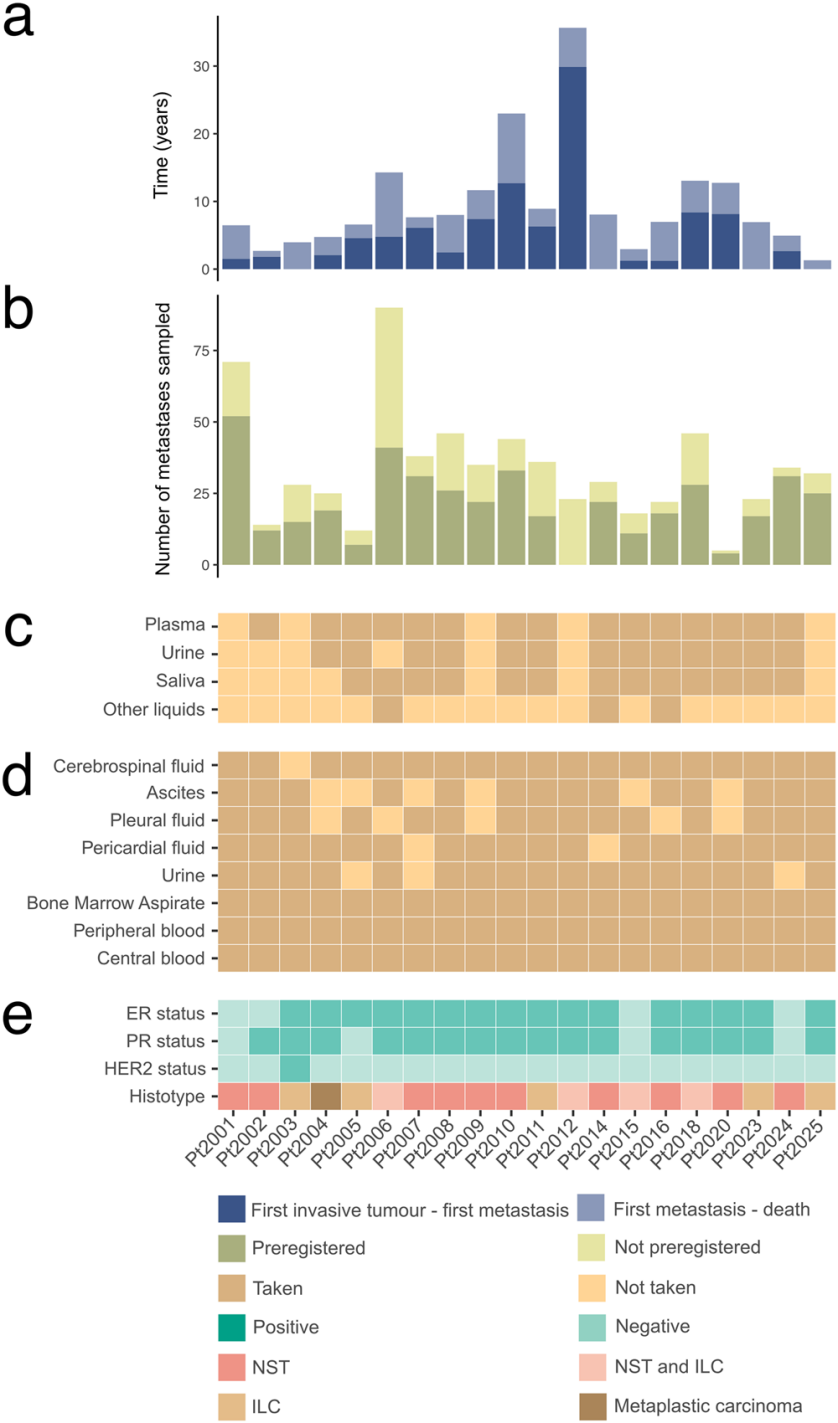
Patient and autopsy details. **a** Time (in years) between first invasive diagnosis and first metastasis (dark blue) and between first metastasis and death (light blue). **b** Total number of metastases sampled per patient at autopsy, either planned to be sampled and preregistered in LabCollector® before the autopsy (dark green) or found only during the autopsy (non pre-registered, light green). For Pt2012 the time between enrollment and death was too short to preregister any samples. **c** Liquid biopsies were sampled prospectively for UPTIDER premortem, either at inclusion or during follow-up. The collection of urine and saliva was only implemented after Pt2003. **d** Liquid biopsies collected during the autopsy. **e** Histopathological characteristics of the primary tumor. Of note, Pt2001 is considered ER-negative at primary diagnosis as per diagnostic biopsy but had one sample from the surgical resection exhibiting ER expression. All metastatic samples pre- and post-mortem were ER-negative. ER = Estrogen Receptor, PR = Progesterone Receptor, NST = Invasive Breast Carcinoma of No Special Type, ILC = Invasive Lobular Carcinoma.

At the cut-off date, 20 autopsies were performed (Fig. 3), representing a median of 1 autopsy per month. Median age at breast cancer diagnosis was 50 years (range: 36–80) for these patients and 60 years at time of death (range: 39–88) (Supplementary Table 2). Progression times for each patient are depicted in Fig. 3a (between the first invasive diagnosis and first metastasis (dark blue) and first metastasis and death (light blue)). Median time between inclusion in UPTIDER and death was 64.5 days (range: 6–177). Nine patients died in the hospital, the others died at home or in a hospice 10–177 (range) km away. A whole-body MRI was performed before the start of the autopsy in 8 patients, for the others, MRI would have delayed the autopsy too much or was unavailable at the time of death. The autopsy procedure itself started at a median of 3.0 h after death (range: 1.8–5.9) and lasted for 6.5 h (3.6–9.3)(Supplementary Fig. 1). Seventy-nine percent of autopsy hours did not fall within normal working hours. Each autopsy involved a team of 11 members (median; range: 6–15) present.

Solid samples were collected from a median of 31 metastases (range 5–90) (Fig. 3b) and 9.5 non-tumor tissues (range: 5–22) per patient. For 91% of all metastases sampled, we were able to collect at least mirrored fresh frozen (FF), fresh frozen in Optimal Cutting Temperature compound (FF-OCT) and formalin-fixed paraffin-embedded (FFPE) samples. The remaining lesions were either too small to divide or collected as supposedly non-tumor tissues. Additionally, fresh tissue samples for immediate metabolomic analysis or tumor model generation, and samples frozen in carboxymethylcellulose (CMC) for lipidomic analysis were taken whenever possible. For these 20 patients, this strategy resulted in over 3000 samples being stored in different conditions from a total of 671 metastases. In patients where samples were registered upfront via the tissue donation plan (*n* = 19, all except for Pt2012), 33.5% (217/648) of all metastases sampled was still encoded ad hoc during the autopsy (Fig. 3b, dark green). The organs classically known to be involved in metastatic BC such as liver, bones, distant lymph nodes and pleura were the most frequent metastatic locations being observed in more than 15 patients. Metastases in the central nervous system (CNS: brain, meninges or spinal cord) were present in 9 patients, with 7 presenting brain metastases, 4 metastases in the meninges and 1 in the spinal cord.

Liquid sample types that could be retrieved pre- and post-mortem are shown in Fig. 3c and 3d, respectively (dark orange). Forty-five percent of patients (*n* = 9) have had all post-mortem liquid types stored for further analysis, for the others no ascites and/or pleural fluid was present or urine or cerebrospinal fluid could not be retrieved (range: 5–8 liquid types/patient). These numbers clearly highlight that post-mortem tissue donation programs have the potential to generate unique and extensive biorepositories of liquid and solid samples from patients with metastatic disease.

### Appropriate operating procedures can reverse subtle transcriptional and proteinic impact of increasing post-mortem interval

**Fig. 4:**
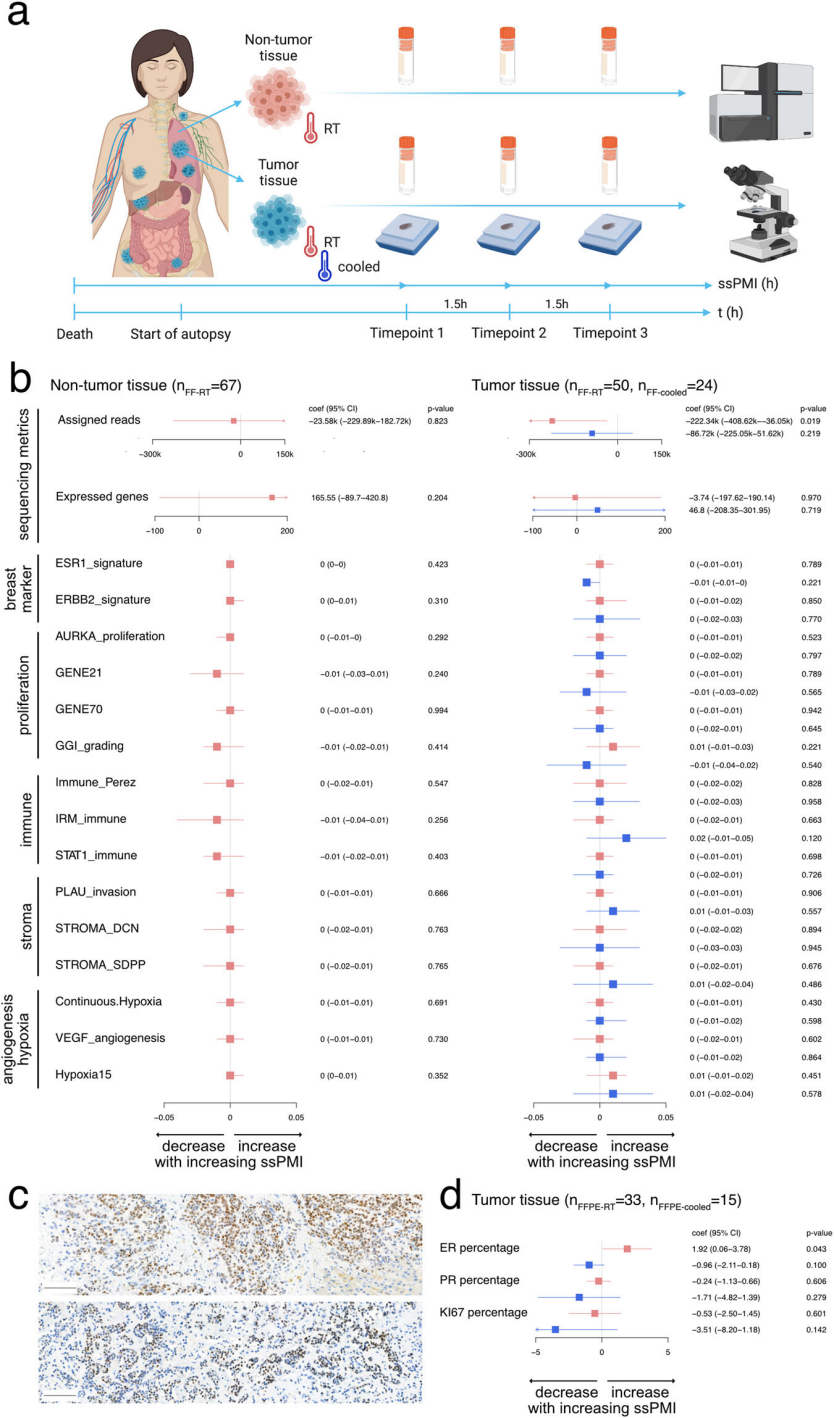
Transcriptomic and protein expression profiles in function of post-mortem interval in non-tumor and tumor tissues. **a** Design of the experiment. Fresh frozen samples (illustrated as tubes) and/or Formalin-Fixed Paraffin-Embedded (FFPE) samples (illustrated as blocks) from non-tumor or tumor tissues were taken repeatedly from the same tissue area (at 1.5 h time intervals) during the autopsy. Sample specific post-mortem interval (ssPMI) was defined as the time between death of the patient and fixation of the sample. Created with BioRender.com. **b** Results of the testing strategy evaluating the association between quality metrics and ssPMI at the RNA level regarding the non-tumor (left) and the tumor tissues (right) for samples kept at room temperature (RT, in red) and cooled between 4 °C and 10 °C (in blue). Linear coefficient (coef), 95% confidence interval (95% CI) and *p*-values are reported in the forest plots. Potential non-linear associations are reported in supplementary appendix. For the assigned reads, numbers are reported in kilo (1k = 1000 reads). **c** IHC slides of a liver metastasis of Pt2023 stained for ER, at time point 1 (top) and after cooling at time point 3 (below). Scale bar = 100 μm. **d** Results of the testing strategy evaluating the association between ER, PR and KI67 markers and ssPMI at the protein level for samples kept at room temperature (in red) and cooled between 4 °C and 10 °C (in blue). Linear coefficient (coef), 95% confidence interval (95% CI) and *p*-values are reported in the forest plots. No indication for non-linearity was found.

As RNA is known to be fragile and as our tissue donation procedures take several hours to complete, we set up an experiment to assess sample quality at the transcriptomic level over time^32,33^. We repeatedly sampled in a first instance non-tumor and tumor tissues at 1.5 h time intervals leaving the tissues at room temperature (*n* = 117) (Fig. 4a). Sample-specific post-mortem interval (ssPMI) was defined as the time between death of the patient and fixation of the sample (in hours (h)). Median ssPMI was 7.0 h (interquartile range (IQR): 3.2 h, range: 3.1 h–11.1 h). Following sequencing, several quality metrics were investigated: (i) sequencing metrics: number of reads assigned to genes (assigned reads), and number of expressed genes, (ii) transcriptomic metrics: a selection of 15 gene signatures covering different aspects of the tumor and its micro-environment such as breast markers, proliferation, immunity, angiogenesis, hypoxia, and stroma (Fig. 4b, Supplementary Table 3, Supplementary Figs. 2 and 3). At room temperature, in non-tumor and tumor tissues, the medians of assigned reads were 3,145,874 (IQR: 4,572,968) and 2,776,523 (IQR: 1,690,333), respectively. In non-tumor tissues, we did not observe any association between the ssPMI and the sequencing metrics or gene expression profiles (Fig. 4b, left in red). However, in tumor tissue, we observed a linear decrease in the number of assigned reads with increasing ssPMI (coefficient −222,336.39, 95% confidence interval (CI): −408619.29 to −36053.49, *p* = 0.019) but this was not reflected in the number of expressed genes or in the gene expression signatures (Fig. 4b, right in red). To investigate whether cooling the organs as soon as removed from the body could avoid this decrease in assigned reads, we also conducted an experiment where tumor samples (*n* = 24) were kept in iced water (between 4 °C and 10 °C). In this setting, we did not observe the decrease in assigned reads and both sequencing metrics and gene expression profiles remained stable (Fig. 4b, right in blue). Of note, in tumor tissues, two immune and two stromal signatures revealed a trend for a non-linear evolution with increasing ssPMI (Supplementary Figs. 4 and 5), but the changes were too subtle to be considered further.

In a similar manner, and because immunohistochemistry results have been reported to vary with cold ischemia time^34,35^, we investigated the evolution of the protein expression of key markers in breast cancer including ER, PR, and KI67, leveraging a cohort of 33 samples kept at room temperature and 15 cooled samples. At room temperature, we observed a small increase over time in the percentage of tumor cell expressing ER (1.92% per hour, 95% CI: 0.06–3.78%, *p* = 0.043) while the other markers remained stable (Fig. 4d, in red). This evolution was no longer observed when the samples were cooled (Fig. 4d in blue and Supplementary Fig. 6).

In conclusion, our easy-to-implement approach of organ cooling proved to be effective to preserve the RNA and protein expression of the samples for an extended period, specifically up to 11 h, our maximal ssPMI. We have now adopted this procedure as a standard practice for all subsequent autopsies.

### Establishment of various types of breast cancer tumor models from post-mortem samples

**Fig. 5:**
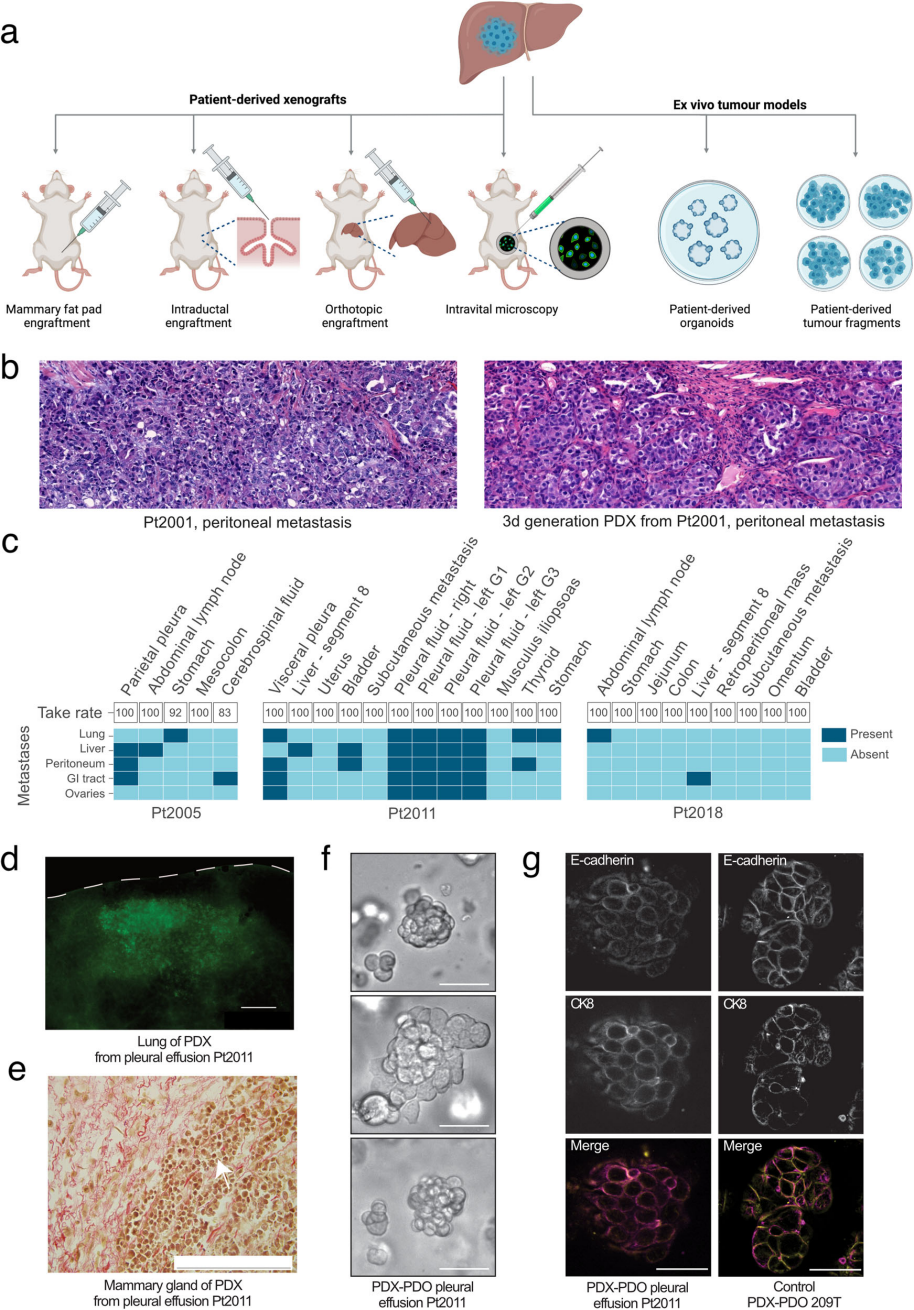
Establishment of different types of preclinical murine tumor models from post-mortem samples. **a** Different strategies for tumor model development within UPTIDER. **b** Hematoxylin-eosin (H&E) staining of a peritoneal lesion of Pt2001 (left) and the corresponding H&E of a third-generation mouse model of the same lesion (right). **c** Summary graph showing the 24 rapid autopsy samples from three patients with invasive lobular carcinoma, the intraductal take rates and the metastases detected at the endpoint. G1, 2, 3: generation 1, 2, 3; GI tract Gastrointestinal Tract. **d** Fluorescence stereo-micrograph of a lung lobe from an immunodeficient (NGS) mouse two months after intraductal injection with cells derived from pleural effusion of Pt2011. Scale bar = 1000 μm. **e** Representative micrograph of picrosirius red-stained histological section of a tumor formed in the mammary gland two months after intraductal injection of cells derived from a pleural effusion of Pt2011. Arrow points to single cell files. Scale bar = 200 μm. **f** Representative differential interference contrast microscopy (DIC) images of the PDX-PDO organoid line derived from a pleural effusion of Pt2011 cultured in a 3D-basement extract-derived matrix. Note the non-coherent “grape-like” phenotypic structures of the PDO model. Scale bar = 50 µm. **g** Immunofluorescence of the PDX-PDO organoid line derived from a pleural effusion of Pt2011 (left) and a control breast carcinoma of the no specific type E-cadherin positive PDO-PDX model 209 T (right). Expression and localization of E-cadherin is shown in the top panels and cytokeratin 8 (CK8) in the middle panels. Merged images are shown in the bottom panels. Scale bar = 5 µm.

Given the unique access to metastatic samples generated by the UPTIDER program, collaboration with multiple academic partners was set up to generate in vivo and ex vivo tumor models. Different approaches were employed including patient-derived tumor xenograft (PDX) models, combination of mouse models with state-of-the-art imaging techniques such as high-resolution intravital microscopy, organoid development, and patient-derived tumor fragment (PDTF) platforms (Fig. 5a)^36–39^.

The TRACE PDX platform at KU Leuven, one of the founding members of the international EurOPDX consortium (www.europdx.eu), focused on the establishment and characterization of preclinical tumor models from all UPTIDER breast cancer subtypes for subsequent therapeutic and biological investigations. Three UPTIDER models have been successfully established via mammary gland implantation, including one TNBC and two from a metaplastic carcinoma with predominant squamous differentiation, a rare breast cancer histotype. Importantly, the PDX models retained the histopathological characteristics of the lesion they were derived from (Fig. 5b). In parallel, the Brisken Laboratory at the Swiss Institute for Experimental Cancer Research, Ecole Polytechnique Fédérale de Lausanne (EPFL), focused on model generation from ER-positive breast cancers, including the ILC subtype^40,41^. Thanks to the UPTIDER collaboration, several PDXs models derived from 3 ILC patients have been established or are currently under investigation (Fig. 5c). Bioluminescence imaging of organs resected post-mortem from the animals revealed the presence of metastatic cells in multiple clinically relevant organs such as the brain, lungs, liver, kidney, adrenal gland, bones, gastro-intestinal tract, peritoneum and ovaries (Fig. 5c and 5d), indicating that rapid autopsy PDX models recapitulate the lobular metastatic disease features. Picrosirius red staining revealed the in situ and invasive growth of the lobular cells with the characteristic single-file lobular growth pattern in stroma enriched in fibrillar collagen (Fig. 5e). In addition, patient-derived organoids (PDO) development directly derived from these ILC PDX models is currently ongoing in the Derksen Laboratory at UMC Utrecht (Fig. 5f and 5g). Tumor models combined with longitudinal intravital imaging will additionally be created in the Laboratory of Intravital Microscopy and Dynamics of Tumor Progression at VIB-KU Leuven with a special focus on liver metastasis and investigation of histological growth patterns^37,42^. Organoid co-culture models from tumor and non-tumor UPTIDER samples to study the interaction dynamics between cancer cells and their host tissues will be established too. Finally, a patient-derived tumor fragment (PDTF) platform, where cellular composition and spatial organization from the original lesion can be maintained, has been set-up with the Thommen laboratory (Netherlands Cancer Institute)^39,43,44^. This platform will be used to test and compare multiple (new) treatment strategies, to directly link treatment responses to intra-patient inter-lesion heterogeneity and to provide new insights into response and resistance mechanisms.

After this promising start, the UPTIDER program permits robust downstream applications, including the collaborative development of diverse preclinical tumor models in parallel, which are crucial for preclinical drug evaluation, biomarker identification, biological studies, and personalized medicine strategies.

## Discussion

Post-mortem tissue donation programs can provide invaluable access to lesions inaccessible during patients’ lives. In metastatic breast cancer, the need for comprehensive sampling is high, as intra-patient multi-level heterogeneity is well-known and limits systemic treatment success. We report the set-up of a post-mortem tissue donation program for stage IV breast cancer patients and present the first results of scientific interest after the first years of enrollment.

In terms of patient inclusion, we aimed at focused enrollment according to the predefined research objectives and were able to include patients with less common subtypes of breast cancer, such as lobular, metaplastic and inflammatory breast cancer. Over two-thirds of patients that received an ICF (showing enough interest at initial approach to consider the study) ultimately decided to participate in the program, and this figure improved with mounting experience of the clinicians selecting and approaching the patients.

In terms of logistical set-up, all procedures revolved around a hypothesis-driven rather than a biobank-driven approach. Sample procurement protocols were focused on and tailored to the objectives of our program as well as to those from an extensive number of national and international collaborators. Preregistration of samples was carefully performed, leading to two thirds of metastases already having ready-to-go barcoded labels printed. The other one-third of metastases were either not expected based on previous clinical imaging, or the time between inclusion and death was too short to allow preparation (Pt2012). We were able to collect samples from a median of 31 and up to 90 metastases per patient, highlighting the extraordinary tumor tissue yield these programs can provide. Additionally, liquid biopsies and samples from non-invaded tissues were collected for future specific investigations. Most of the sample processing was done on-site during the tissue donation procedure, resulting in immediate final storage of all frozen liquid and tissue samples. We chose to put particular effort into electronic annotation of samples during the procedure itself too, including sample-specific registration of timepoints and storage location (see Methods). While this strategy importantly enhances the value and immediate usage of the samples and allows robust sample tracking, it comes at a cost of long autopsy durations. This parameter is only rarely reported, but our median of 6.5 h is likely to exceed that of other programs^28^. In contrast, median time between death and start of the autopsy in our study is only 3 h, which is shorter than or comparable to other programs^25–28,30,45–50^. Overall, all samples reported in this manuscript were collected within 13 h after death, which by all standards can be considered as rapid procurement. The tissue donation procedures were reliant on a 24 h/7d on-call trained team of ˜11 researchers and clinicians, a logistical feat on its own. We believe this was possible through motivation rooted in research interest, high involvement of all team members in project development as well as downstream analyses, internal funding agencies sustaining the program, and external funding agencies sustaining personnel costs.

Usually referred to as cold ischemia time, prolonged intervals between the surgical removal of a sample and its fixation are known to influence RNA quality measures and expression, albeit only slightly^51,52^. In the post-mortem setting, RNA quality has been assessed by several research groups, often on non-tumor tissues only^32,50,53,54^. In the few malignant tissues analyzed no association between RNA Integrity Number (RIN) values and PMI was seen^32^. These studies used surrogate markers of RNA quality (RIN is only a marker of ribosomal RNA stability) and none of them assessed samples taken repeatedly from the same patient, so the results could importantly have been biased by inter-individual variation in RNA quality. To our knowledge, only one study using repeated samples (from one failed and one non-failed heart) has been published so far^55^. In our study, repeatedly taken tumor and non-tumor tissues were considered, cooling was implemented, and actual sequencing data was considered. We showed a decline in assigned RNA reads and an in increase in ER protein expression with increasing time after death when samples were kept at room temperature. Importantly, this trend was not observed anymore when the samples were kept in a cold environment, in which setting all RNA and protein quality metrics remained stable. Of note, despite an overall stability of our quality metrics, variability is always observed within a time series at RNA and protein level. This can be explained, at least partially, by intra-tumor heterogeneity. Our results importantly help to fill the knowledge gap on post-mortem tumor tissue quality and reassure researchers who are collecting tissue samples within the first 11 h after death.

As another validation of sample quality, the engraftment of tissues collected through our tissue donation program has already resulted in the successful establishment of xenograft models. This included rare histological subtypes of breast cancer, as well as ER-positive disease which is generally less aggressive and thus more challenging to establish in mouse models. Histological evaluation showed that the characteristics of the original tumors were nicely retained, and some PDX models even exhibited metastatic capacity^56,57^. Ongoing efforts will lead not only to the establishment of more models from UPTIDER samples, but also to biological insights through experiments performed on these models.

Successful launching of the UPTIDER program now paves the way for multiple lines of research (Fig. 1). First, the molecular profiling of different types of liquid biopsies in combination with their respective tissue samples will allow for better exploitation of this minimally invasive liquid biopsy technique. Secondly, phylogenetic analyses on longitudinal and extensively collected post-mortem samples from the same patient will yield important insights into disease progression. Thirdly, mechanisms behind treatment sensitivity and resistance can be investigated on an individual lesion-level through multi-omic analyses and tumor model experiments. Next, micro-environment-specific features of the tumor, such as metabolic pathway reliance and immune infiltration, can be investigated and compared between organs. Heterogeneity of emerging biomarkers used as therapeutic target, a predictive or a prognostic tool can be evaluated in the metastatic setting^14^. While all these would not be possible on the limited number of samples that can be obtained from living patients, we acknowledge the limitations specific to the post-mortem setting, allowing only for single timepoint observations in a heavily pre-treated context. Finally, we aspire the leverage of this project to not confine itself to the field of breast cancer. By reporting on the set-up, feasibility and quality of our tissue donation program, we can importantly inform researchers in oncology and beyond, helping them to enable access to tissues irretrievable during the lives of patients.

## Methods

### Study design and inclusion procedure

UPTIDER is a monocentric post-mortem tissue donation program for patients with end-stage breast cancer (NCT04531696, local ethics number: S64410, approval 30^th^ November 2020 by ethical committee research UZ/KU Leuven). Written informed consent is obtained from all participants and all relevant ethical regulations including the Declaration of Helsinki are complied with. Different substudies are described, focusing on specific research questions (e.g. liquid biopsies, xenograft establishment) or on specific patient subpopulations (ILC, inflammatory breast cancer, male breast cancer). All adult patients, regardless of gender, with metastatic breast cancer (either de novo or relapse) in their last line(s) of treatment are eligible for inclusion, provided that they are either treated at University Hospitals Leuven or referred to it specifically for the project. Additionally, patients with a hereditary cancer syndrome conferring a high risk of developing breast cancer and with at least one malignancy (of any organ) diagnosed at time of inclusion are eligible too; so far no patients have been recruited based on this inclusion criterion only. Exclusion criteria are the presence of transmissible diseases that can form a risk to the health of researchers handling the body or patient samples (e.g. HIV, HCV, tuberculosis), and place of residence outside the area covered by the transport company (Belgium–Netherlands–Luxemburg, and some areas of nearby countries).

Patients are notified of the program by their treating oncologist at a point in time during their disease course that is deemed appropriate by that physician. A leaflet with information in layman terms is offered. In case the patient shows interest, a member of the UPTIDER research team provides the patient with more detailed information and an informed consent form (ICF). In the ICF, patients can optionally choose to donate samples from the head and neck area, to allow the collection of samples during life when a drainage is clinically indicated, and to inform the family in case a germline mutation of clinical relevance is found. If the patient agrees to participate, the signed form is either sent back to the researchers by post, to allow for the preparation of the inclusion sampling prior to the patient’s next visit to the clinic, either returned by the patient to the physician. If needed, further discussions with the patient and the family are planned. Unless the patient objects to this, the general physician is informed about the patient’s participation in the project.

### Sample collection at inclusion

Upon inclusion, a blood draw is performed for the following: (i) circulating tumor cell (CTC) isolation and counting using Cellsearch® protocols; (ii) peripheral blood mononuclear cell (PBMC) isolation using SepMate™ tubes and Histopaque®-1077 density gradient, with cryopreservation in fetal bovine serum (FBS)-10% dimethyl sulfoxide (DMSO) freezing medium; (iii) plasma isolation (collection in 9 mL EDTA tube, 15 min centrifugation at 4 °C, 3000 × g) for different purposes (e.g. cell-free DNA (cfDNA) extraction, proteomics profiling) and storage of the remaining leukocyte cell pellet for later germline DNA extraction; (iv) clinical analyses such as endocrine assays, serology testing for transmittable diseases HIV, HBV, HCV and CMV (as part of exclusion criteria assessment and safety requirements for sample handling). This blood draw can be repeated throughout the follow-up period preceding death at time points of interest, e.g., at clinical progression. Additionally, saliva is collected and frozen without processing and urine supernatant and cell pellet are collected and stored (addition of 0.5 M EDTA, then 10 min centrifugation at 4 °C, 2400 × g). In case the patient undergoes diagnostic or therapeutic sampling of other body fluids after inclusion (such as ascites, pleural fluid, cerebrospinal fluid), wherever possible supernatants and cell pellets are collected (15 min centrifugation at 4 °C, 3000 × g). Historical tissue samples of the primary tumor, if available, are requested from the pathology biobank of the hospital where they are stored. DNA extracted pre-mortem from tumor tissue and/or plasma (cfDNA) in the clinical setting, if available, is requested from the department of human genetics of University Hospitals Leuven. If available, prospective blood samples (plasma/extracted genomic DNA), collected at primary diagnosis and at first distant relapse for translational research by the Multidisciplinary Breast Centre of University Hospitals Leuven (Biobanking project S63773), are also requested.

### Data collection at inclusion

Data collected at inclusion encompasses medical and familial history, cancer characteristics at diagnosis, location and timing of the metastases, anticancer treatments and their responses on a patient-level as well as on an individual lesion-level, histopathological characteristics of the primary and -where available- of the metastases, relevant laboratory results (tumor markers, DNA or RNA sequencing results). Imaging performed close to the date of inclusion is carefully assessed, as this will serve as the basis for the tissue donation preparation. All data collected at inclusion is registered in a specifically designed electronic case report form (eCRF) using REDCap electronic data capture tools hosted at University Hospitals/KU Leuven^58,59^. REDCap (Research Electronic Data Capture) is a secure, web-based software platform designed to support data capture for research studies, providing (1) an intuitive interface for validated data capture; (2) audit trails for tracking data manipulation and export procedures; (3) automated export procedures for seamless data downloads to common statistical packages; and (4) procedures for data integration and interoperability with external sources.

### Preparation of the tissue donation

Data from the medical file of the patient and imaging performed close to inclusion are used to construct an individual tissue donation plan for each patient. Standardly included samples liquid biopsies from all body fluids and non-invaded samples of the brain, breast, lung, heart, liver, kidney, and adipose tissue (retroperitoneal, subcutaneous and from the breast). For all patients, known tumor lesions are added to the donation plan, along with adjacent normal tissue samples, where possible. Previously locally treated lesions are also registered, including irradiated breast/thoracic wall tissue and tissue from irradiated metastatic sites. Within this tissue donation plan, for each sampling site different processing methods are encoded. Standard processing for tissue samples results in three mirrored samples: one formalin-fixed paraffin-embedded (FFPE), one fresh frozen (FF) and one fresh frozen in optimal cutting temperature compound (FF_OCT). Depending on the size of the lesion and scientific interest, additional fresh samples (FRESH), samples frozen in carboxymethylcellulose (FF_CMC) and samples frozen slowly in freezing medium (FF_DMSO) are encoded. For very large lesions multiregional sampling can be planned. From this tissue donation plan sample records are created upfront in our lab management system LabCollector^©^. In conclusion, individual samples are preregistered before the autopsy in a structured way, including information on the patient they will be taken from, the location of sampling (using ICD-O-3 organ codes^60^), the type of tissue (normal versus tumoral versus previously irradiated), and the intended processing and storage method.

### Tissue donation procedure

Patients remain in passive follow-up in the study after inclusion. At time of death, the UPTIDER team is notified straight away by the patients’ family or caregivers via a central phone number (24 h/7d). In case the patient passes away at home, the transport company under contract for the program will pick up the body as soon as possible and transport it to the morgue of University Hospitals Leuven. If logistically feasible, a post-mortem whole-body MRI is performed before the start of the autopsy.

After registration of general information (weight, body temperature, external examination of the body) and performance of a rapid antigen test for SARS-CoV-2, the autopsy procedure will start. First, cerebrospinal fluid (CSF) is collected through a puncture of the cisterna magna with the patient in prone position. The patient is then turned to supine position and, in case an implanted central port (port-a-cath) is present, blood samples are drawn through it. Next, the body is opened through a T-incision, and pleural fluid (left and right separately), ascites, urine, peripheral blood (femoral vein or iliac vein preferably), pericardial fluid, central blood (intracardial) and bone marrow samples are collected. Liquid samples are centrifuged and stored as supernatants and cell pellets (blood and urine same settings as pre-mortem (cfr supra); pleural fluid, pericardial fluid, ascites and CSF 15 min centrifugation at 4 °C 3000 × g; bone marrow frozen as such). After all liquid sampling has been completed, tissue sampling starts. Tissue samples are taken in order of priority set for that patient and depending on feasibility in terms of organ location. For downstream techniques very sensitive to nucleic acid degradation, such as single nuclei RNA sequencing, quick FF biopsies (snap frozen in liquid nitrogen) can be taken in situ first, before removal of any organs. Since implementation of organ cooling (May 2022), organs are subsequently kept (as individual organs or as organ blocks) in iced water (4 °C–10 °C) until dissection. All solid organs that are retrieved are sliced completely to evaluate known lesions, and to discover possible unknown lesions. Standardly, from each macroscopically apparent malignant lesion three mirrored samples are taken from the central part of the lesion, three from the interface (where possible), and three from the adjacent normal tissue (1 FFPE, 1 FF and 1 FF_OCT for each sampling location). For small lesions, FFPE and FF_OCT samples are prioritized. Multiregional sampling (from different regions within the same lesion) is performed for large lesions. For very large lesions or very diffusely infiltrated organs, any left-over malignant tissue is frozen as a whole in a vacuum bag. From macroscopically diffusely invaded livers, samples are taken from each anatomical segment. Malignant-looking lymph nodes are each sampled and encoded individually. For lymph nodes in the cervical region, careful dissection from the T-incision in cranial direction allows sampling without the need for additional skin incisions. Slices of heavily invaded organs are frozen in vacuum sealed bags for future research purposes. For the procurement of bone lesions, we especially harvest lesions from the anterior part of the spine and skull. Bone lesions that might lead to delay in the procedure and/or disfigurement of the body are avoided. For lesions in the vertebrae, the intervertebral disks are incised with a knife. The pedicles are cut through with the saw to remove the vertebral body. The vertebral body is then further incised with the saw to access the metastatic deposits. Soft tumor parts are curetted for snap freezing or immediate fresh processing. This is only possible for osteolytic bone lesions. The rest material is put on formalin for 2 days, and after fixation sliced with a saw in 4 mm slices. These are then decalcified with EDTA to preserve antigenicity and DNA integrity. If the patient consented for this and if invasion is known or suspected, brain samples are taken after opening of the skull using a standard approach. As stipulated in the section on the preparation of the tissue donation, non-tumor samples are taken from different organs, preferably at the end of the autopsy. These will serve either for detection of occult metastasis, as controls for later interpretation of findings in tumor tissues, to assess mechanisms of treatment toxicity, or to help determine the direct cause of death. Every sample’s QR code is scanned at time of freezing (if applicable) or storage in other media, to later calculate the exact sample-specific ischemia time. The QR code is scanned again when the sample is being moved to its final storage position in a sample box or container, to register its location. This scanning additionally allows the discrimination between samples that were planned to be taken but were eventually not (will be archived in our lab management system) and those that were indeed taken and stored.

To accomplish this extensive sample collection and registration, the autopsy team consists of different members each with their own role during the procedure. One team leader guides the autopsy and the team from beginning till end and makes sure priorities are set and followed (coordinator). One or more team members with expertise in forensic/clinical autopsies open the body, retrieve all liquid samples, dissect the organs, procure the tissue samples, and close the body (prosectors). They are assisted by morgue technicians when available. One team member cleans equipment in between tissue samples to avoid cross-contamination of nucleic acids. Two or more team members process liquid samples according to their specific standard operating procedures, receive the tissue samples in their respective recipients and process them correctly (sample processors). Two or more team members register new/unexpected samples in the sample management system, register the sample-specific time points (time of freezing) for all samples as well as the final storage location, take macroscopical images during the autopsy of organs and samples retrieved (from which ‘time of sampling’ is later derived), and annotate and register all other information needed for accordance with biobanking regulations.

At the end of the autopsy, the body is reconstituted expertly. Within 24 h after the patient’s death, the body will be transported to the undertaker of the patient’s choice.

### Extensive liquid and tissue sampling during the rapid autopsies

For this section of the results, metastases per patient were counted as samples of non-breast tissue with histologically confirmed invasion on tissue slides (4 µm from FFPE samples) stained with hematoxylin and eosin (H&E) and subsequently scored for tumor cellularity. We excluded doublets of the same lesion (e.g. samples from the repeated sampling experiment, multiregional sampling within the same lesion).

### Repeated sampling experiment – sample acquisition

A repeated sampling experiment was set up to specifically evaluate the effect of increasing time after death on sample quality. Selected non-tumor and large tumor tissues (with enough material available to collect for this experiment beside routine sampling) were sampled repeatedly at 1.5-hour time intervals during the autopsy and stored as FFPE, FF and FF_OCT samples. The experiment was initiated in June 2021 and concluded in January 2023, collecting extra samples at room temperature from 7 patients (Pt2004, Pt2006, Pt2007, Pt2008, Pt2009, Pt2010, and Pt2011) and, in a second phase, at room temperature and cooled between 4 °C and 10 °C from 6 patients (Pt2018, Pt2020, Pt2023, Pt2024, Pt2025, and Pt2027). Of note, when both conditions were available, the sample considered for time point 1 was the same for both conditions.

### Repeated sampling experiment – protein expression

Protein expression of ER, PR and KI67 was assessed on the FFPE samples with confirmed presence of tumor cells. Immunohistochemical staining (IHC) was performed for ER (antibody clone EP1, DAKO, RTU, CE-IVD), PR (PgR 1294, DAKO, RTU, CE-IVD) and KI67 (MIB1, DAKO, RTU, CE-IVD). ER and PR were scored according to the Allred scoring system^61^. KI67 score was determined by estimating the average percentage of positively staining nuclei among the total number of staining nuclei across the entire sample.

### RNA extraction and analysis

RNA was extracted from the FF samples collected specifically for the repeated sampling experiment. Invitrogen™ TRIzol™ reagent was used to isolate RNA from the tissue specimen. 0.2 mL of chloroform (Sigma Aldrich - C2432) was added per 1 mL of TRIzol™ Reagent, and a subsequent step of centrifugation (15 min at 12,000 × g at 4 °C) was carried out to separate the lysed tissue mixture. The colorless upper aqueous phase that contains the RNA was transferred to a new eppendorf tube. To co-precipitate and visualize the RNA pellet, 1 µL of Genelute – LPA (Sigma Aldrich - 56575) was added to the transferred aqueous phase, followed by 0.5 mL of isopropanol per 1 mL of TRIzol™ Reagent to precipitate the RNA. Following a subsequent step of centrifugation (10 min at 12,000 × g at 4 °C), the RNA pellet was washed twice with 75% ethanol, allowed to air dry for up to 10 min and then solubilized in nuclease-free water. Extracted RNA was quantified using NanoDrop™ One spectrophotometer. RNA samples were further checked for quality and quantity by using the Agilent RNA 6000 nano kit (ref. no. 5067–1511) on an Agilent 2100 Bioanalyzer system to get the RIN values. The extracted RNA was subsequently sequenced using the lexogen protocol (3’ mRNA FWD Quantseq) for Illumina NovaSeq6000. The RNA reads were mapped to the GRCh38 reference genome (primary assembly, dated 08.06.2022) using the STAR aligner^62^ (v2.7.10a) and the transcripts were annotated using gencode (v40 for GRCh38.p13). Gene counts were calculated using the subread package^63^ (v2.0.3) and normalized using the variance stabilizing transformation method from the DESeq2 package^64^ (v1.34.0).

### Statistical analysis: repeated sampling experiment

Quality metrics were defined as: (i) sequencing metrics: number of reads assigned to genes, and number of expressed genes, (ii) transcriptomic metrics: a selection of 15 gene signatures covering different aspect of the tumor and its micro-environment such as breast markers, proliferation, immunity, angiogenesis, hypoxia, and stroma (references and gene lists in Supplementary Table 3), (iii) protein metrics: percentage of tumor cells expressing ER, PR and KI67 proteins evaluated by IHC. Gene signatures were computed as a weighted average of the normalized gene expression, with the weights being set to −1 or 1 for negative or positive coefficients respectively^65^. Sample-specific post-mortem interval (ssPMI) was defined as the time between death of the patient and fixation of the sample in hours. Associations between quality metrics and ssPMI were assessed by linear regressions for longitudinal data, with quality metrics scores as dependent variable, time as independent variable and accounting for the clustering of the data by patient and organ using the generalized estimating equation method using the glmtoolbox R package (v0.1.4). Three nested models - with constant, linear and non-linear relationship - were compared using ANOVA testing strategy and the best model was retained. Non-linearity was rendered by a restrict cubic spline with three knots. All tests were performed by the Wald test on regression coefficients. *P*-values are two sided. All analyses were performed in R version 4.2.1.

### Patient-derived xenograft development

#### TRACE PDX platform

Fresh solid tumor samples obtained through UPTIDER are transported to the TRACE PDX platform headed by Prof. Leucci in a transport container with Transport medium (RPMI 1640 medium, 500 mL; containing Penicillin/Streptomycin, Gentamicin and Fungizone). In case no direct implantation is possible because of the timing of the autopsy or unavailability of mice, samples are put in freezing medium (90% FBS with 10% dimethylsulfoxide (DMSO)) and slowly frozen to −20 °C and subsequently to −80 °C in a freezing container where they are stored until thawing for implantation. In case cerebrospinal fluid is retrieved for implantation, it is centrifuged at 300 × g for 10 min at 4 °C, most of the supernatant is removed, and the cell pellet is then resuspended in 0.5–1 mL of supernatant and transported on ice. All subsequent procedures are performed under a laminar flow hood. For implantation in immune-compromised mice, the tumor tissue is divided into small pieces (3 × 3 × 3mm) and transferred to a sterile cryotube containing Matrigel kept on ice until implantation. A representative tissue fragment (5 × 5 × 5mm) is also snap-frozen for subsequent molecular characterization of the model. Mice (NMRI nude or NOD-scidIL-2 Rγnull) are anesthetized with an intraperitoneal injection (IP) of a mixture of ketamine, medetomidine and saline solution. The surgical site is shaved with enough border area to keep hair from contaminating the incision and disinfected with ethanol 70% on a sterile gauze. Anesthetized mice are placed on a heating pad (37 °C) to prevent hypothermia and eyes are protected from drying during anesthesia with Alcon eye gel 10 g if surgical procedures lasts more than 10 min. Surgery is performed under a laminar flow. The human tumor fragment is implanted in the mammary fat pad and the wound is closed with a Michel suture clip. Post-operative analgesia consisting of a buprenorphine solution is injected by subcutaneous administration and anesthesia is reversed with Atipamezole solution, subcutaneously. The animals are allowed to fully recover from anesthesia on a heating pad and are closely monitored for the first 2–3 h post-surgery. Then, the first days after implantation the mice are regularly checked for any signs of disease, body weight, or infection of surgical incision. Mice are sacrificed when they reach humane endpoints or when the tumor is large enough for biobanking. The harvested tumor is either xenografted into another set of mice and/or stored for further analyses and later reimplantation. A model is considered established after passaging into three generations of mice and when the conservation of human morphology and genealogy is confirmed by quality control analyses (H&E and SNP fingerprinting). Mouse experiments were performed according to the regulations of the Federation of European Laboratory Animal Science Associations and approved by the local ethical committee (P164/2019; P189/2020). **Brisken Laboratory**. *Animal Experiments*. NOD.Cg-Prkdcscid Il2rgtm1Wjl/SzJ (NSG) were purchased from Charles River. NSG mice were maintained and handled according to Swiss guidelines for animal safety with a 12-h-light-12-h-dark cycle, controlled temperature 22 ± 2 °C, and controlled humidity 55% ± 10 °C with food and water ad libitum. All experiments were performed under the protocol VD1865.5 and VD3795, approved by Service de la Consommation et des Affaires Vétérinaires, Canton de Vaud, Switzerland. *Tissue Handling and Digestion*. Tumor samples obtained through UPTIDER were transported on ice to EPFL, Switzerland, in DMEM F-12 medium (catalog number: 31331028) Thermo Fisher Scientific (Life Technologies) supplemented with 2% penicillin/streptomycin (including 5000 unit/ml penicillin and 5000 g/ml streptomycin) and 1% antibiotic/amphotericin B (including 10,000 units/ml penicillin, 10,000 μg/ml of streptomycin, and 25 μg/ml of amphotericin B). In case cerebrospinal fluid was retrieved for transplantation, it was transported on ice without centrifugation. Tissue samples were processed by mechanical and enzymatic digestion, and lentiviral transduction (GFP-luc2) was performed as described^40,66^. Lentiviruses lenti-ONE GFP-2A-Luc2 were designed and purchased from GEG Tech in partnership with Dr. Nicolas GrandchampCells were counted using trypan blue (Bio-Rad, Cat. No.1450021) with dual-chamber cell counting chamber slides (Bio-Rad, Cat. No. 145-0011) in an automated cell counter (Bio-Rad TC20). All experimental procedures were performed under sterile conditions in a laminar flow hood under general standard EPFL operation procedures for the biosafety level 2 laboratory. *Intraductal Injections*. Transplantations were performed into the milk ducts of NOD.Cg-Prkdcscid Il2rgtm1Wjl/SzJ (NSG) 8–12-week-old female mice. Mice were anesthetized by intraperitoneal injection of 200 µl of 10 mg/kg xylazine and 90 mg/kg ketamine (Graeub). Intraductal injections of single-cell suspensions were generated by injecting 186 × 10^5^ to 125 × 10^6^ cells into the teats of 8–12-week-old NSG female mice as previously detailed^40,41^. The animals were allowed to recover from anesthesia on a heating pad and were closely monitored for the first 2–3 h post-surgery. *Tumor Growth and Metastasis Analysis*. Tumor growth of individual xenografted glands was monitored by in vivo imaging system (IVIS, Caliper Life Sciences). Briefly, post intraperitoneal administration of 150 mg/kg luciferin (cat# L-8220, Biosynth AG) with an insulin syringe 0,33 mm (29 G) x 127 mm, mice were first anesthetized in the induction chamber (O_2_ and 2% isoflurane) and then placed inside the IVIS machine where eyes are protected from drying during anesthesia with Viscotears Augengel. Images were acquired approximately 14–18 min after luciferin injection and analyzed with Living Image Software (Caliper Life Sciences, Inc.). For metastasis detection, mice were first injected with 300 mg/kg luciferin for 8 min and then injected with 1 ml of 10 mg/kg xylazine and 90 mg/kg ketamine. Resected organs were imaged approximately 14–18 min after luciferin injection. Mammary glands and tissues were fixed in 4% paraformaldehyde (PFA) overnight at 4 °C or snap-frozen in liquid nitrogen for RNA and protein extraction. Stereoscope images were taken with a Leica M205 FA Fluorescence Stereo Microscope equipped with a LEICA DFC340FX camera. **Derksen Laboratory**. *Cell and organoid culturing*. The human ILC organoid model MetsUM-01-C1991M14 was created from patient PT2011 from the UPTIDER program from mouse transplanted tumors by resuspending dissected, and mechanical/enzymatic digested cells in 40 µL of cold 10 mg.ml Cultrex growth factor reduced Basement Membrane Extract (BME). The cell suspension was distributed in a pre-warmed 24-well plate (Greiner) over four drops (10 µL per drop) per well. Culture medium was optimized for this model, we used DMEM-F12 supplemented with 1% Penicillin/Streptomycin, 10 mM HEPES, 5 ng.ml Epidermal Growth Factor (Peprotech), 10 µg.ml Insulin (Sigma), 0.5 µg.ml Hydrocortisone (Sigma), 70 µg.ml Bovine Pituitary Extract (ThermoFisher), and 10 µM Y-27632. Upon full gelation, 500 µL of optimized medium was added, and the plates were transferred to humidified 37˚C / 5% CO2 incubators. Medium was refreshed every 3 days. For passaging, organoids were resuspended in 1 ml of cold Advanced DMEM-F12 medium supplemented with 1% Penicillin/Streptomycin, 2 mM L-Glutamine, and 10 mM HEPES and were dissociated by mechanical sheering. Organoid structures were resuspended in cold BME, reseeded in a 1:3 drop ratio to preserve high density, and were cultured in separate plates from other organoid lines to prevent cross-contamination. For immunofluorescence microscopy, organoid cultures were collected and washed in cold PBS containing Mg2+/Ca2+, before being fixed with 4% paraformaldehyde (PFA)^67^. *Antibodies and reagents* The following primary antibodies were used for Immunofluorescence: monoclonal TRITC-conjugated mouse E-cadherin (clone 36, 1:50; BD560064; BD Biosciences), and TROMA-I rat cytokeratin 8 (TROMA-I, 1:200; Developmental Studies Hybridoma Bank). *Microscopy* Representative differential interference contrast microscopy (DIC) images were acquired by using a 103 objective on an EVOS M5000 Imaging System. Images were white balanced (after preliminary auto-exposure). Immunofluorescence microscopy samples were prepared as described previously^67^. Briefly, PDO-PDX cultures were in gel (in a 24- or 48-well culture plate) fixed in 4% paraformaldehyde in PBS at pH 7 for 20 min and permeabilized with TBS-T (TBS—0.1% Triton X-100) at room temperature. Cells were blocked in PBS-T supplemented with 10% BSA for 1 h at room temperature. Primary antibodies were incubated overnight at 4˚C, rocking, in blocking solution. Wells were then washed three times 15 min with PBS and incubated with secondary antibodies in blocking solution overnight, rocking at 4˚C. After three washes in PBS, cells were stained with 2 mg/mL DAPI (Sigma, D9542) for 5 min. Imaging was conducted on a Zeiss LSM880 confocal microscope. Images were acquired and analyzed with ImageJ software.

### Reporting summary

Further information on research design is available in the Nature Research Reporting Summary linked to this article.

### Supplementary information

Supplementary Material

Reporting Summary

## Data Availability

All relevant data are available from the authors. The clinical, histological and processed RNA sequencing data used in this manuscript is provided in a code capsule in the CodeOcean repository (10.24433/CO.8724699.v1)^68^. Additionally, the raw sequencing data (FASTQ files) are deposited in the EGA repository (accession number: EGAS50000000224). Samples including tissue and body fluids are available for research pending EC approval of the specific project and signed material transfer agreement (MTA) with UZ/KU Leuven. Please contact christine.desmedt@kuleuven.be for further information.

## Code availability

The R code along with the processed data required to reproduce the analysis is provided in a code capsule in the CodeOcean repository (10.24433/CO.8724699.v2)^68^.
