# Supplementary material for: Rapid autopsies to enhance metastatic research: the UPTIDER post-mortem tissue donation program
Source: NPJ Breast Cancer. 2024 Apr 24;10:31. doi: 10.1038/s41523-024-00637-3 (PMC11043338; doi:10.1038/s41523-024-00637-3)
Supplement: Supplementary file 1 — Supplementary Material [file 41523_2024_637_MOESM1_ESM.pdf]

# Supplementary Appendix

## Rapid autopsies to enhance metastatic research: the UPTIDER post-mortem tissue donation program

### Table of Contents

***Supplementary Figures*** ........................................................................................... ***2***

**Supplementary Figure 1** ........................................................................................................ **2**

**Supplementary Figure 2** ........................................................................................................ **3**

**Supplementary Figure 3** ........................................................................................................ **18**

**Supplementary Figure 4** ........................................................................................................ **36**

**Supplementary Figure 5** ........................................................................................................ **36**

**Supplementary Figure 6** ........................................................................................................ **38**

***Supplementary Tables*** ........................................................................................... ***41***

**Supplementary Table 1** ........................................................................................................ **41**

**Supplementary Table 2** ........................................................................................................ **42**

**Supplementary Table 3** ........................................................................................................ **43**

# Supplementary Figures

## Supplementary Figure 1

Histogram (left) and violin plots (right) of the post-mortem interval (PMI, time between death and start of the autopsy) and duration of autopsy for patients undergoing autopsy within the UPTIDER program up until January 15th 2023.

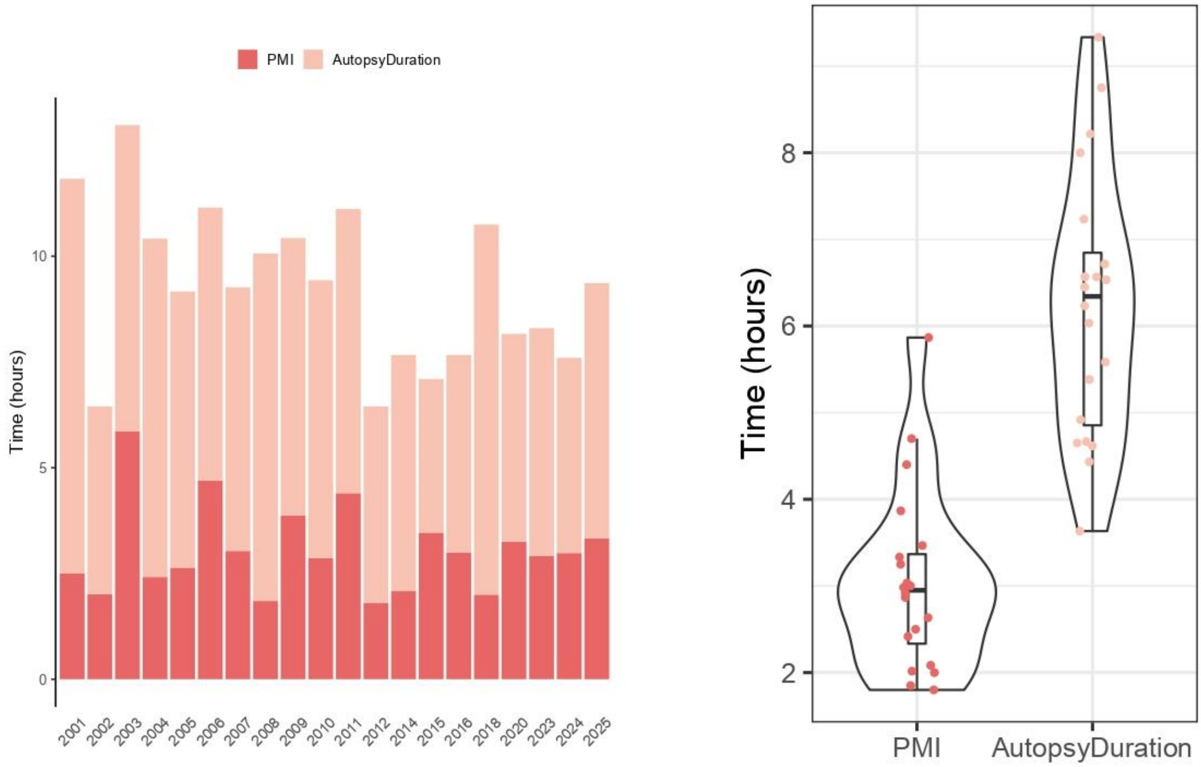

## Supplementary Figure 2

Trajectories of sequencing metrics and transcriptomics signatures in non-tumor tissues.

Abbreviations: AR: number of assigned reads to genes, genecount1: number of expressed genes, N: non-tumor tissue, P: tumor tissue, cold: samples cooled between 4°C and 10°C, RT: room temperature, samplePMI: sample specific post-mortem interval.

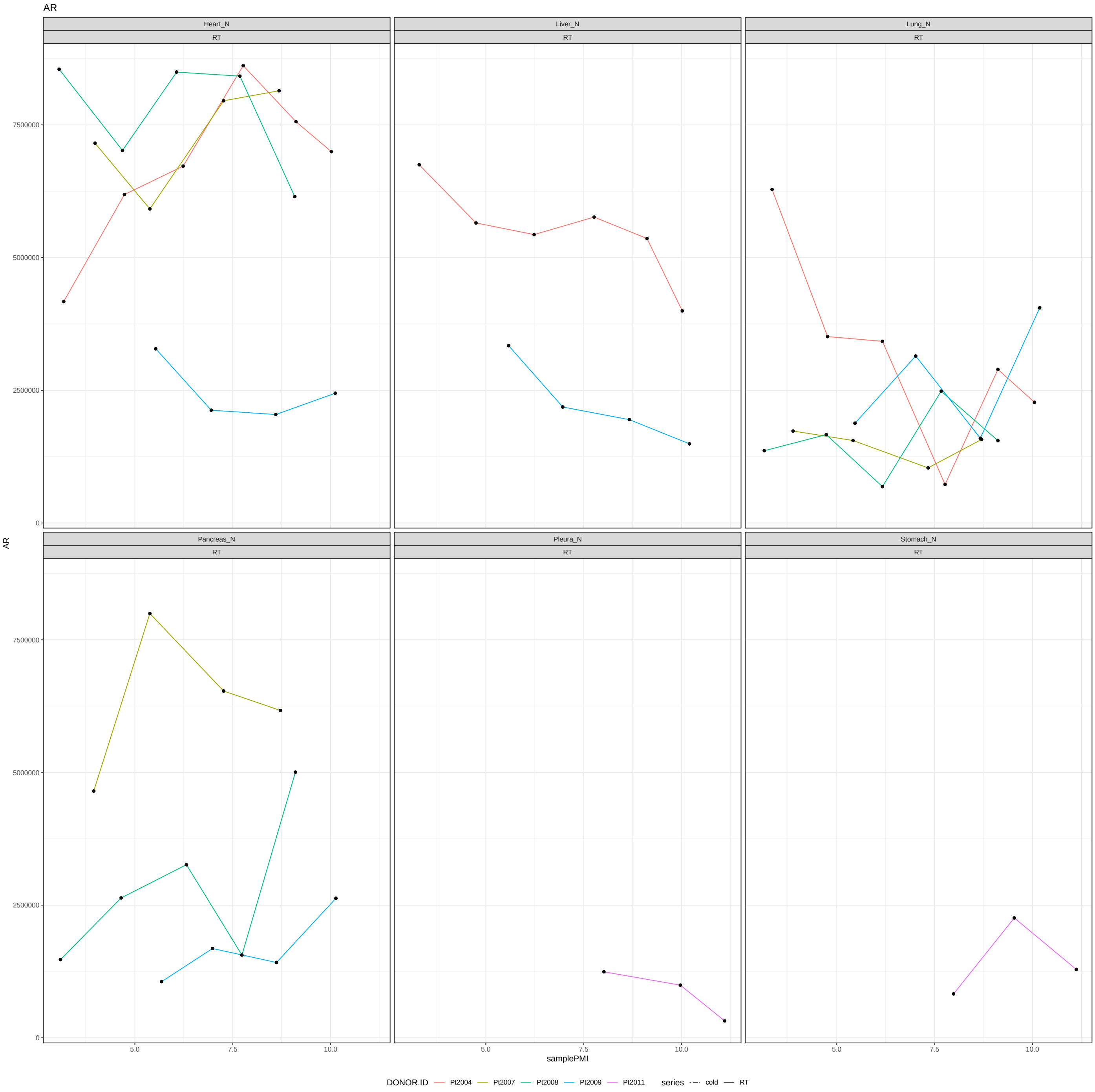

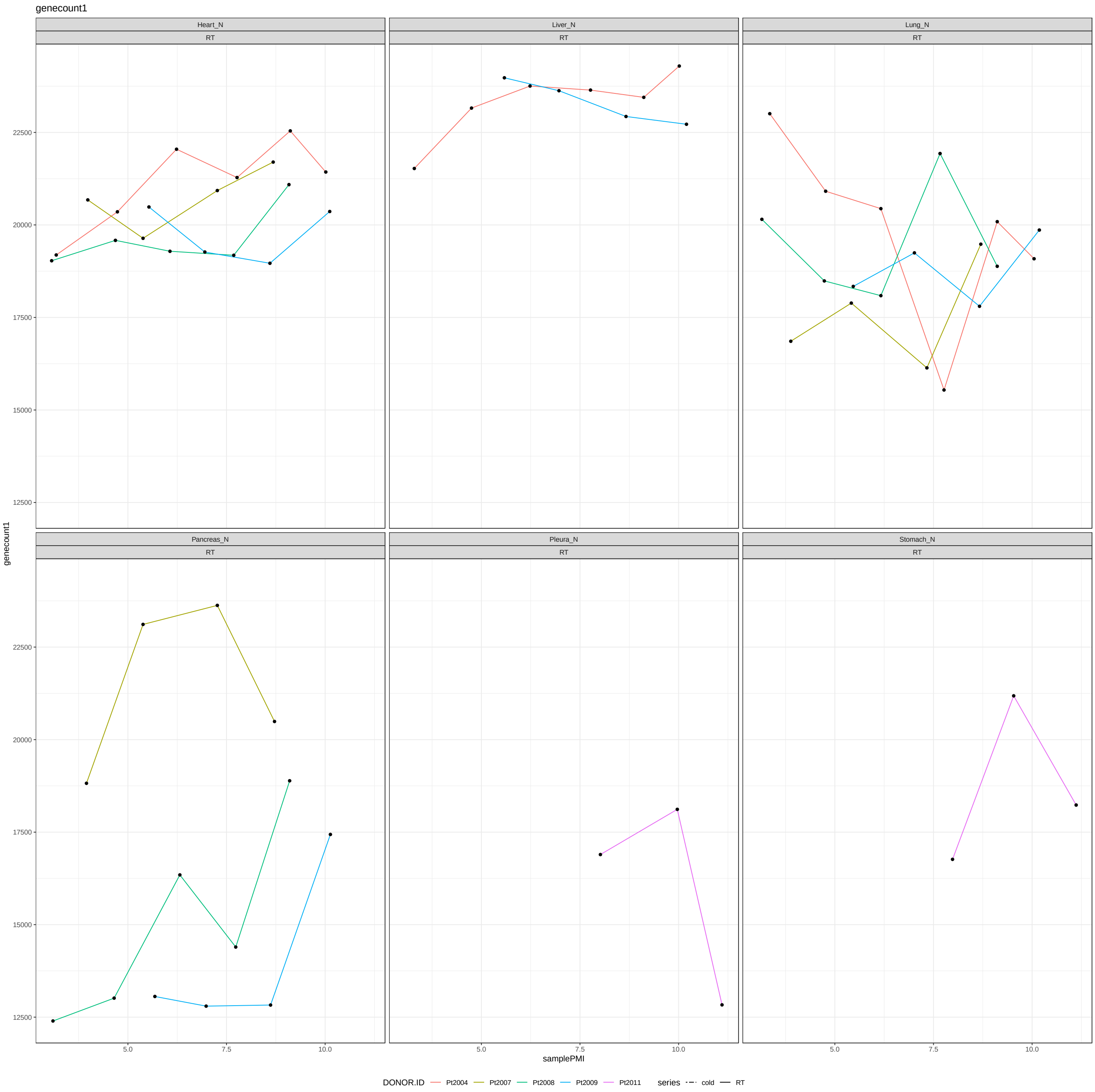
genecount1
Heart_N
RT
Liver_N
RT
Lung_N
RT
Pancreas_N
RT
Pleura_N
RT
Stomach_N
RT
genecount1
12500
15000
17500
20000
22500
5.0
7.5
10.0
samplePMI
DONOR.ID
Pt2004
Pt2007
Pt2008
Pt2009
Pt2011
series
cold
RT

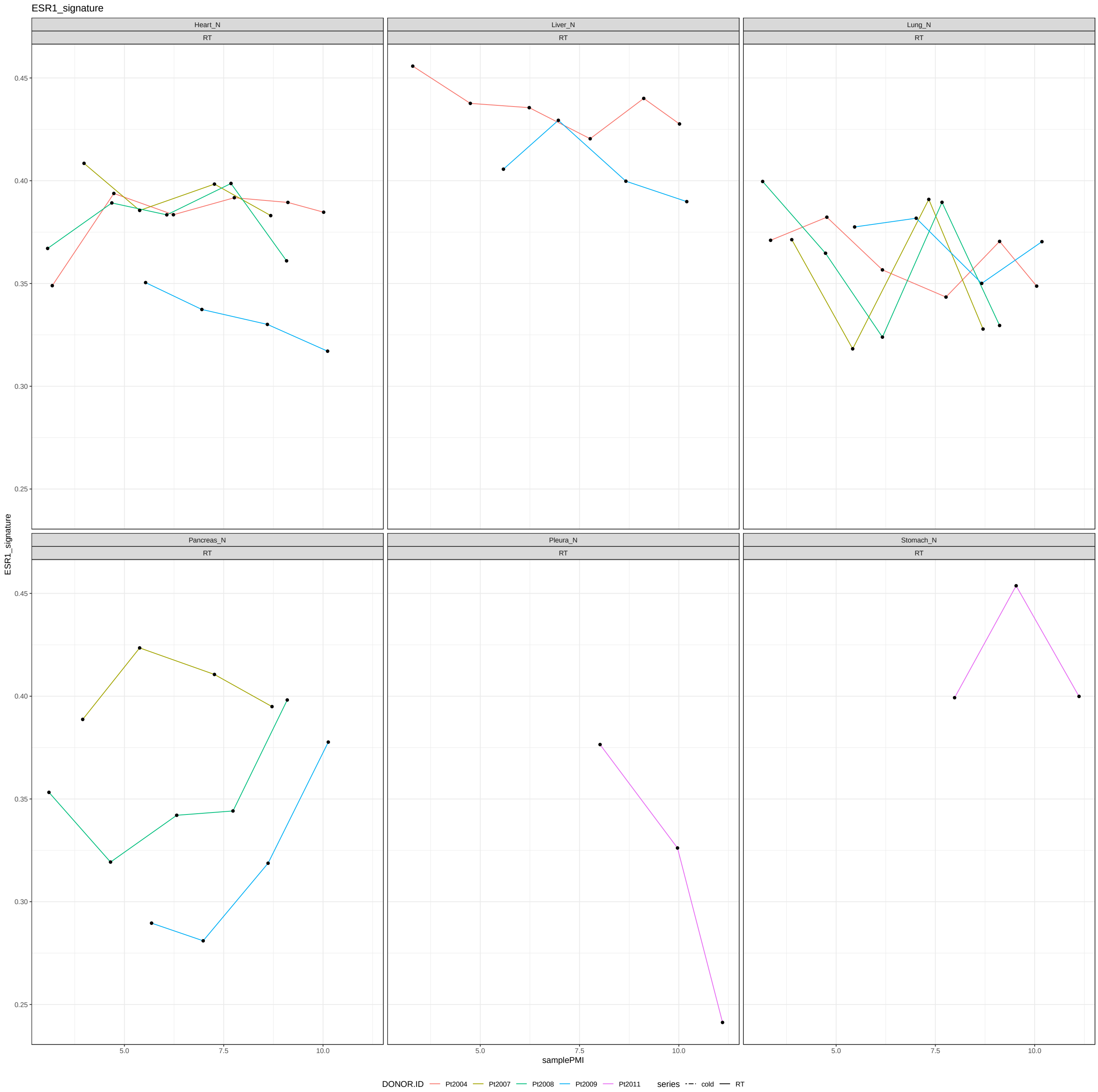

ESR1_signature
Heart_N
RT
Liver_N
RT
Lung_N
RT
Pancreas_N
RT
Pleura_N
RT
Stomach_N
RT
ESR1_signature
0.45
0.40
0.35
0.30
0.25
5.0
7.5
10.0
samplePMI
DONOR.ID
Pt2004
Pt2007
Pt2008
Pt2009
Pt2011
series
cold
RT

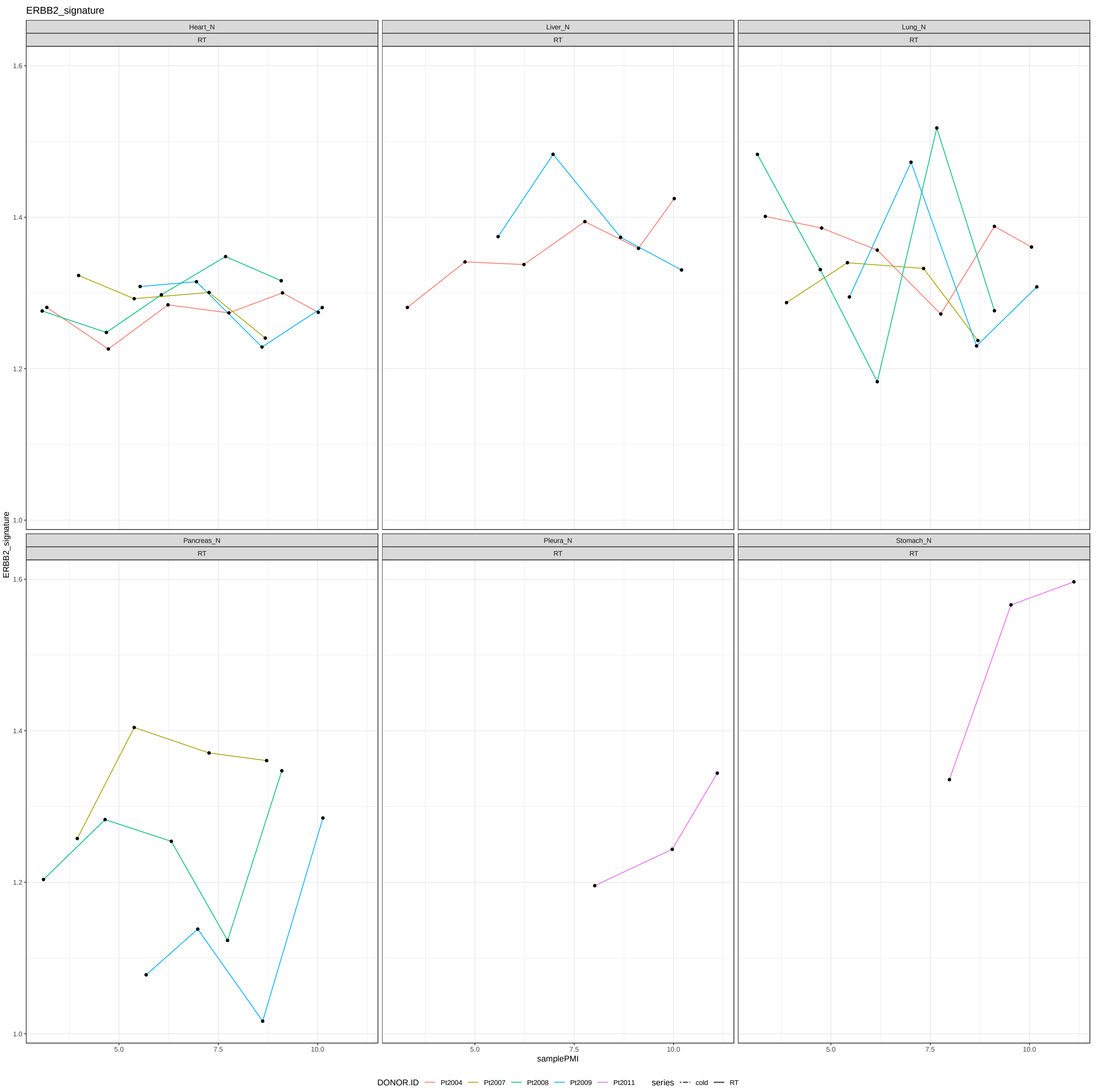

ERBB2_signature
Heart_N
Liver_N
Lung_N
Pancreas_N
Pleura_N
Stomach_N
RT
1.6
1.4
1.2
1.0
5.0
7.5
10.0
ERBB2_signature
samplePMI
DONOR.ID
Pt2004
Pt2007
Pt2008
Pt2009
Pt2011
series
cold
RT

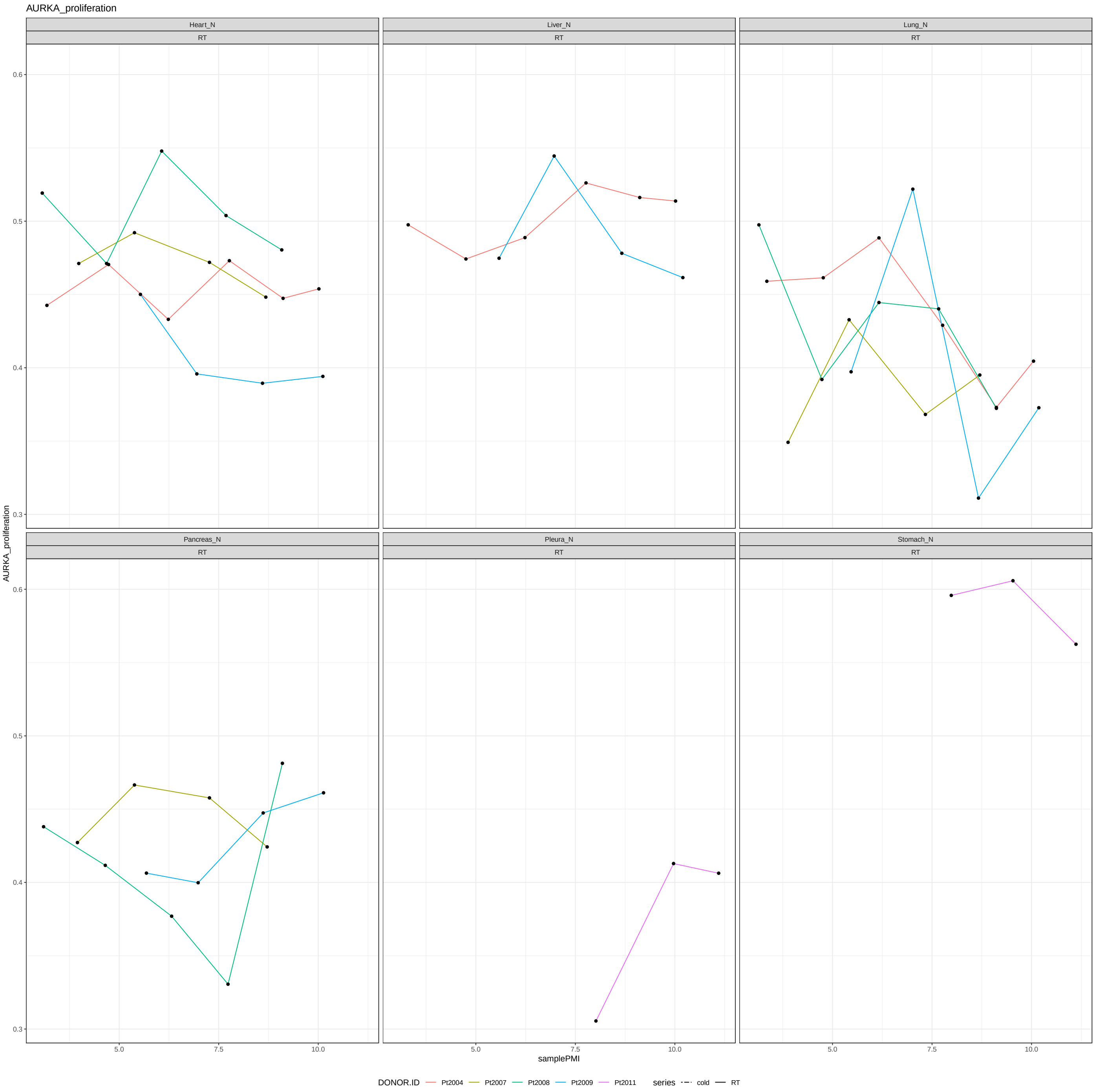
AURKA_proliferation
Heart_N
Liver_N
Lung_N
Pancreas_N
Pleura_N
Stomach_N
RT
AURKA_proliferation
0.3
0.4
0.5
0.6
5.0
7.5
10.0
samplePMI
DONOR.ID
Pt2004
Pt2007
Pt2008
Pt2009
Pt2011
series
cold
RT

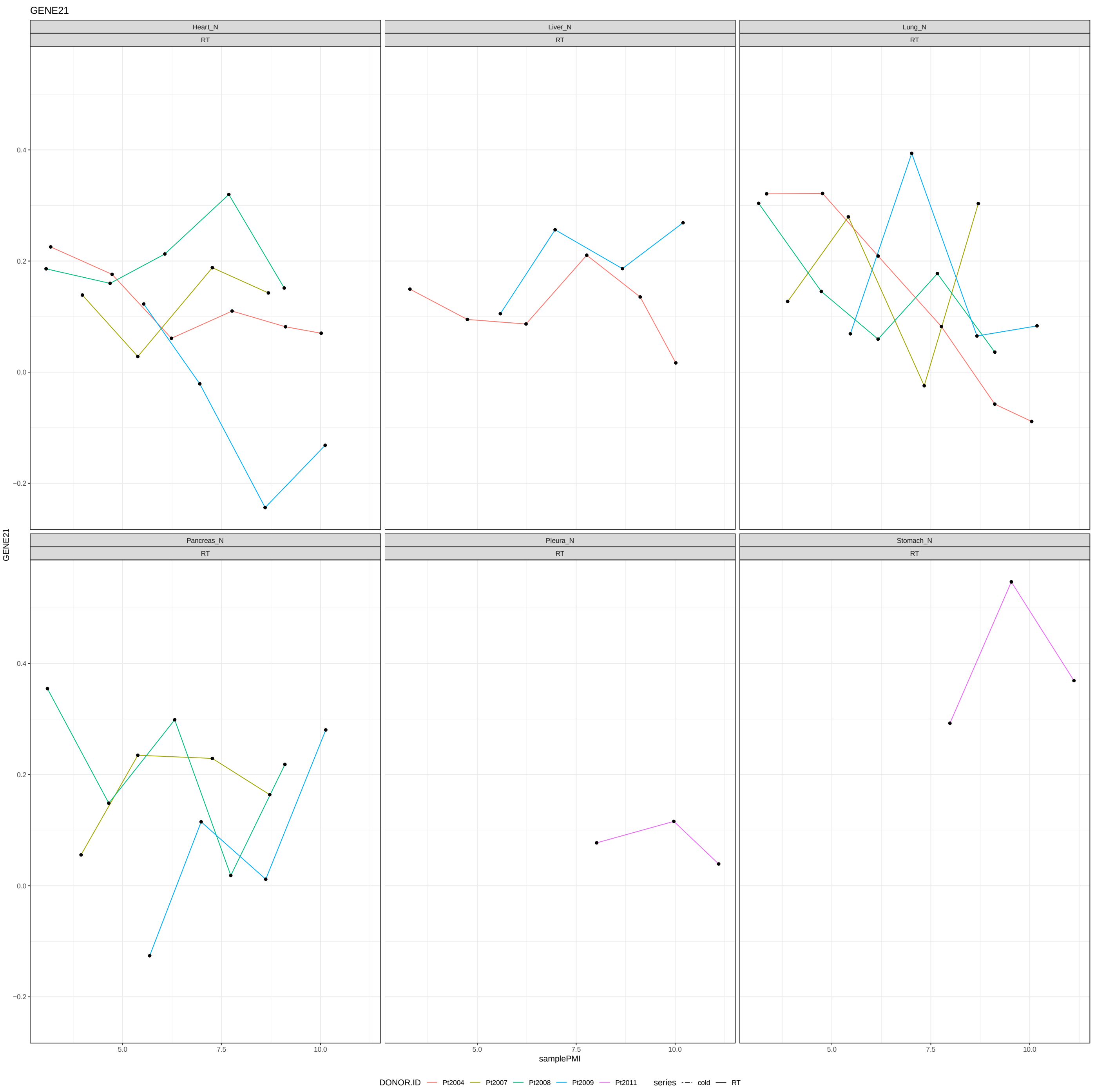

GENE21
Heart_N
RT
Liver_N
RT
Lung_N
RT
Pancreas_N
RT
Pleura_N
RT
Stomach_N
RT
GENE21
0.4
0.2
0.0
−0.2
5.0
7.5
10.0
samplePMI
DONOR.ID
Pt2004
Pt2007
Pt2008
Pt2009
Pt2011
series
cold
RT

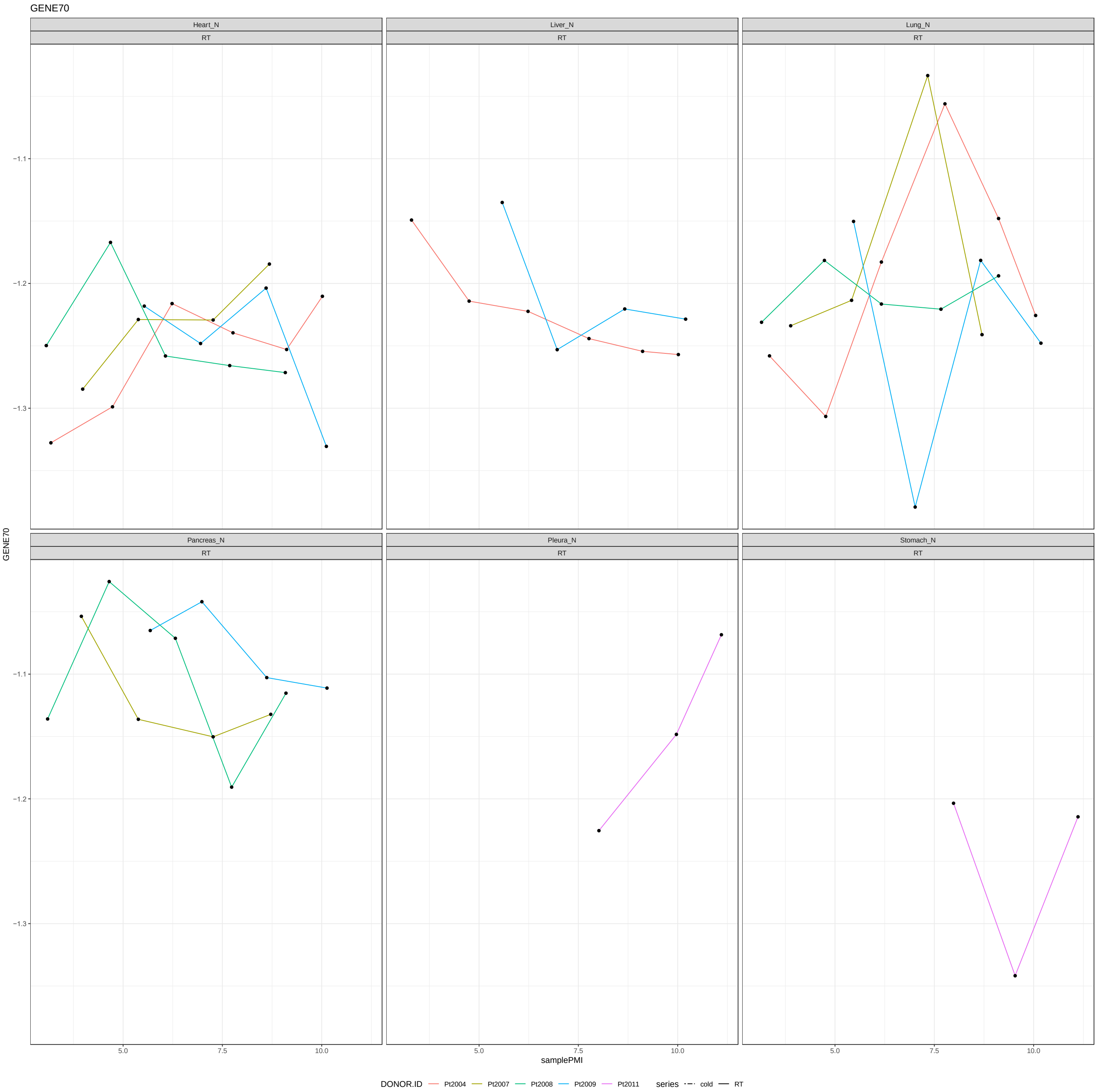
GENE70
Heart_N
RT
Liver_N
RT
Lung_N
RT
Pancreas_N
RT
Pleura_N
RT
Stomach_N
RT
−1.1
−1.2
−1.3
GENE70
5.0
7.5
10.0
samplePMI
DONOR.ID
Pt2004
Pt2007
Pt2008
Pt2009
Pt2011
series
cold
RT

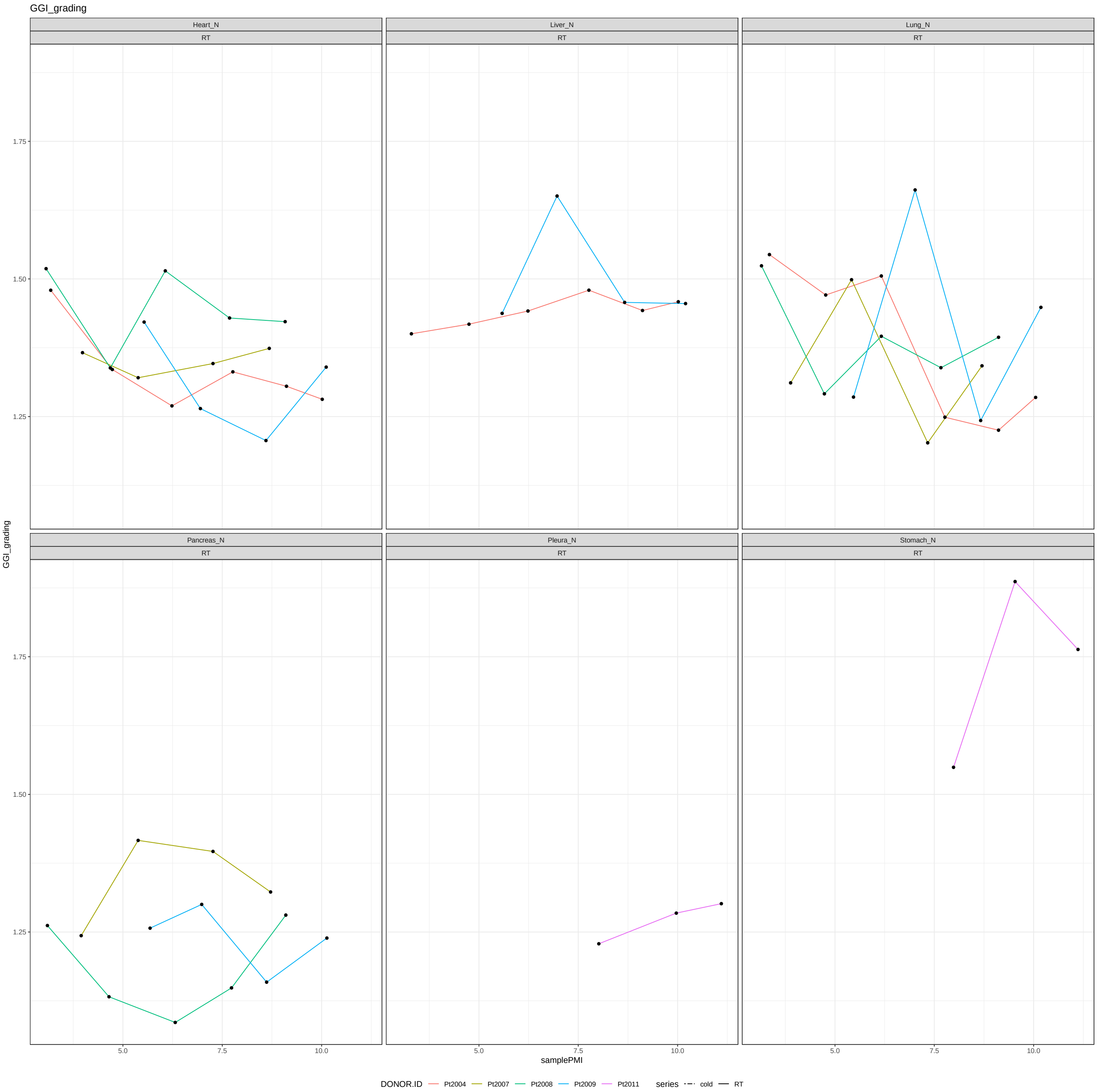

GGI_grading
Heart_N
RT
Liver_N
RT
Lung_N
RT
Pancreas_N
RT
Pleura_N
RT
Stomach_N
RT
1.75
1.50
1.25
GGI_grading
5.0
7.5
10.0
samplePMI
DONOR.ID
Pt2004
Pt2007
Pt2008
Pt2009
Pt2011
series
cold
RT

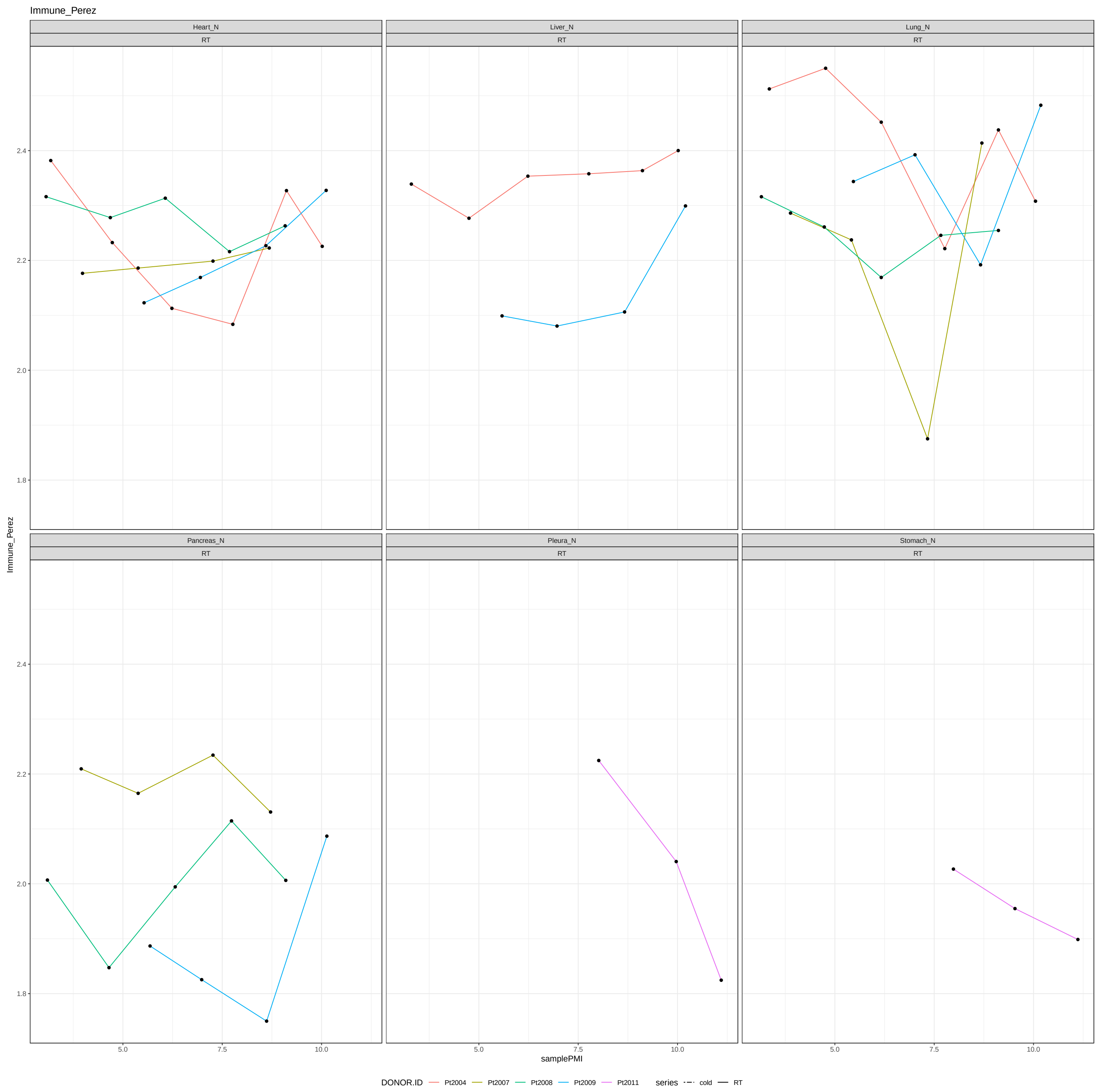
Immune_Perez
Heart_N
Liver_N
Lung_N
Pancreas_N
Pleura_N
Stomach_N
RT
Immune_Perez
samplePMI
2.4
2.2
2.0
1.8
5.0
7.5
10.0
DONOR.ID
Pt2004
Pt2007
Pt2008
Pt2009
Pt2011
series
cold
RT

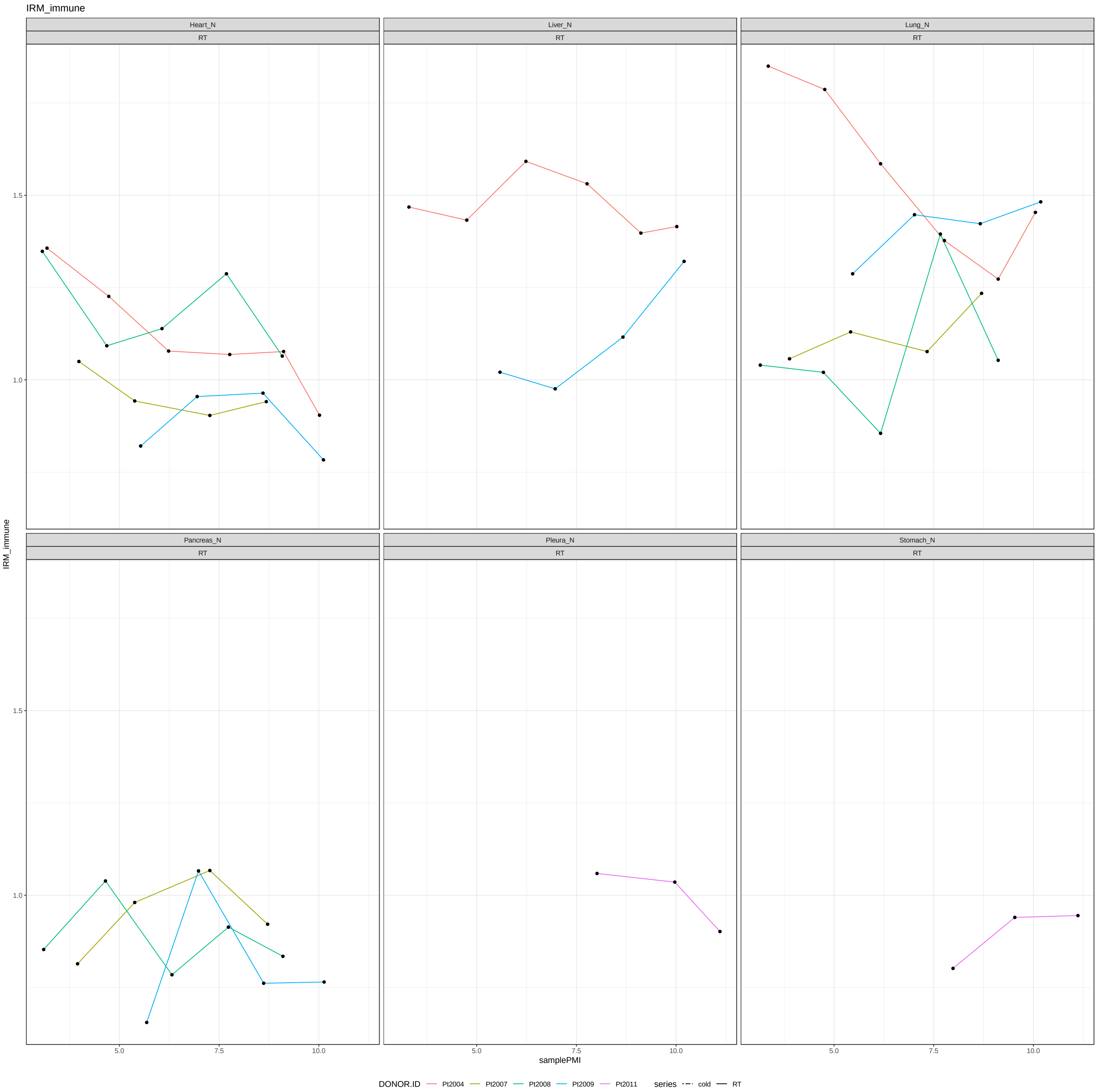
IRM_immune
Heart_N
Liver_N
Lung_N
Pancreas_N
Pleura_N
Stomach_N
RT
1.5
1.0
5.0
7.5
10.0
IRM_immune
samplePMI
DONOR.ID
Pt2004
Pt2007
Pt2008
Pt2009
Pt2011
series
cold
RT

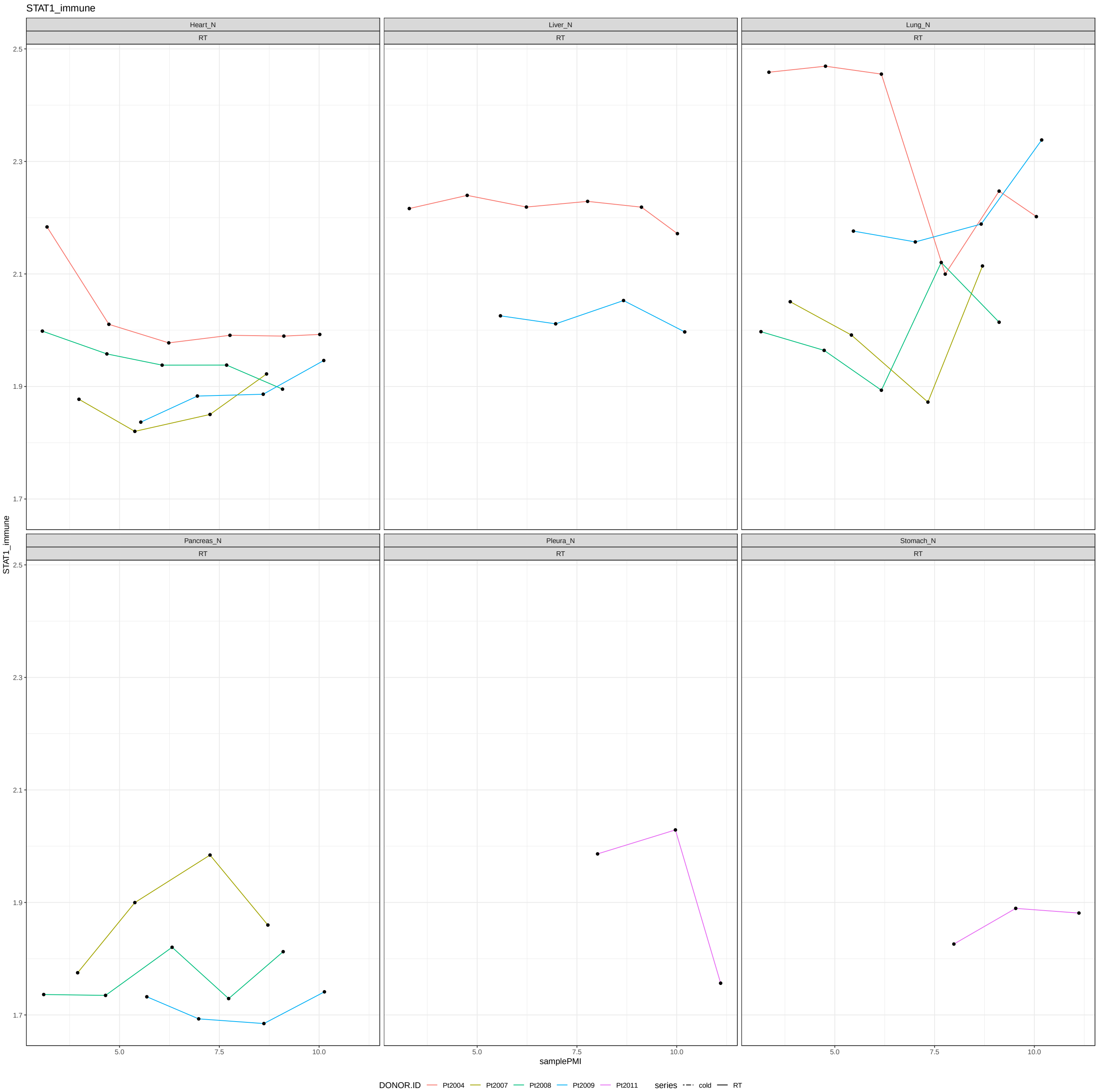
STAT1_immune
Heart_N
Liver_N
Lung_N
Pancreas_N
Pleura_N
Stomach_N
RT
2.5
2.3
2.1
1.9
1.7
STAT1_immune
5.0
7.5
10.0
samplePMI
DONOR.ID
Pt2004
Pt2007
Pt2008
Pt2009
Pt2011
series
cold
RT

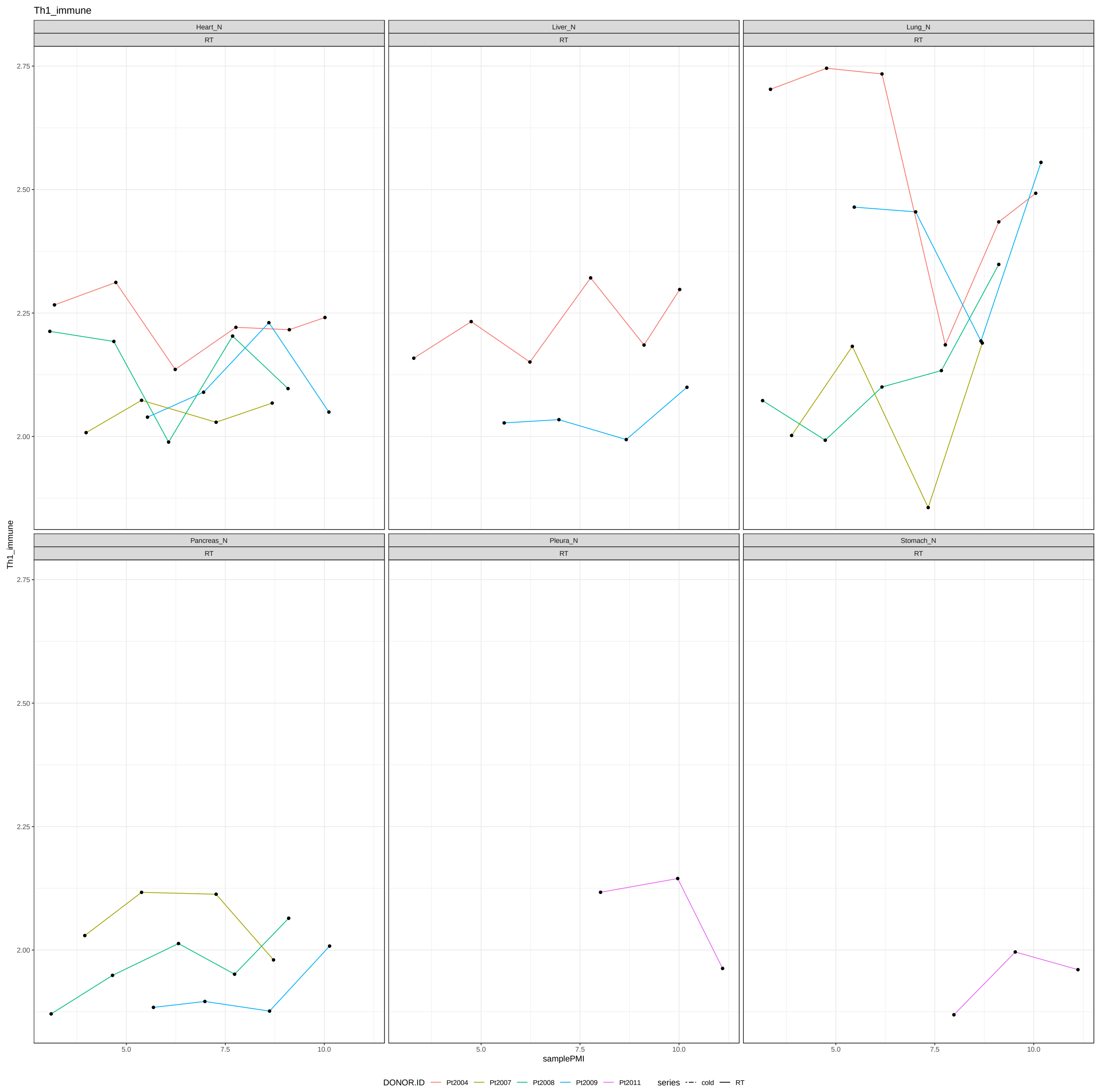

Th1_immune
Heart_N
RT
Liver_N
RT
Lung_N
RT
Pancreas_N
RT
Pleura_N
RT
Stomach_N
RT
Th1_immune
2.75
2.50
2.25
2.00
5.0
7.5
10.0
samplePMI
DONOR.ID
Pt2004
Pt2007
Pt2008
Pt2009
Pt2011
series
cold
RT

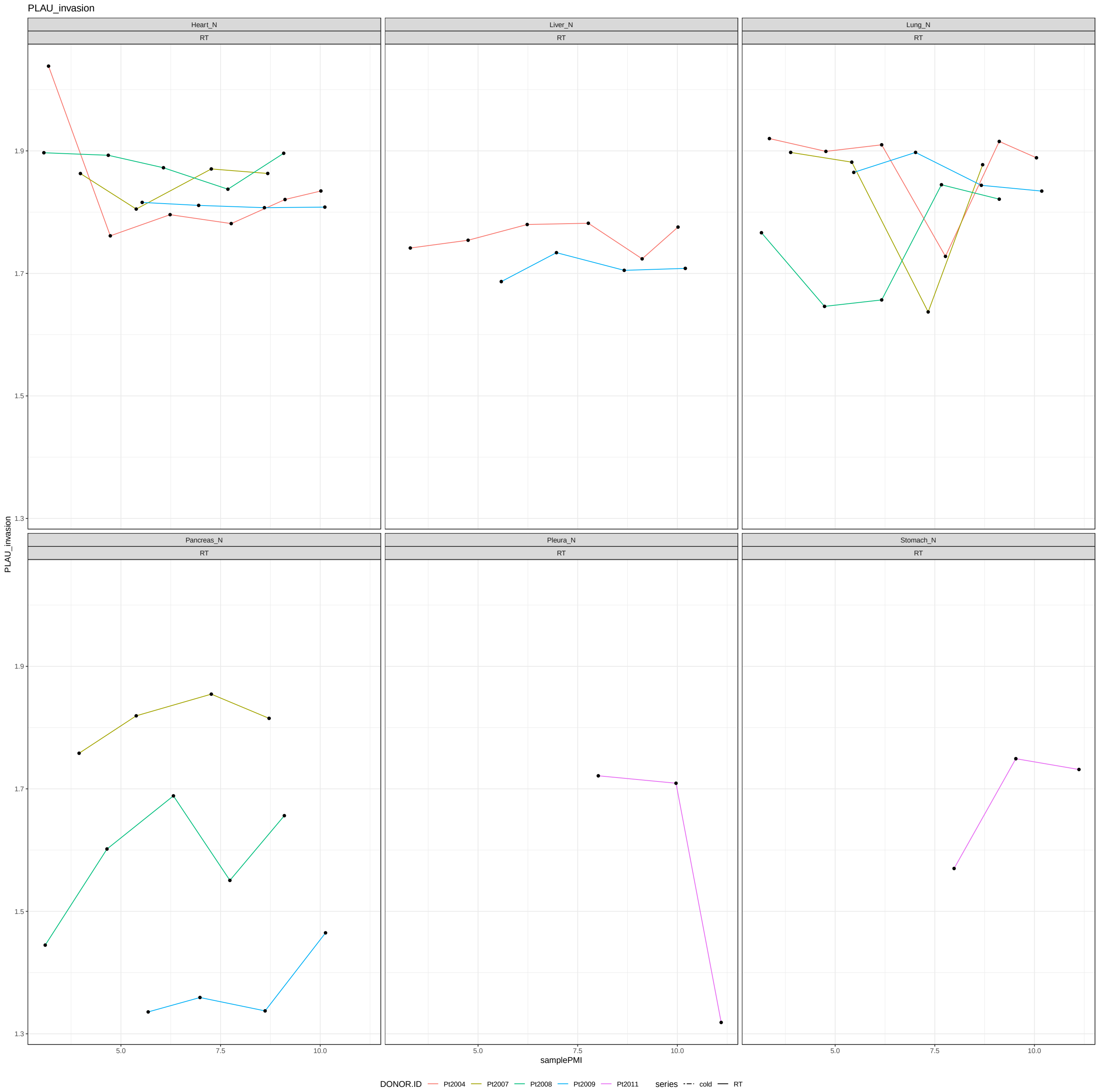
PLAU_invasion
Heart_N
RT
Liver_N
RT
Lung_N
RT
Pancreas_N
RT
Pleura_N
RT
Stomach_N
RT
1.9
1.7
1.5
1.3
5.0
7.5
10.0
PLAU_invasion
samplePMI
DONOR.ID
Pt2004
Pt2007
Pt2008
Pt2009
Pt2011
series
cold
RT

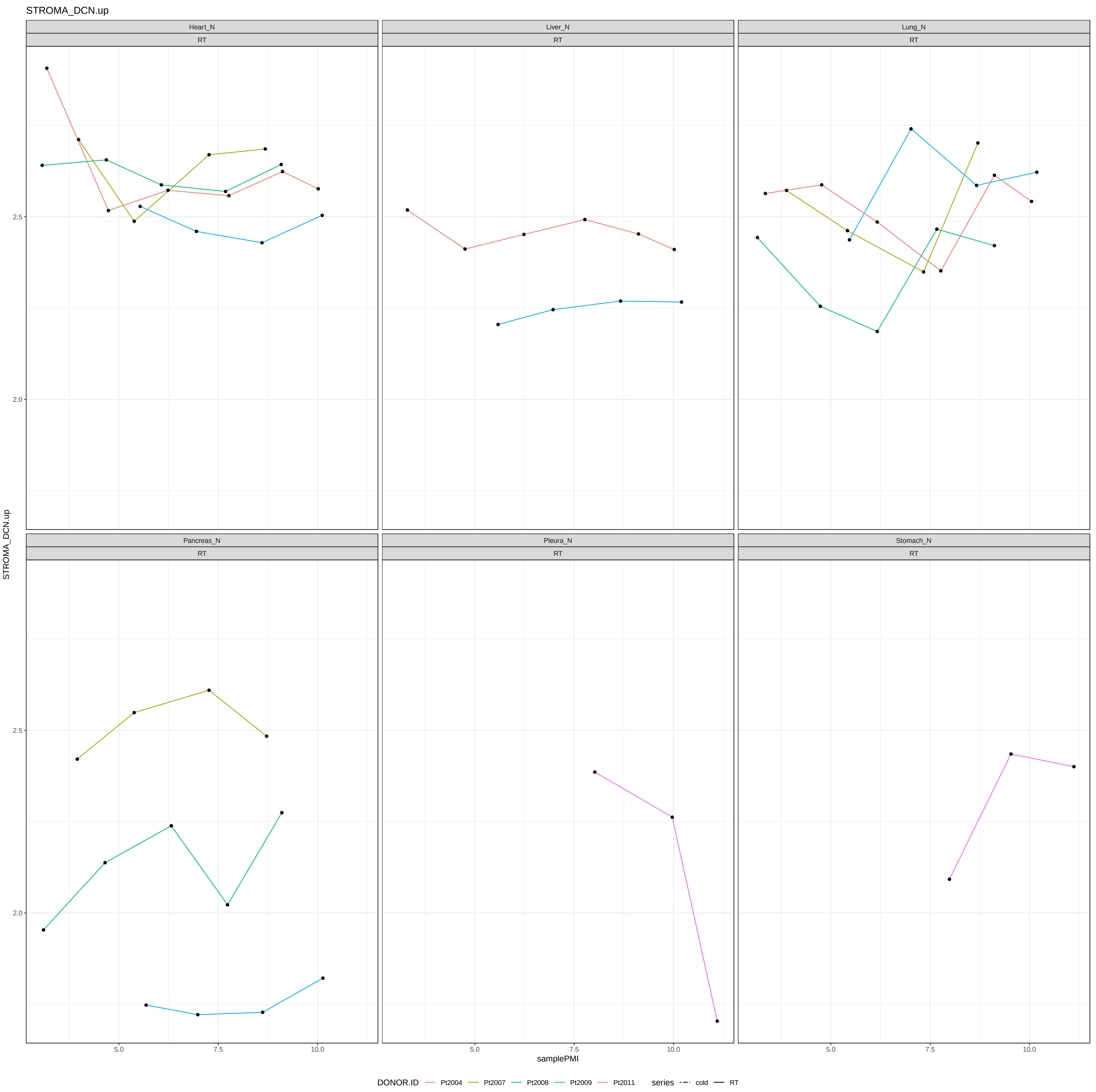
STROMA_DCN.up
Heart_N
Liver_N
Lung_N
Pancreas_N
Pleura_N
Stomach_N
RT
2.5
2.0
5.0
7.5
10.0
STROMA_DCN.up
samplePMI
DONOR.ID
Pt2004
Pt2007
Pt2008
Pt2009
Pt2011
series
cold
RT

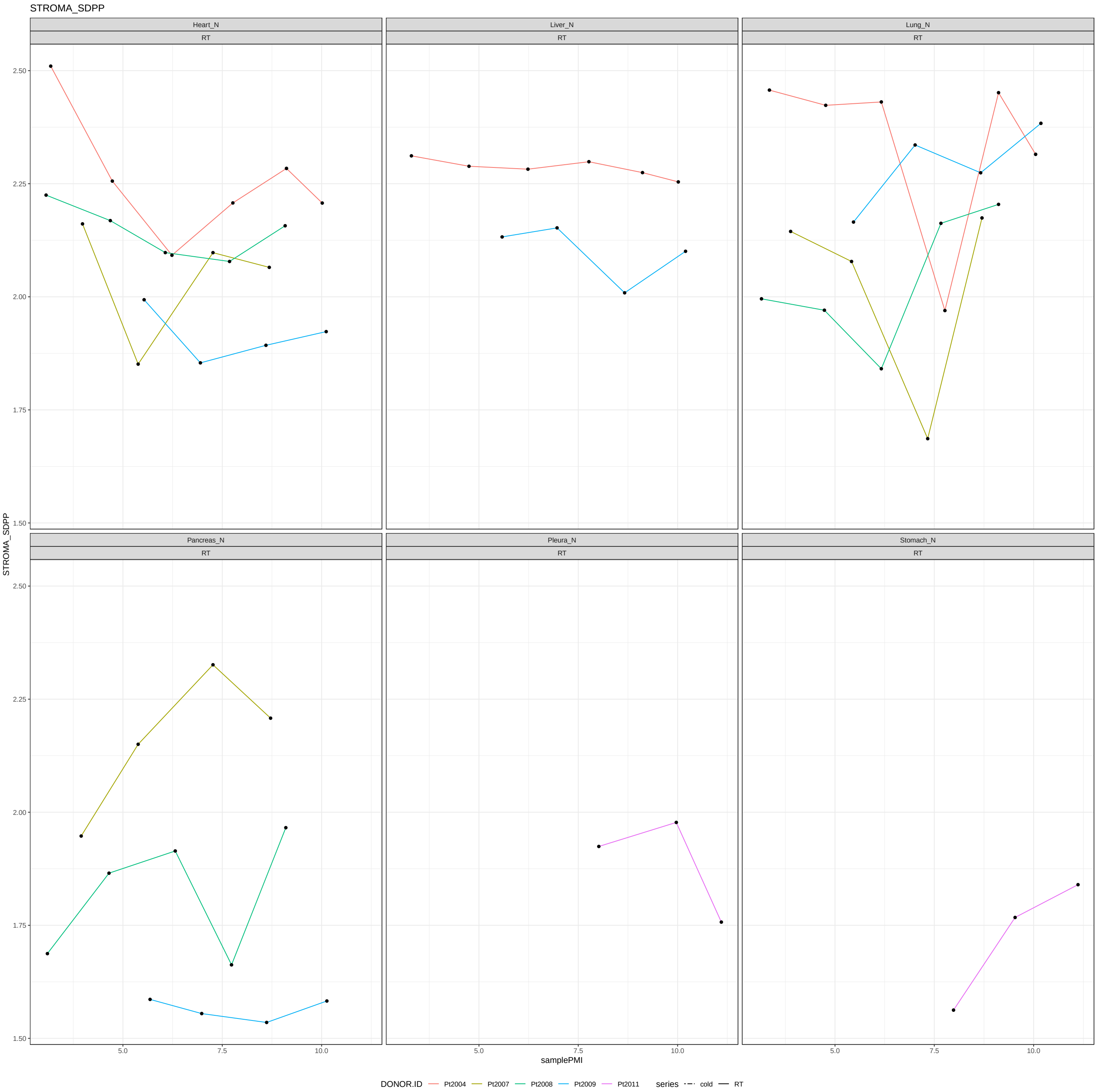

STROMA_SDPP
Heart_N
Liver_N
Lung_N
Pancreas_N
Pleura_N
Stomach_N
RT
2.50
2.25
2.00
1.75
1.50
5.0
7.5
10.0
STROMA_SDPP
samplePMI
DONOR.ID
Pt2004
Pt2007
Pt2008
Pt2009
Pt2011
series
cold
RT

# Supplementary Figure 3

Trajectories of sequencing metrics and transcriptomics signatures in tumor tissues.

Abbreviations: AR: number of assigned reads to genes, genecount1: number of expressed genes, N: non-tumor tissue, P: tumor tissue, cold: samples cooled between 4°C and 10°C, RT: room temperature, samplePMI: sample specific post-mortem interval.

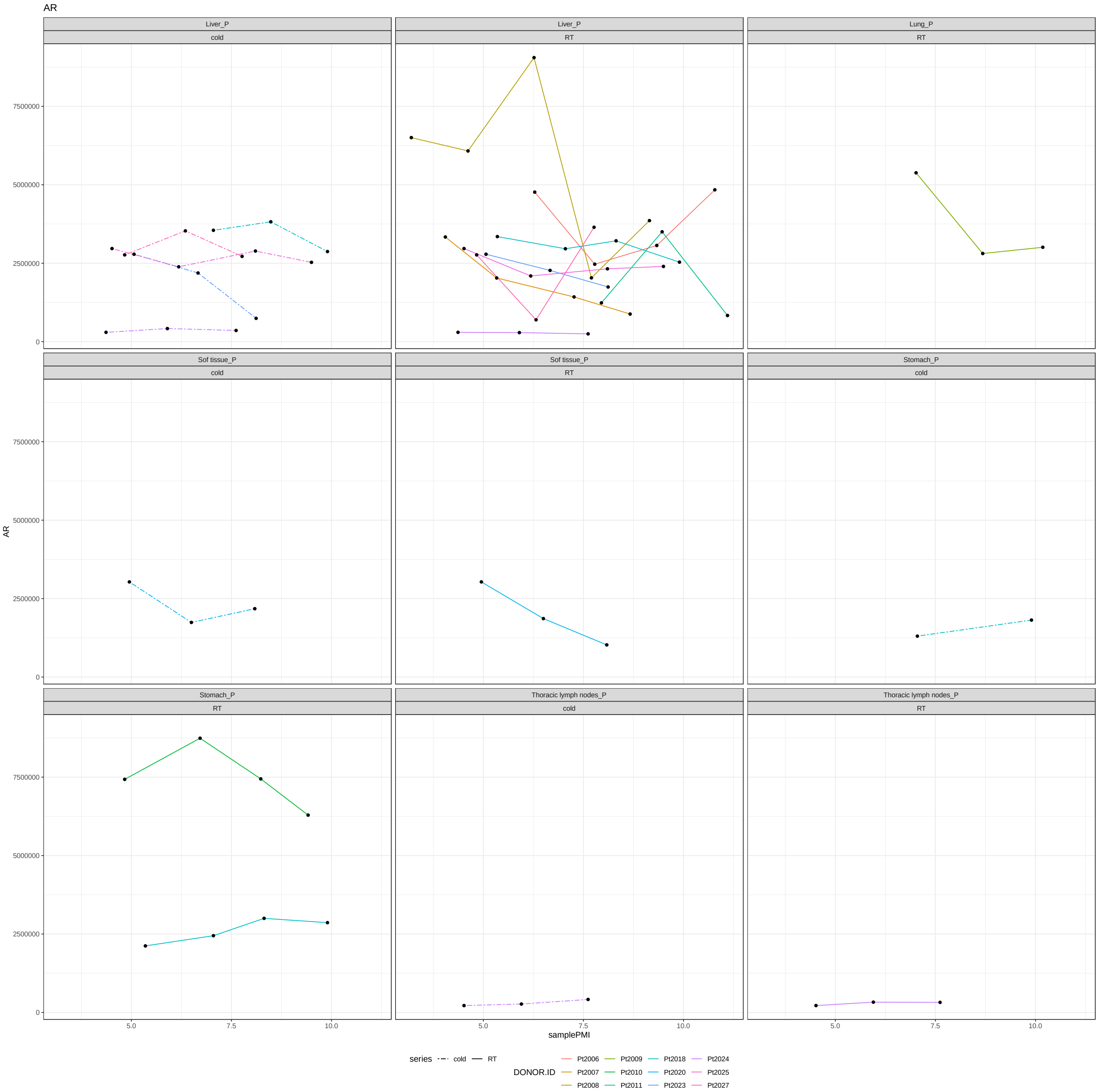

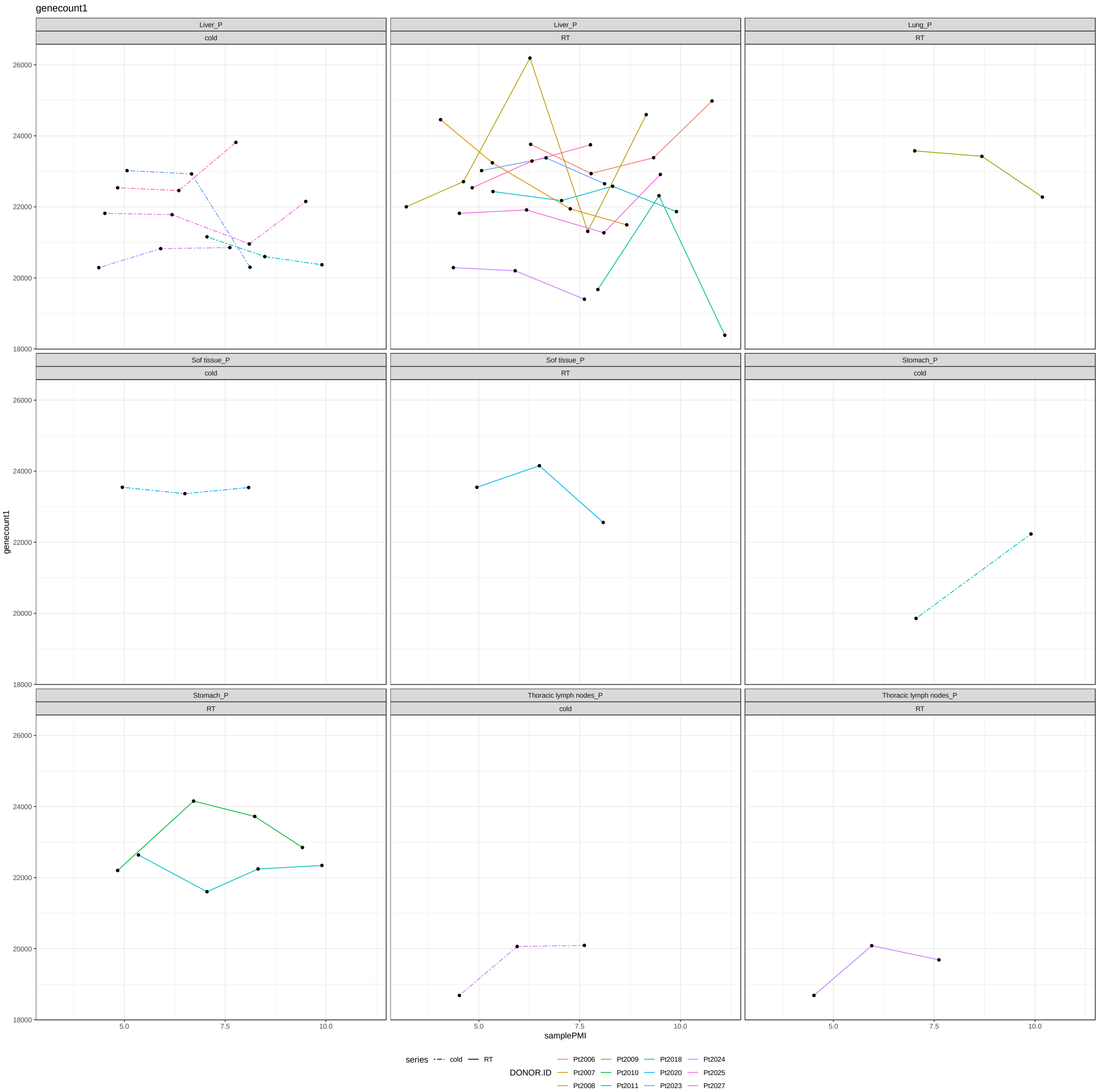
genecount1
Liver_P
cold
Liver_P
RT
Lung_P
RT
Sof tissue_P
cold
Sof tissue_P
RT
Stomach_P
cold
Stomach_P
RT
Thoracic lymph nodes_P
cold
Thoracic lymph nodes_P
RT
genecount1
26000
24000
22000
20000
18000
5.0
7.5
10.0
samplePMI
series
cold
RT
DONOR.ID
Pt2006
Pt2007
Pt2008
Pt2009
Pt2010
Pt2011
Pt2018
Pt2020
Pt2023
Pt2024
Pt2025
Pt2027

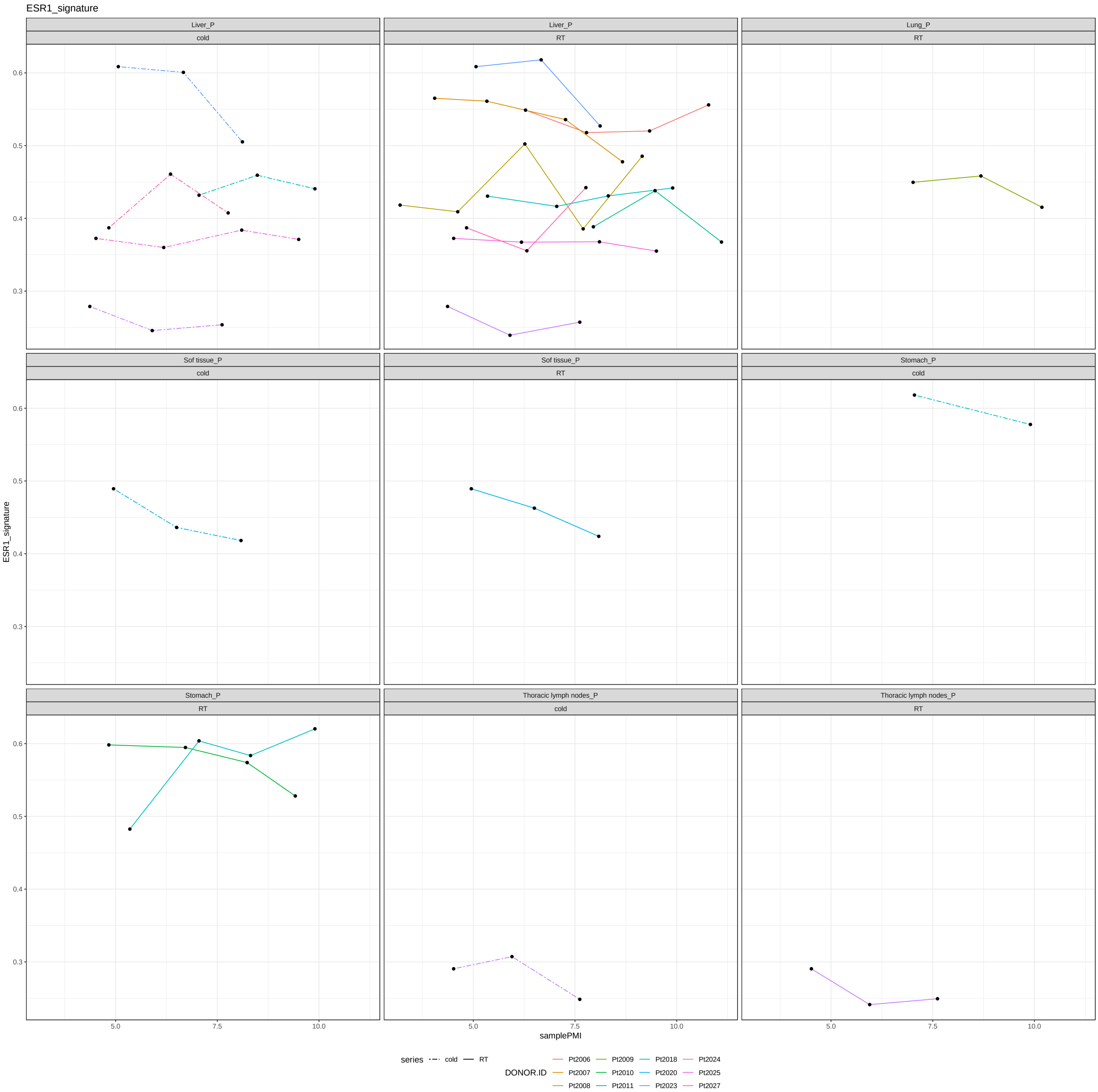
ESR1_signature
Liver_P
cold
Liver_P
RT
Lung_P
RT
Sof tissue_P
cold
Sof tissue_P
RT
Stomach_P
cold
Stomach_P
RT
Thoracic lymph nodes_P
cold
Thoracic lymph nodes_P
RT
ESR1_signature
0.6
0.5
0.4
0.3
5.0
7.5
10.0
samplePMI
series cold RT
DONOR.ID
Pt2006 Pt2009 Pt2018 Pt2024
Pt2007 Pt2010 Pt2020 Pt2025
Pt2008 Pt2011 Pt2023 Pt2027

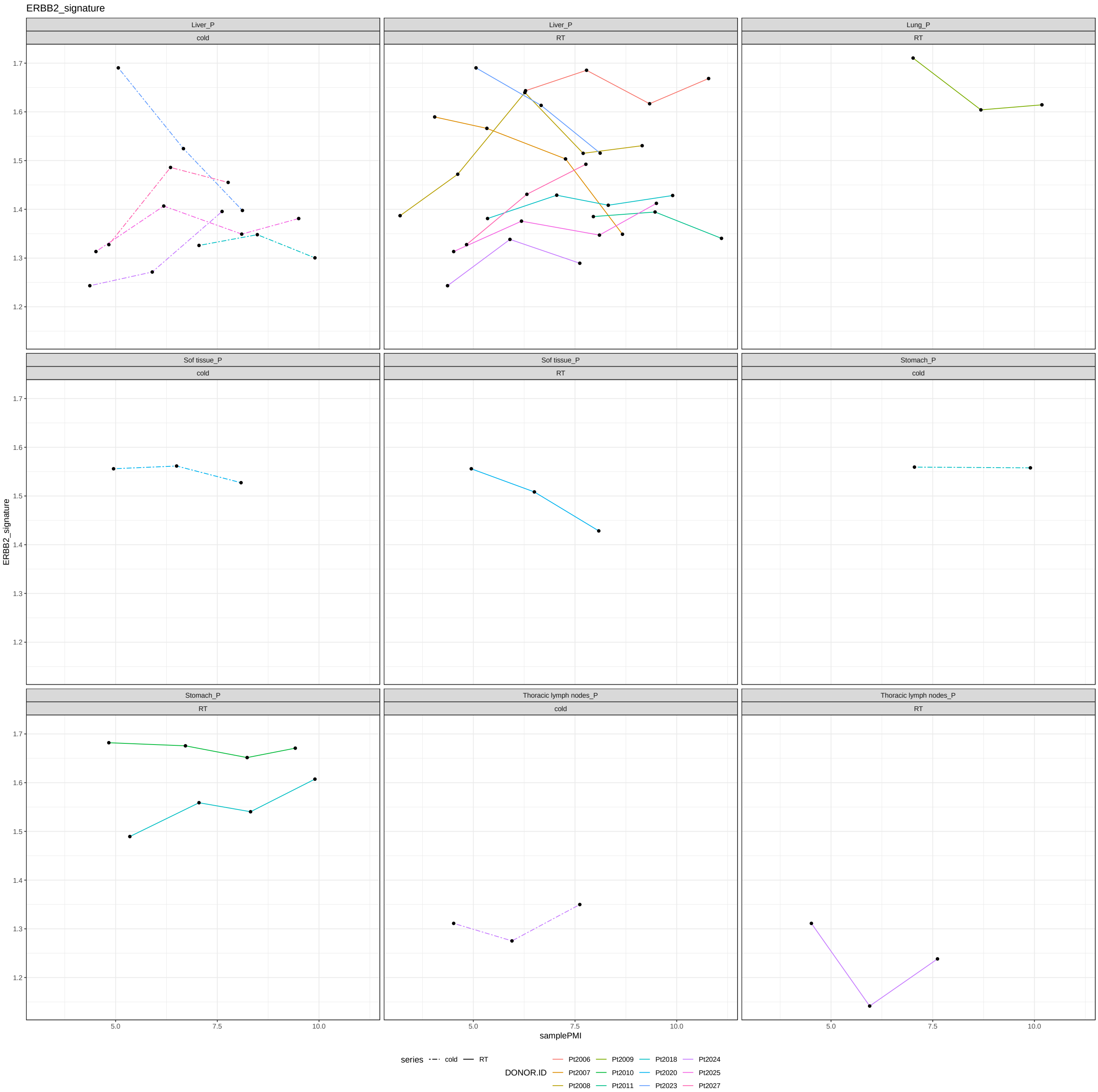
ERBB2_signature
Liver_P
cold
RT
Lung_P
Sof tissue_P
Stomach_P
Thoracic lymph nodes_P
ERBB2_signature
samplePMI
1.7
1.6
1.5
1.4
1.3
1.2
5.0
7.5
10.0
series
cold
RT
DONOR.ID
Pt2006
Pt2007
Pt2008
Pt2009
Pt2010
Pt2011
Pt2018
Pt2020
Pt2023
Pt2024
Pt2025
Pt2027

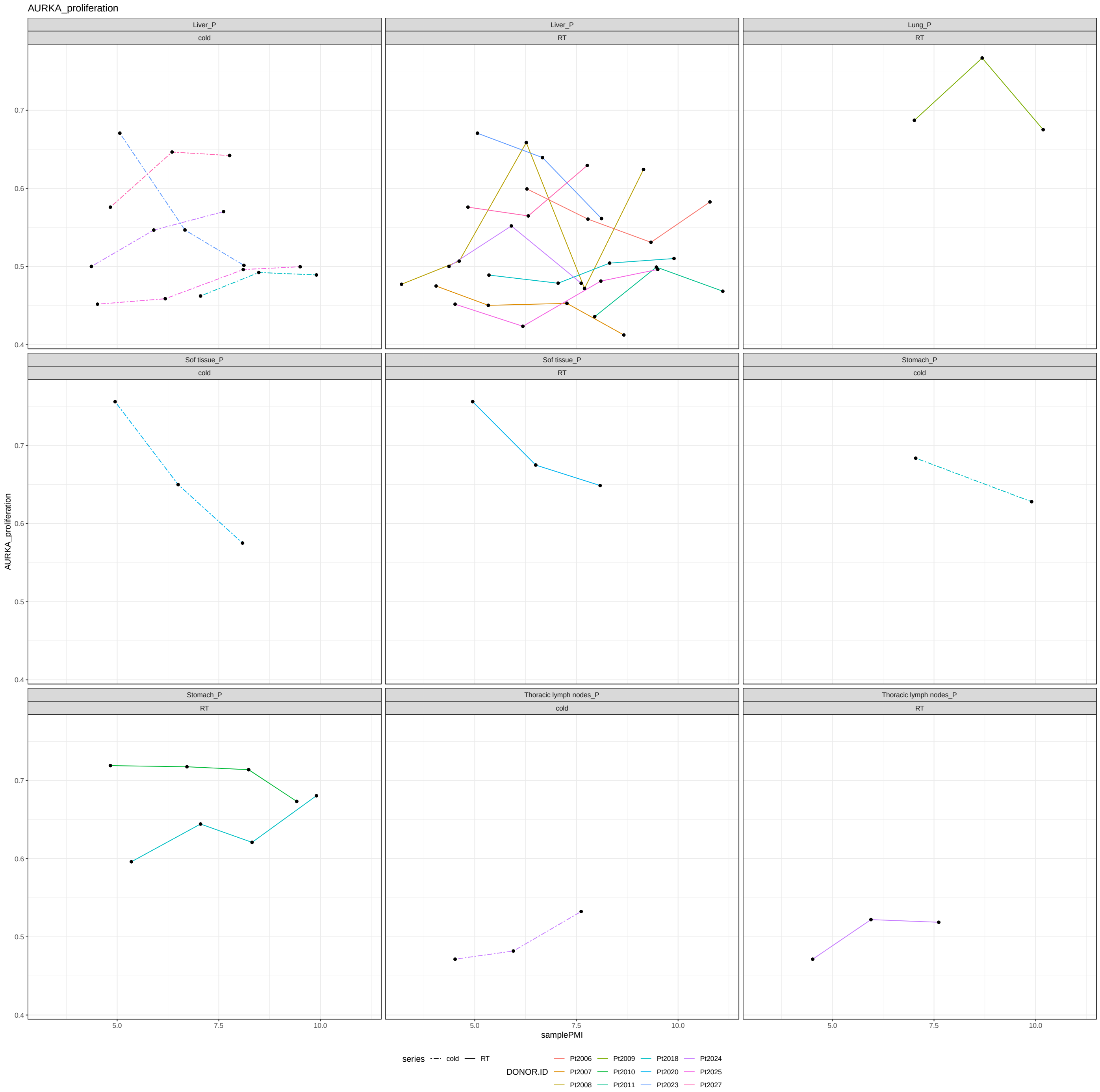

AURKA_proliferation
Liver_P
cold
Liver_P
RT
Lung_P
RT
Sof tissue_P
cold
Sof tissue_P
RT
Stomach_P
cold
Stomach_P
RT
Thoracic lymph nodes_P
cold
Thoracic lymph nodes_P
RT
AURKA_proliferation
0.4
0.5
0.6
0.7
5.0
7.5
10.0
samplePMI
series
cold
RT
DONOR.ID
Pt2006
Pt2007
Pt2008
Pt2009
Pt2010
Pt2011
Pt2018
Pt2020
Pt2023
Pt2024
Pt2025
Pt2027

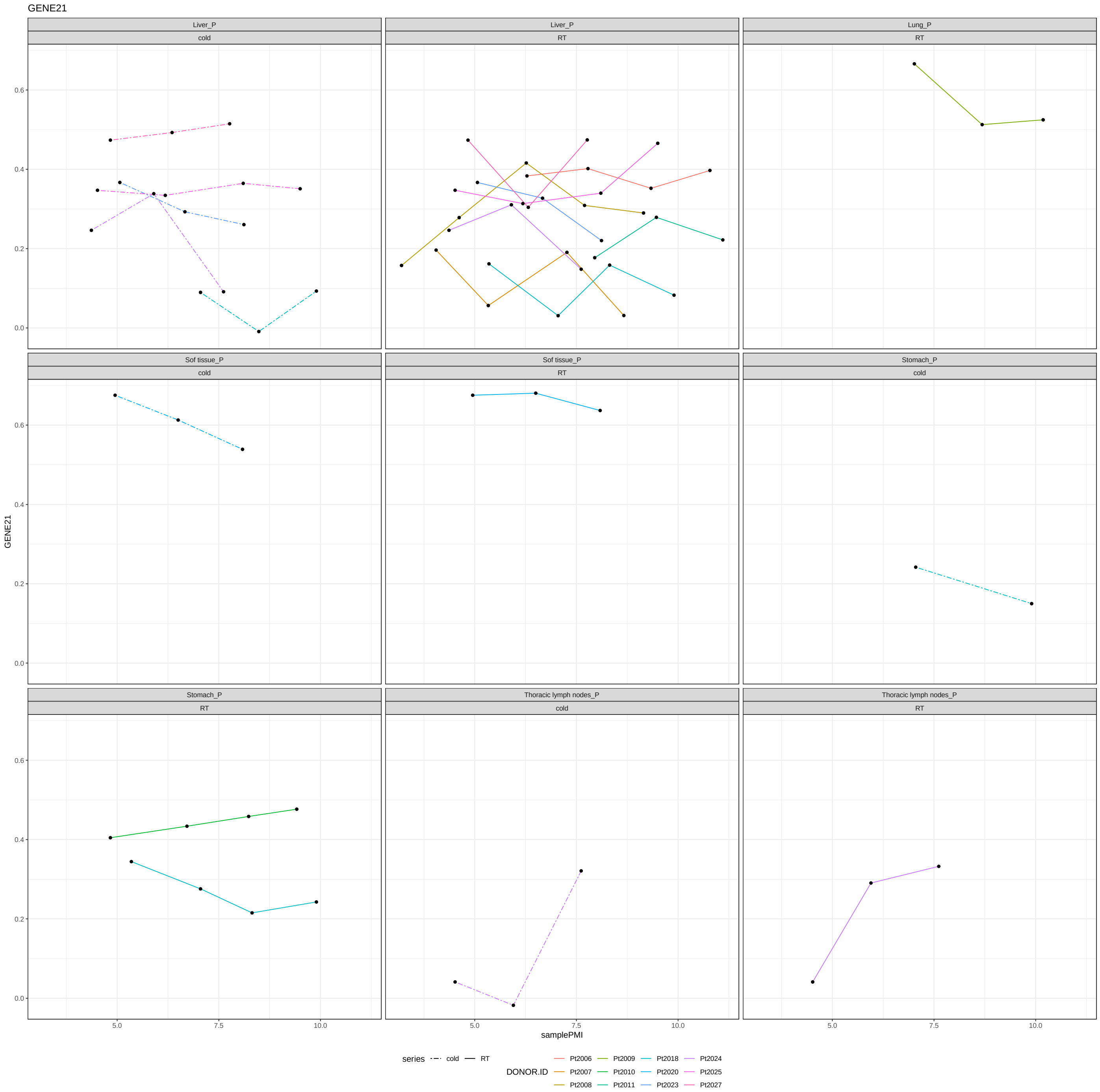

GENE21
Liver_P
cold
Liver_P
RT
Lung_P
RT
Sof tissue_P
cold
Sof tissue_P
RT
Stomach_P
cold
Stomach_P
RT
Thoracic lymph nodes_P
cold
Thoracic lymph nodes_P
RT
0.0
0.2
0.4
0.6
GENE21
5.0
7.5
10.0
samplePMI
series cold RT
DONOR.ID
Pt2006 Pt2009 Pt2018 Pt2024
Pt2007 Pt2010 Pt2020 Pt2025
Pt2008 Pt2011 Pt2023 Pt2027

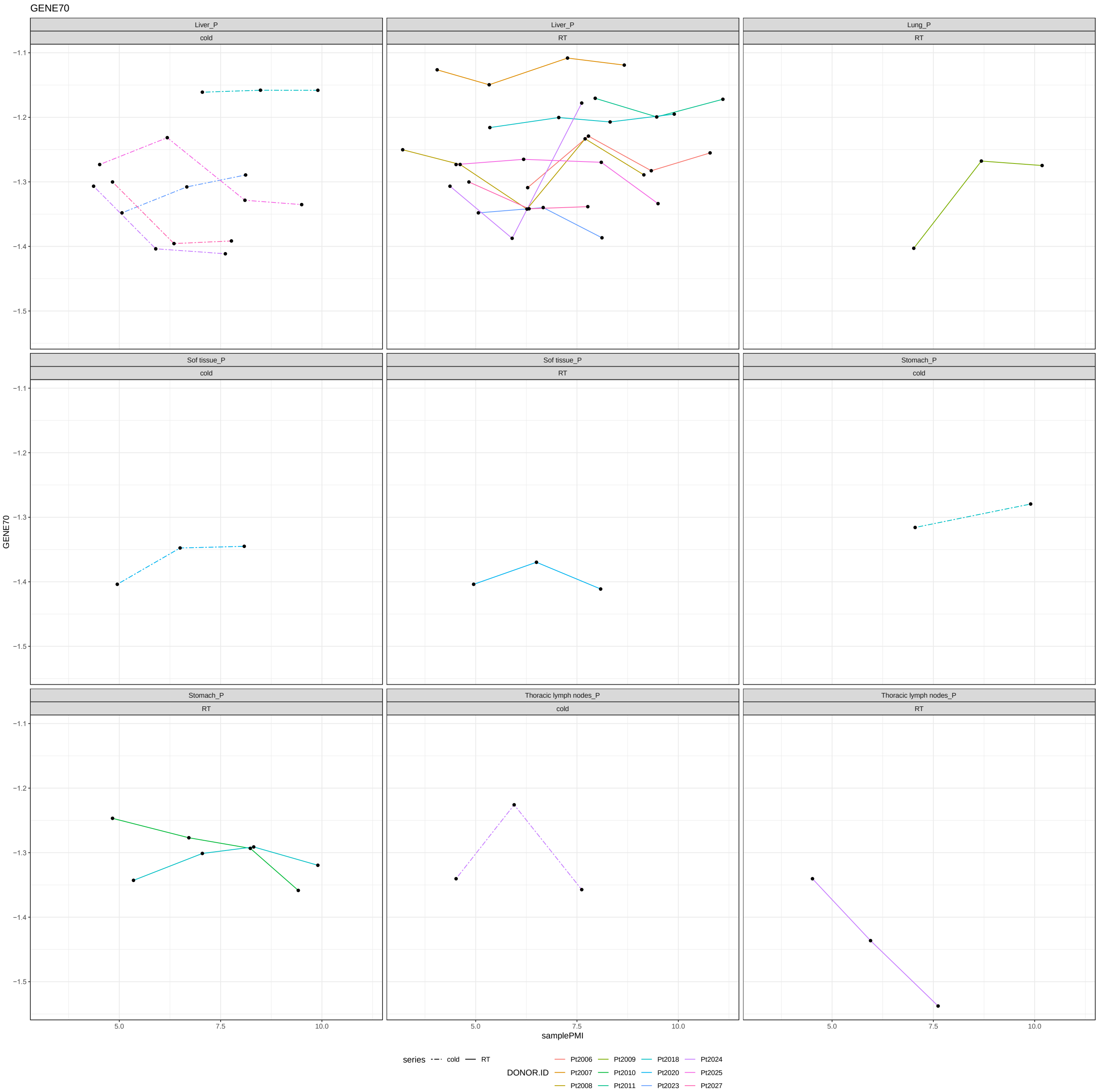
GENE70
Liver_P
cold
Liver_P
RT
Lung_P
RT
Sof tissue_P
cold
Sof tissue_P
RT
Stomach_P
cold
Stomach_P
RT
Thoracic lymph nodes_P
cold
Thoracic lymph nodes_P
RT
−1.1
−1.2
−1.3
−1.4
−1.5
GENE70
5.0
7.5
10.0
samplePMI
series
cold
RT
DONOR.ID
Pt2006
Pt2007
Pt2008
Pt2009
Pt2010
Pt2011
Pt2018
Pt2020
Pt2023
Pt2024
Pt2025
Pt2027

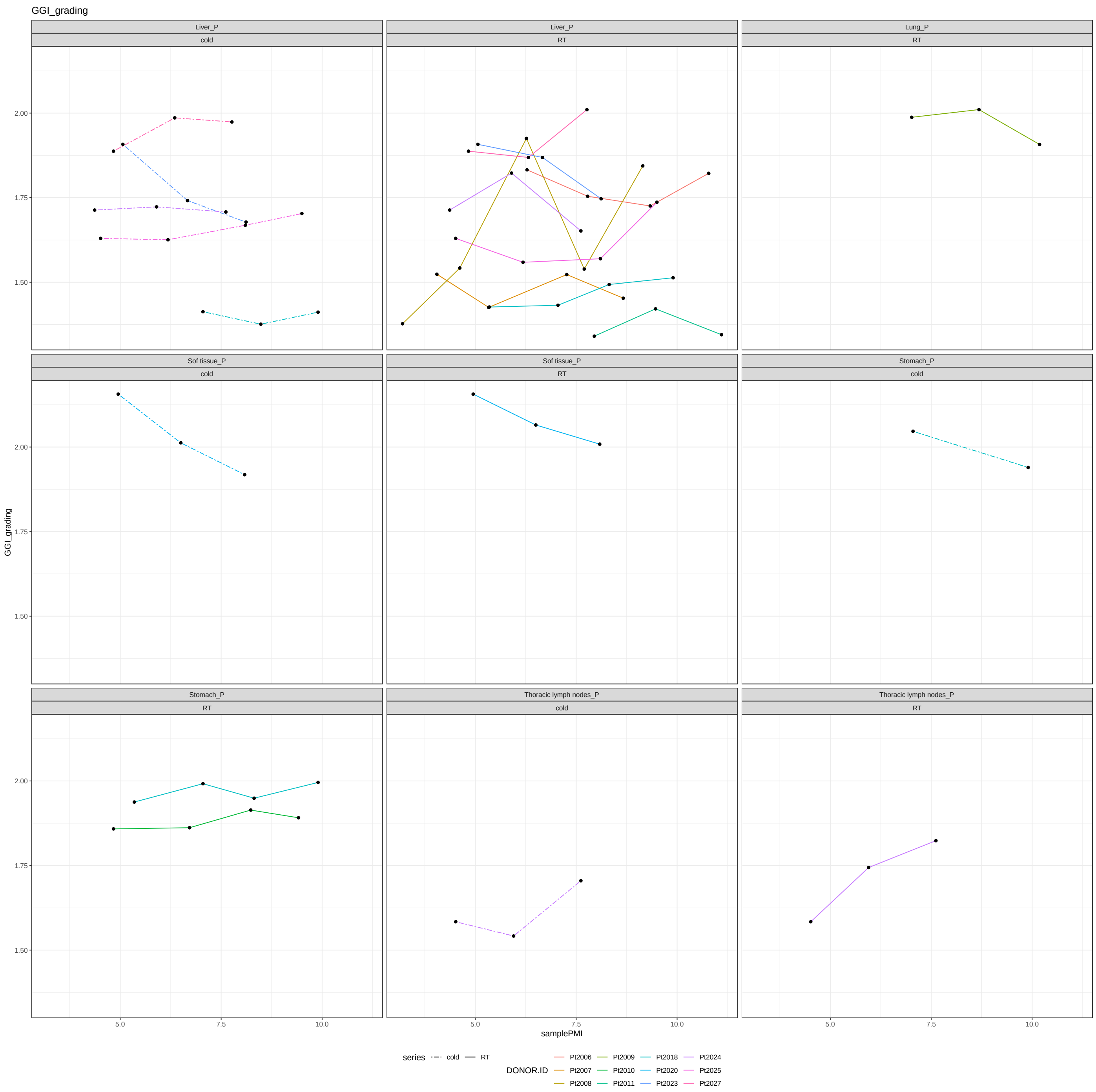
GGI_grading
Liver_P
cold
Liver_P
RT
Lung_P
RT
Sof tissue_P
cold
Sof tissue_P
RT
Stomach_P
cold
Stomach_P
RT
Thoracic lymph nodes_P
cold
Thoracic lymph nodes_P
RT
GGI_grading
2.00
1.75
1.50
5.0
7.5
10.0
samplePMI
series
cold
RT
DONOR.ID
Pt2006
Pt2007
Pt2008
Pt2009
Pt2010
Pt2011
Pt2018
Pt2020
Pt2023
Pt2024
Pt2025
Pt2027

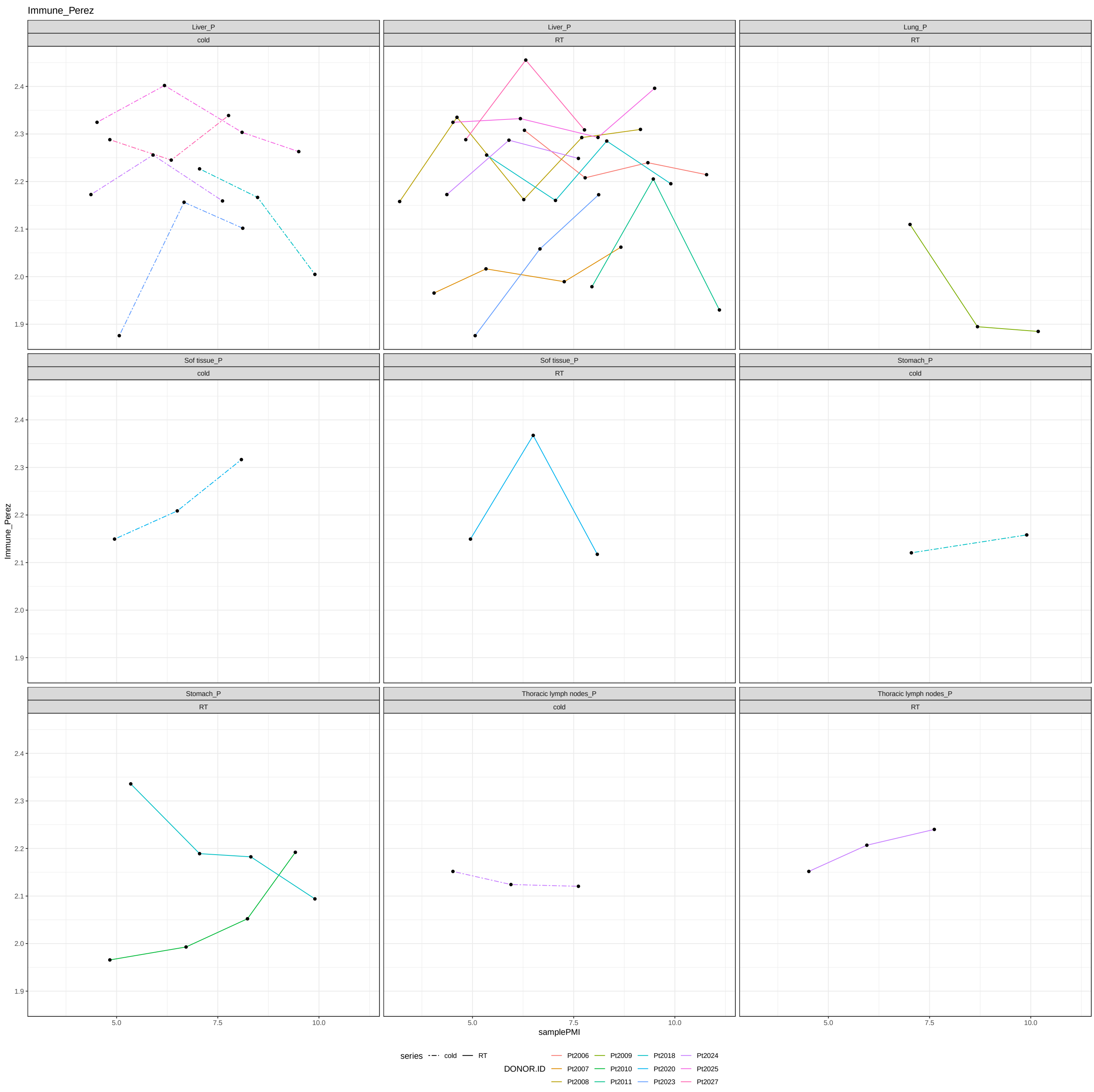

Immune_Perez
Liver_P
cold
Liver_P
RT
Lung_P
RT
Sof tissue_P
cold
Sof tissue_P
RT
Stomach_P
cold
Stomach_P
RT
Thoracic lymph nodes_P
cold
Thoracic lymph nodes_P
RT
2.4
2.3
2.2
2.1
2.0
1.9
Immune_Perez
5.0
7.5
10.0
samplePMI
series
cold
RT
DONOR.ID
Pt2006
Pt2007
Pt2008
Pt2009
Pt2010
Pt2011
Pt2018
Pt2020
Pt2023
Pt2024
Pt2025
Pt2027

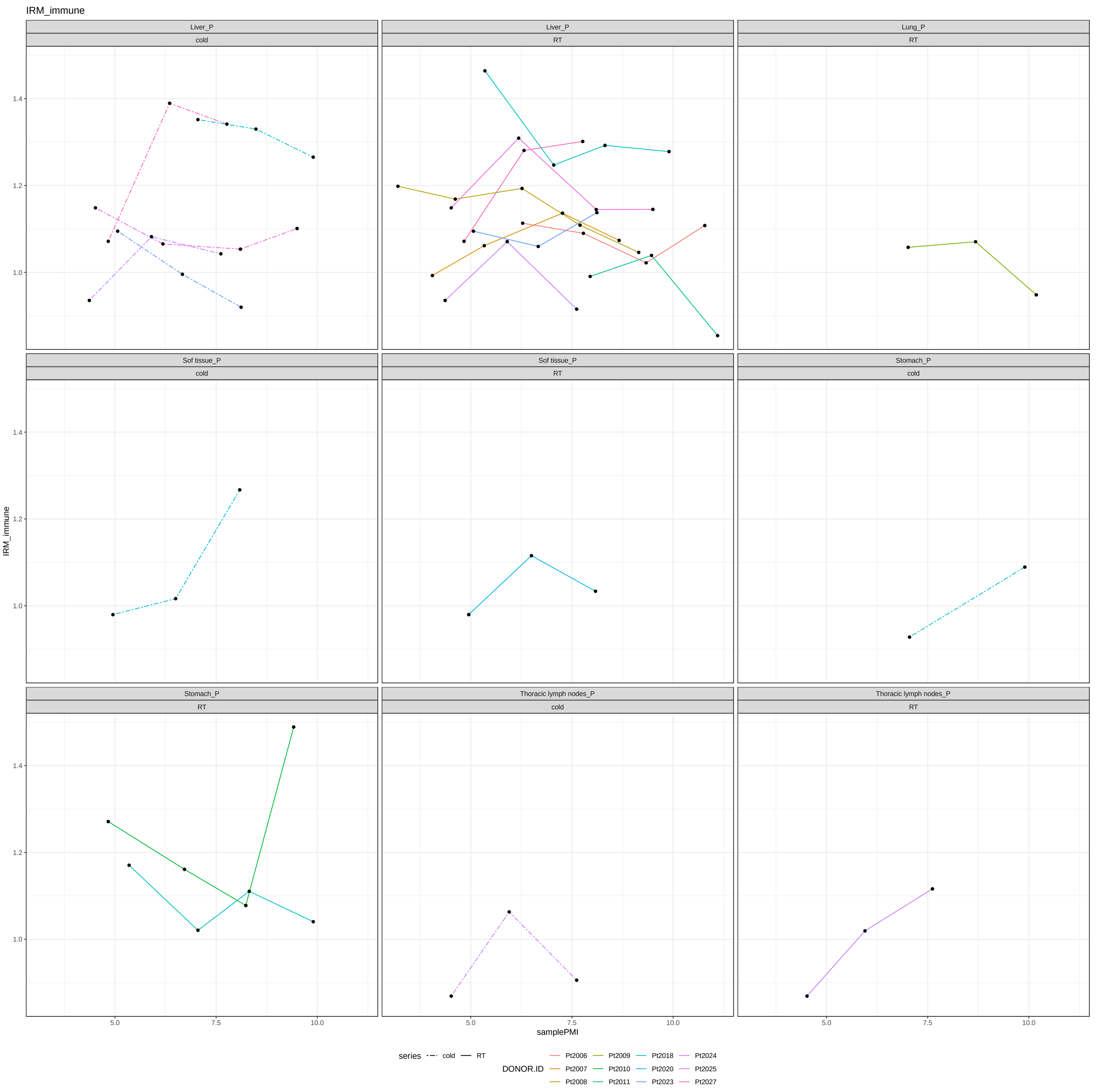
IRM_immune
Liver_P
cold
Liver_P
RT
Lung_P
RT
Sof tissue_P
cold
Sof tissue_P
RT
Stomach_P
cold
Stomach_P
RT
Thoracic lymph nodes_P
cold
Thoracic lymph nodes_P
RT
1.4
1.2
1.0
IRM_immune
5.0
7.5
10.0
samplePMI
series
cold
RT
DONOR.ID
Pt2006
Pt2007
Pt2008
Pt2009
Pt2010
Pt2011
Pt2018
Pt2020
Pt2023
Pt2024
Pt2025
Pt2027

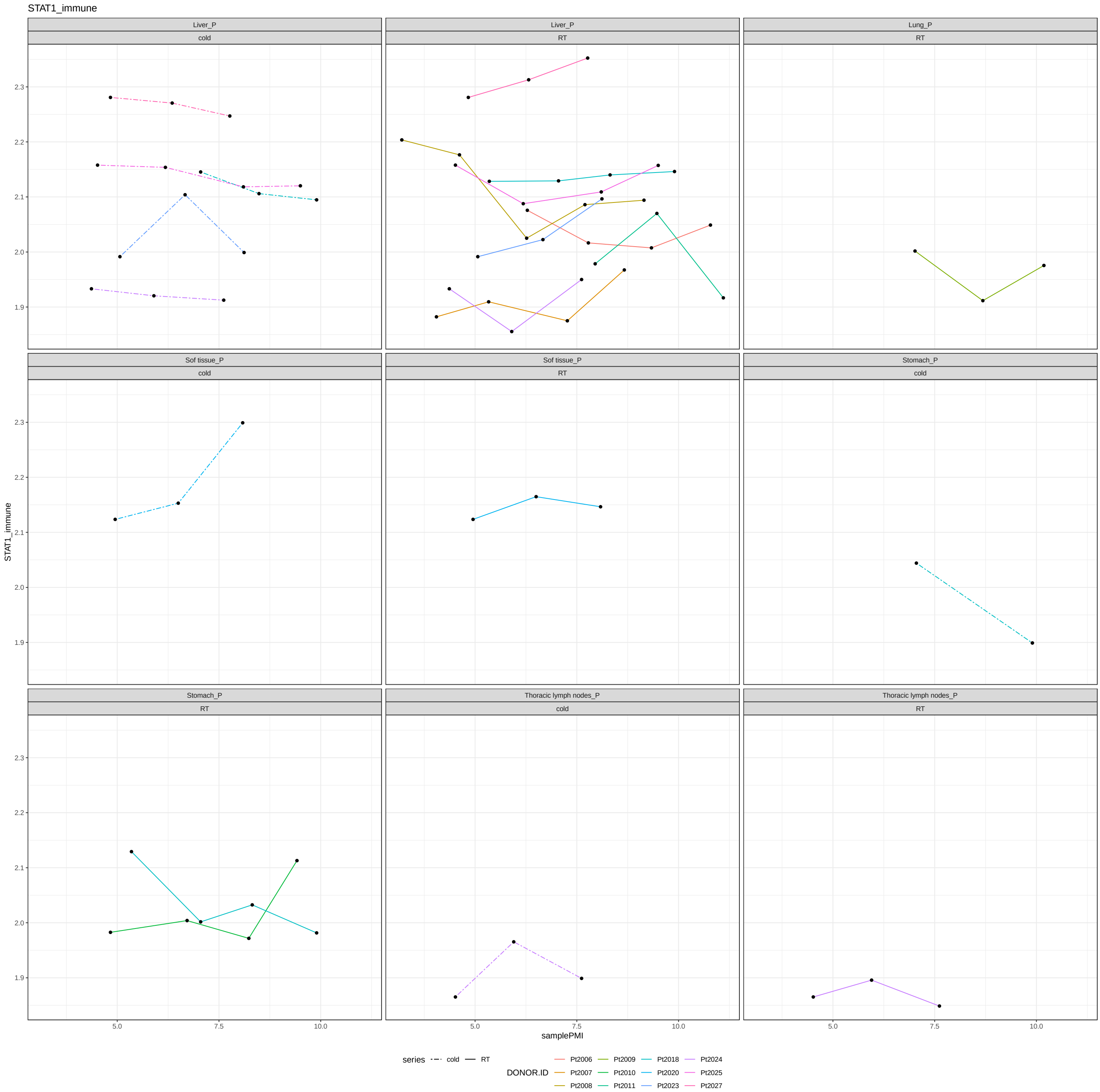
STAT1_immune
Liver_P
cold
Liver_P
RT
Lung_P
RT
Sof tissue_P
cold
Sof tissue_P
RT
Stomach_P
cold
Stomach_P
RT
Thoracic lymph nodes_P
cold
Thoracic lymph nodes_P
RT
STAT1_immune
samplePMI
series cold RT
DONOR.ID
Pt2006 Pt2007 Pt2008 Pt2009 Pt2010 Pt2011 Pt2018 Pt2020 Pt2023 Pt2024 Pt2025 Pt2027

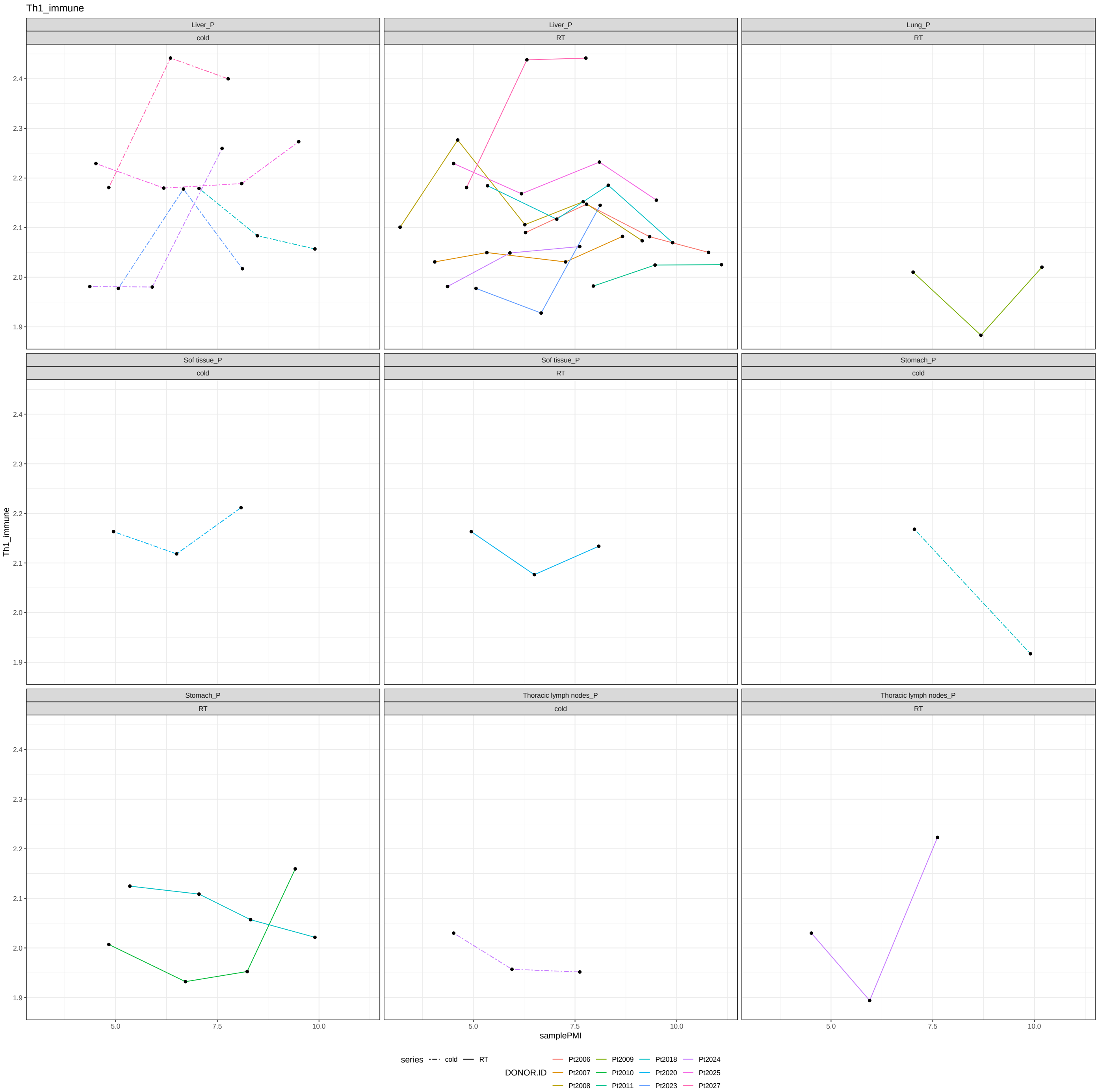
Th1_immune
Liver_P
cold
Liver_P
RT
Lung_P
RT
Sof tissue_P
cold
Sof tissue_P
RT
Stomach_P
cold
Stomach_P
RT
Thoracic lymph nodes_P
cold
Thoracic lymph nodes_P
RT
Th1_immune
2.4
2.3
2.2
2.1
2.0
1.9
5.0
7.5
10.0
samplePMI
series
cold
RT
DONOR.ID
Pt2006
Pt2007
Pt2008
Pt2009
Pt2010
Pt2011
Pt2018
Pt2020
Pt2023
Pt2024
Pt2025
Pt2027

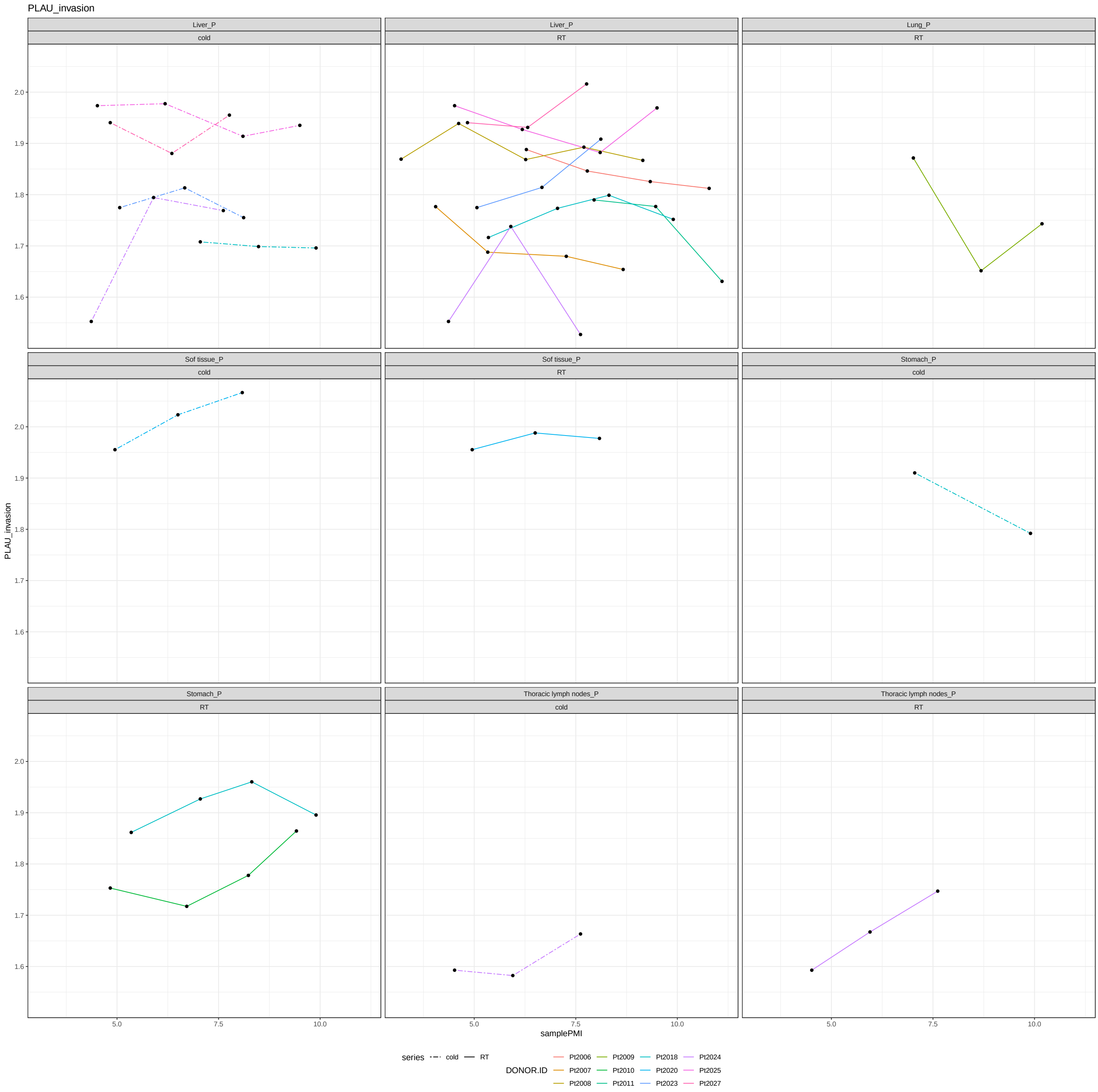
PLAU_invasion
Liver_P
cold
Liver_P
RT
Lung_P
RT
Sof tissue_P
cold
Sof tissue_P
RT
Stomach_P
cold
Stomach_P
RT
Thoracic lymph nodes_P
cold
Thoracic lymph nodes_P
RT
PLAU_invasion
samplePMI
series cold RT
DONOR.ID
Pt2006 Pt2009 Pt2018 Pt2024
Pt2007 Pt2010 Pt2020 Pt2025
Pt2008 Pt2011 Pt2023 Pt2027

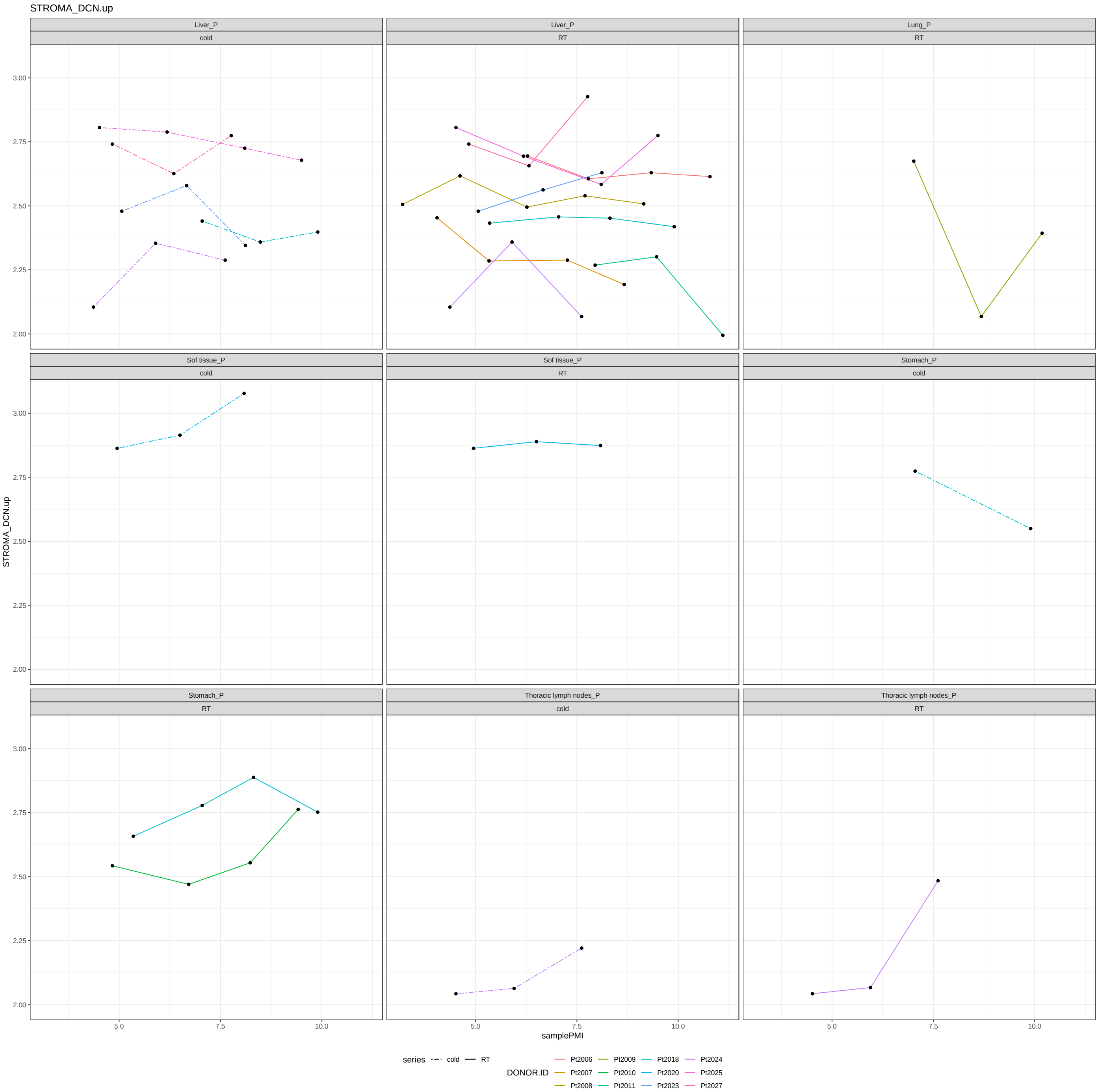

STROMA_DCN.up
Liver_P
cold
Liver_P
RT
Lung_P
RT
Sof tissue_P
cold
Sof tissue_P
RT
Stomach_P
cold
Stomach_P
RT
Thoracic lymph nodes_P
cold
Thoracic lymph nodes_P
RT
STROMA_DCN.up
samplePMI
3.00
2.75
2.50
2.25
2.00
5.0
7.5
10.0
series
cold
RT
DONOR.ID
Pt2006
Pt2007
Pt2008
Pt2009
Pt2010
Pt2011
Pt2018
Pt2020
Pt2023
Pt2024
Pt2025
Pt2027

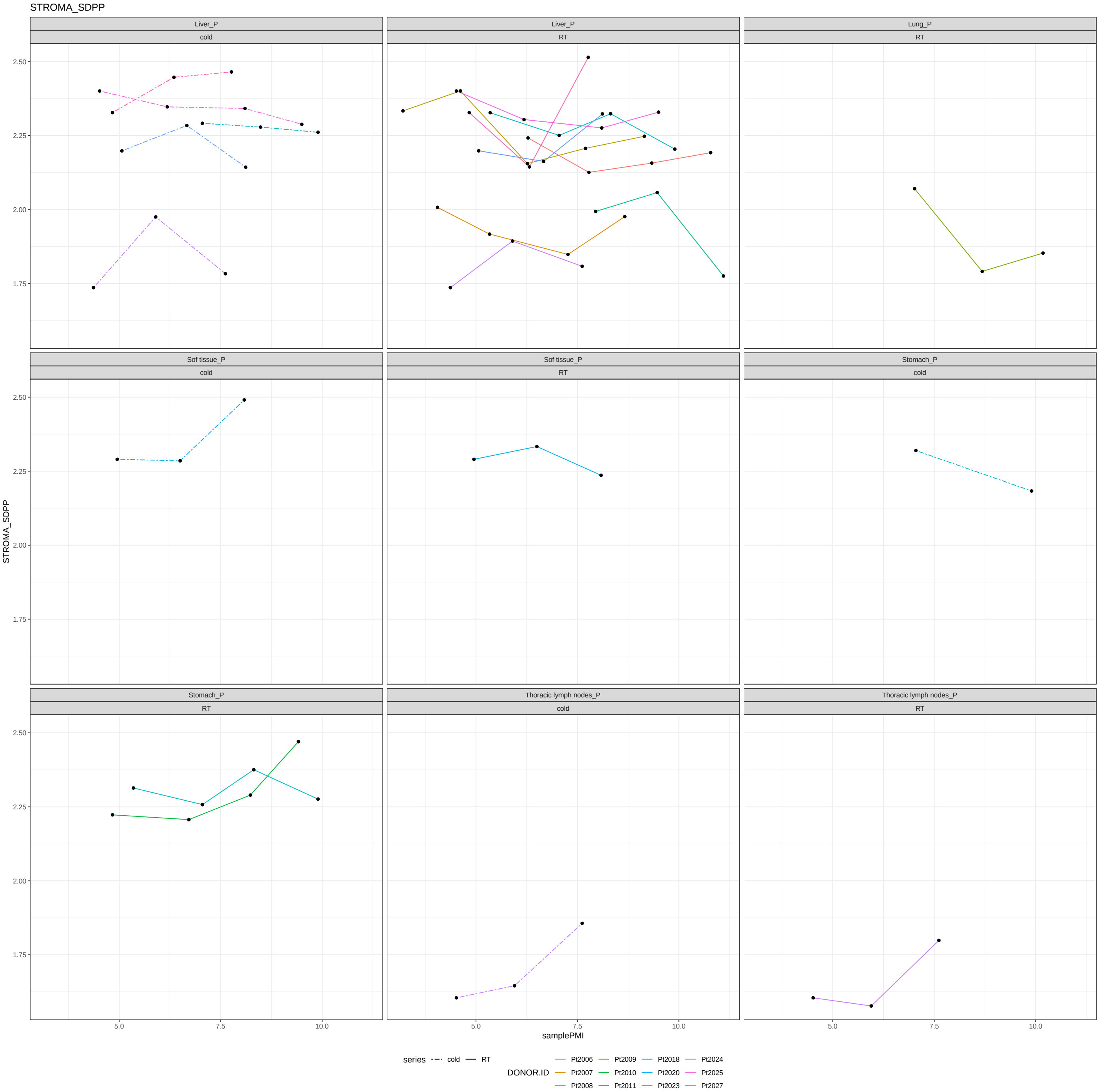
STROMA_SDPP
Liver_P
cold
Liver_P
RT
Lung_P
RT
Sof tissue_P
cold
Sof tissue_P
RT
Stomach_P
cold
Stomach_P
RT
Thoracic lymph nodes_P
cold
Thoracic lymph nodes_P
RT
STROMA_SDPP
2.50
2.25
2.00
1.75
5.0
7.5
10.0
samplePMI
series
cold
RT
DONOR.ID
Pt2006
Pt2007
Pt2008
Pt2009
Pt2010
Pt2011
Pt2018
Pt2020
Pt2023
Pt2024
Pt2025
Pt2027

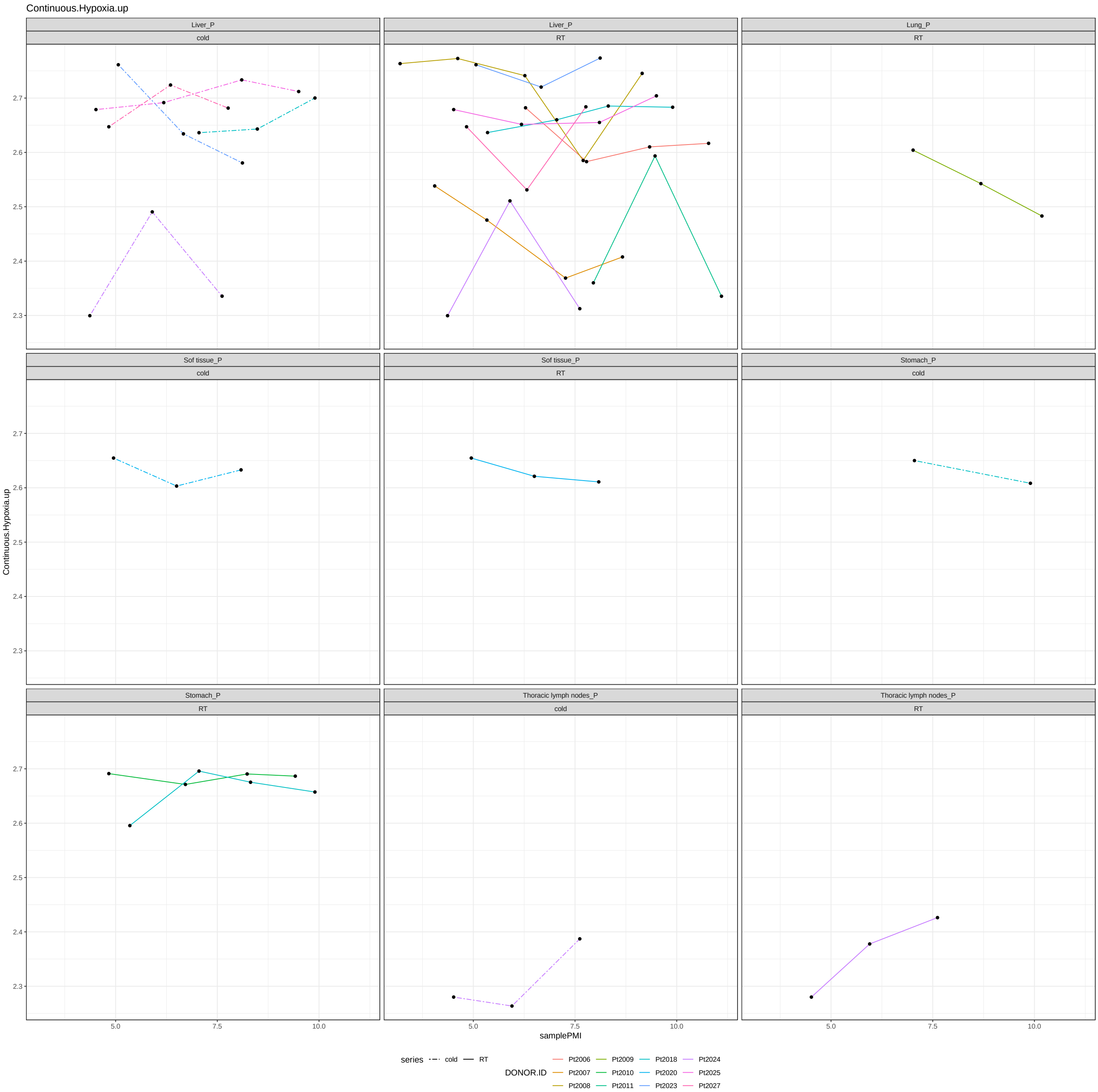
Continuous.Hypoxia.up
Liver_P
cold
RT
Lung_P
Sof tissue_P
Stomach_P
Thoracic lymph nodes_P
Continuous.Hypoxia.up
samplePMI
2.3
2.4
2.5
2.6
2.7
5.0
7.5
10.0
series
DONOR.ID
Pt2006
Pt2007
Pt2008
Pt2009
Pt2010
Pt2011
Pt2018
Pt2020
Pt2023
Pt2024
Pt2025
Pt2027

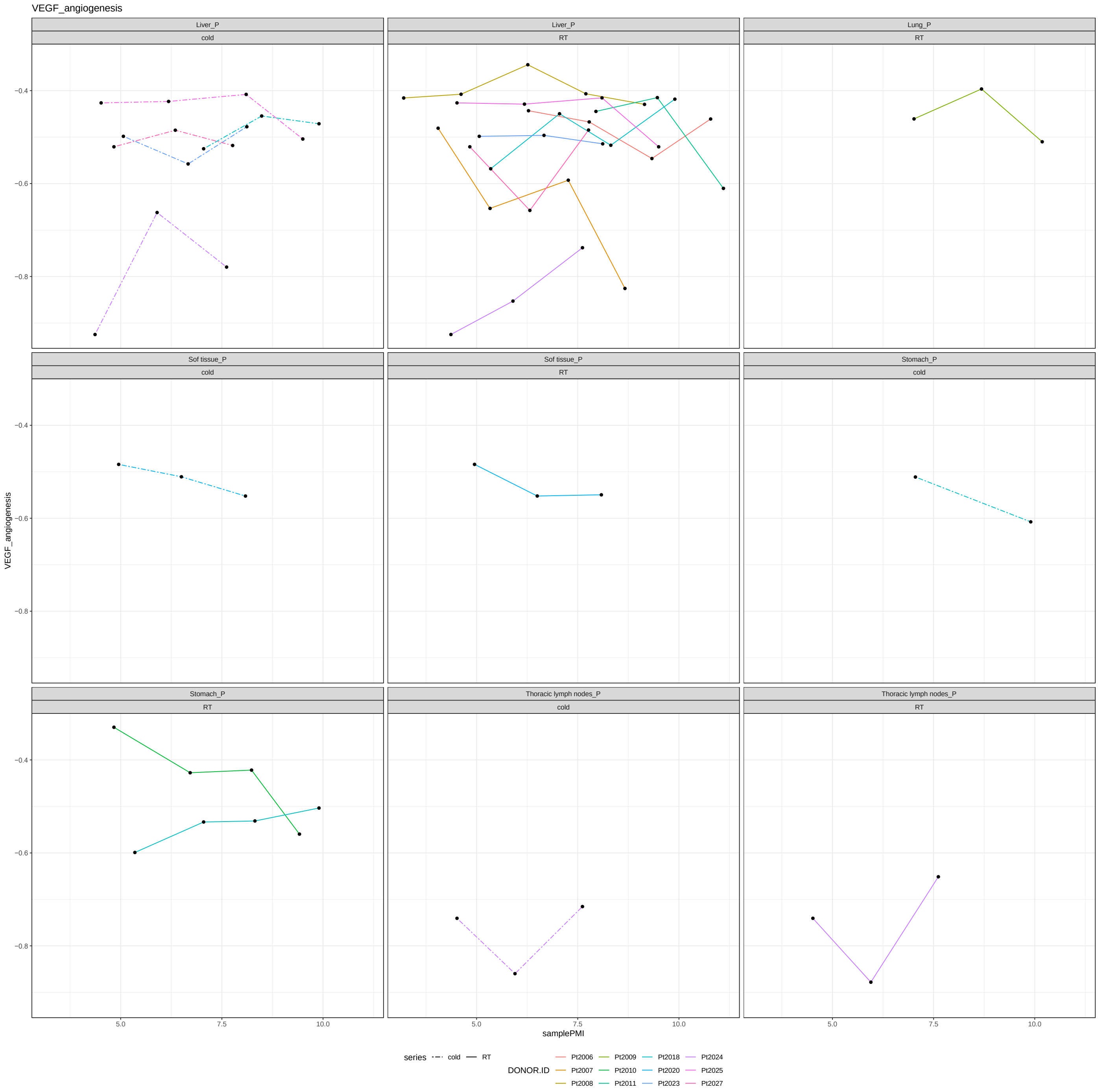

VEGF_angiogenesis
Liver_P
cold
Liver_P
RT
Lung_P
RT
Sof tissue_P
cold
Sof tissue_P
RT
Stomach_P
cold
Stomach_P
RT
Thoracic lymph nodes_P
cold
Thoracic lymph nodes_P
RT
VEGF_angiogenesis
−0.4
−0.6
−0.8
5.0
7.5
10.0
samplePMI
series
cold
RT
DONOR.ID
Pt2006
Pt2007
Pt2008
Pt2009
Pt2010
Pt2011
Pt2018
Pt2020
Pt2023
Pt2024
Pt2025
Pt2027

Hypoxia15.up

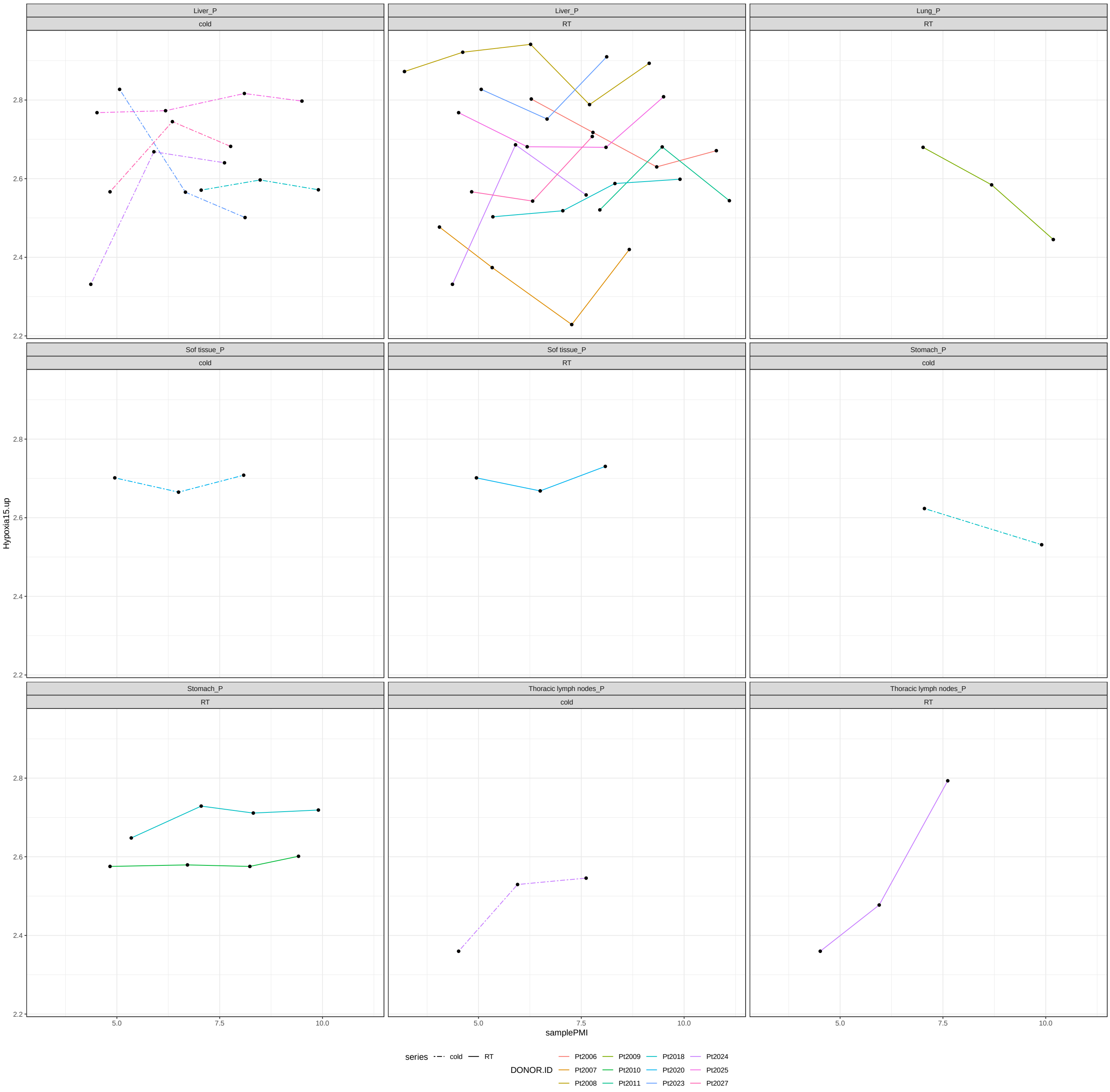

## Supplementary Figure 4

Non-linear trend observed in tumor tissue at room temperature.

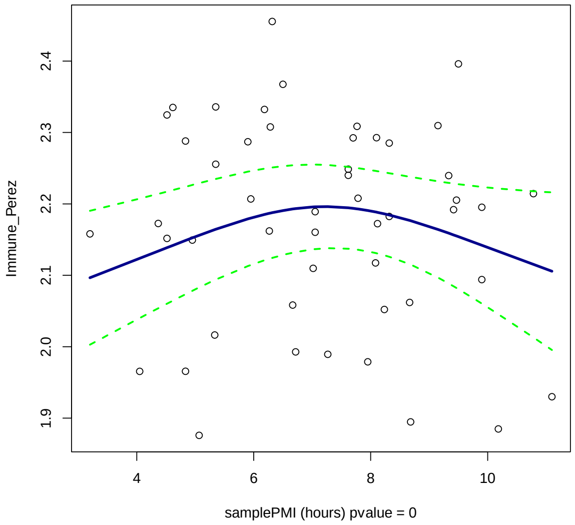


## Supplementary Figure 5

Non-linear trends observed in tumor tissue for cooled samples.

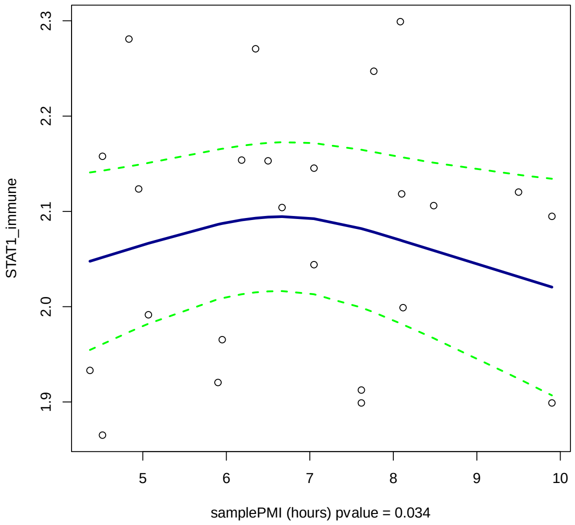

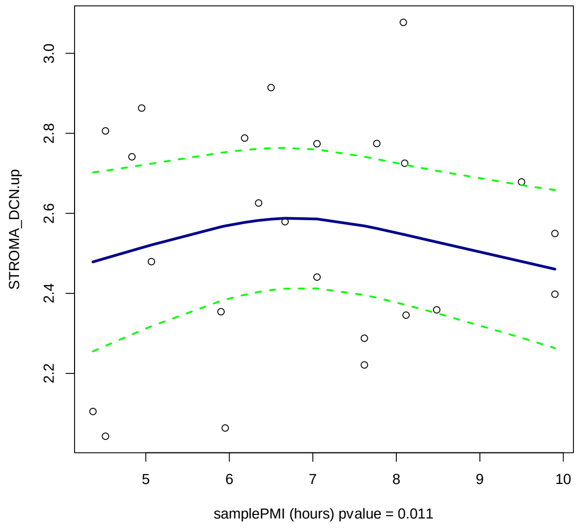
STROMA_DCN.up
3.0
2.8
2.6
2.4
2.2
5
6
7
8
9
10
samplePMI (hours) pvalue = 0.011

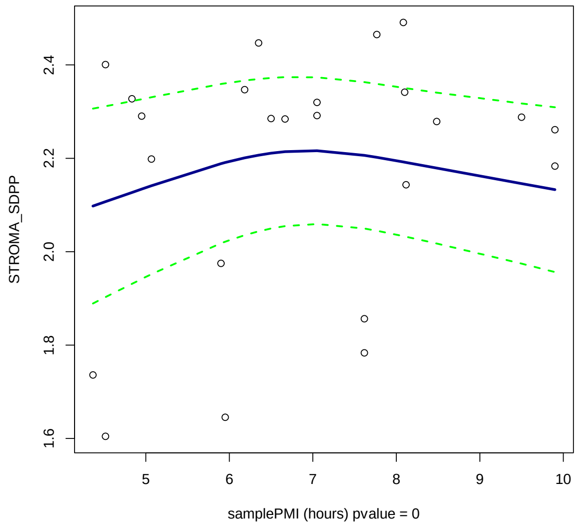
STROMA_SDPP
2.4
2.2
2.0
1.8
1.6
5
6
7
8
9
10
samplePMI (hours) pvalue = 0

# Supplementary Figure 6

Trajectories of protein expression metrics in tumor tissues.

Abbreviations: N: non-tumor tissue, P: tumor tissue, cold: samples cooled between 4°C and 10°C, RT: room temperature, samplePMI: sample specific post-mortem interval.

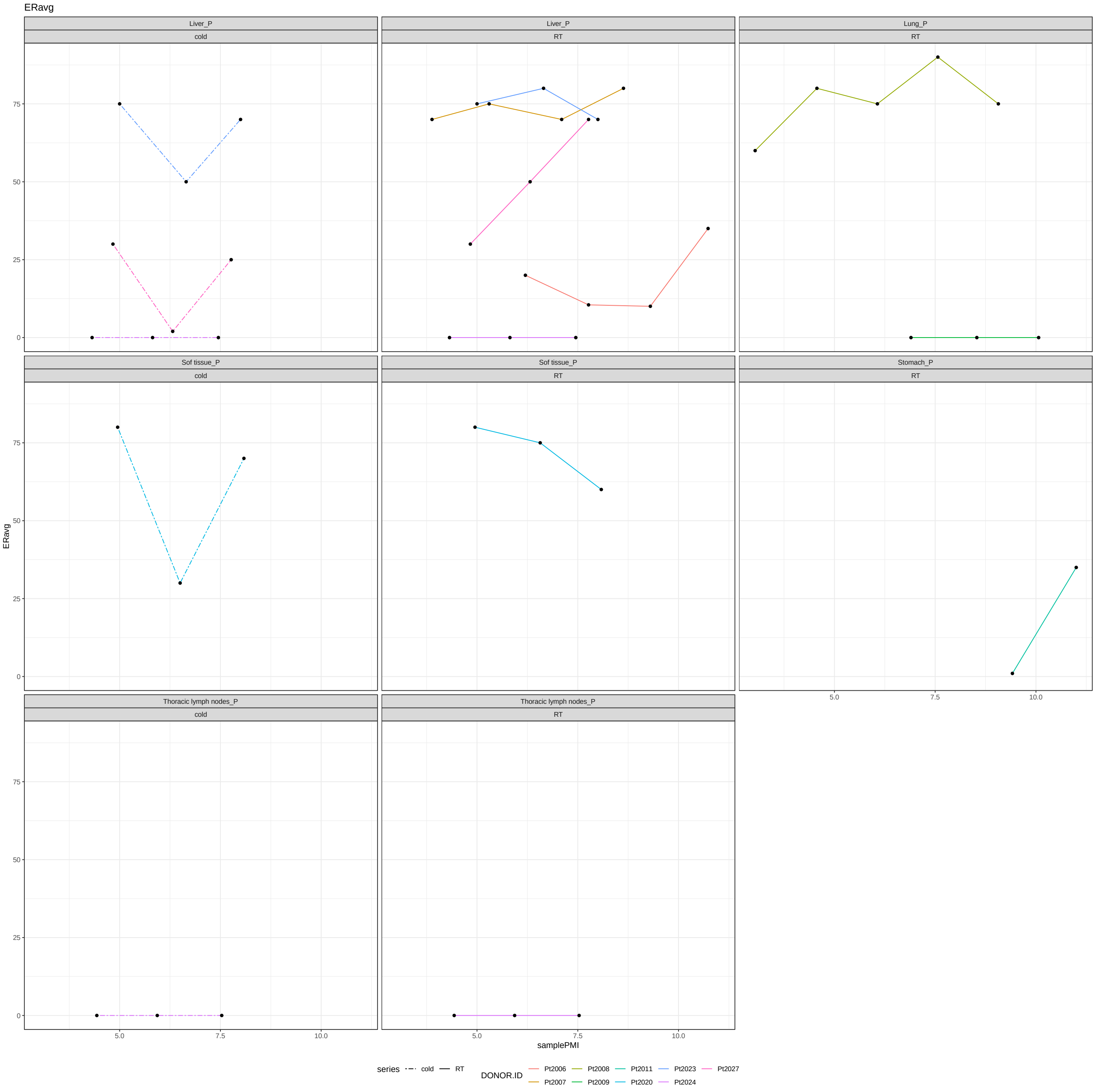

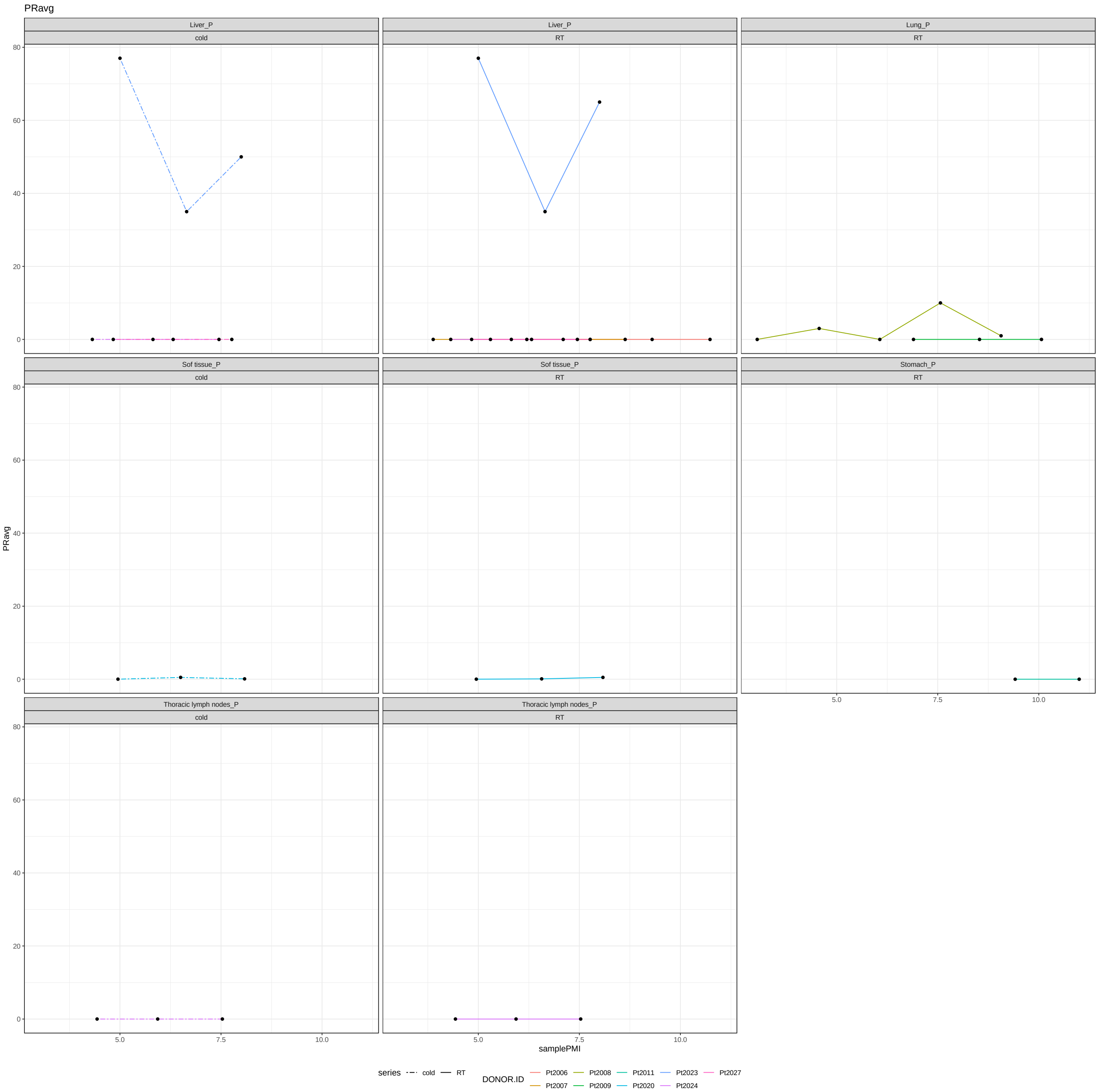

PRavg
Liver_P
cold
Liver_P
RT
Lung_P
RT
Sof tissue_P
cold
Sof tissue_P
RT
Stomach_P
RT
Thoracic lymph nodes_P
cold
Thoracic lymph nodes_P
RT
0
20
40
60
80
5.0
7.5
10.0
PRavg
samplePMI
series
cold
RT
DONOR.ID
Pt2006
Pt2007
Pt2008
Pt2009
Pt2011
Pt2020
Pt2023
Pt2024
Pt2027

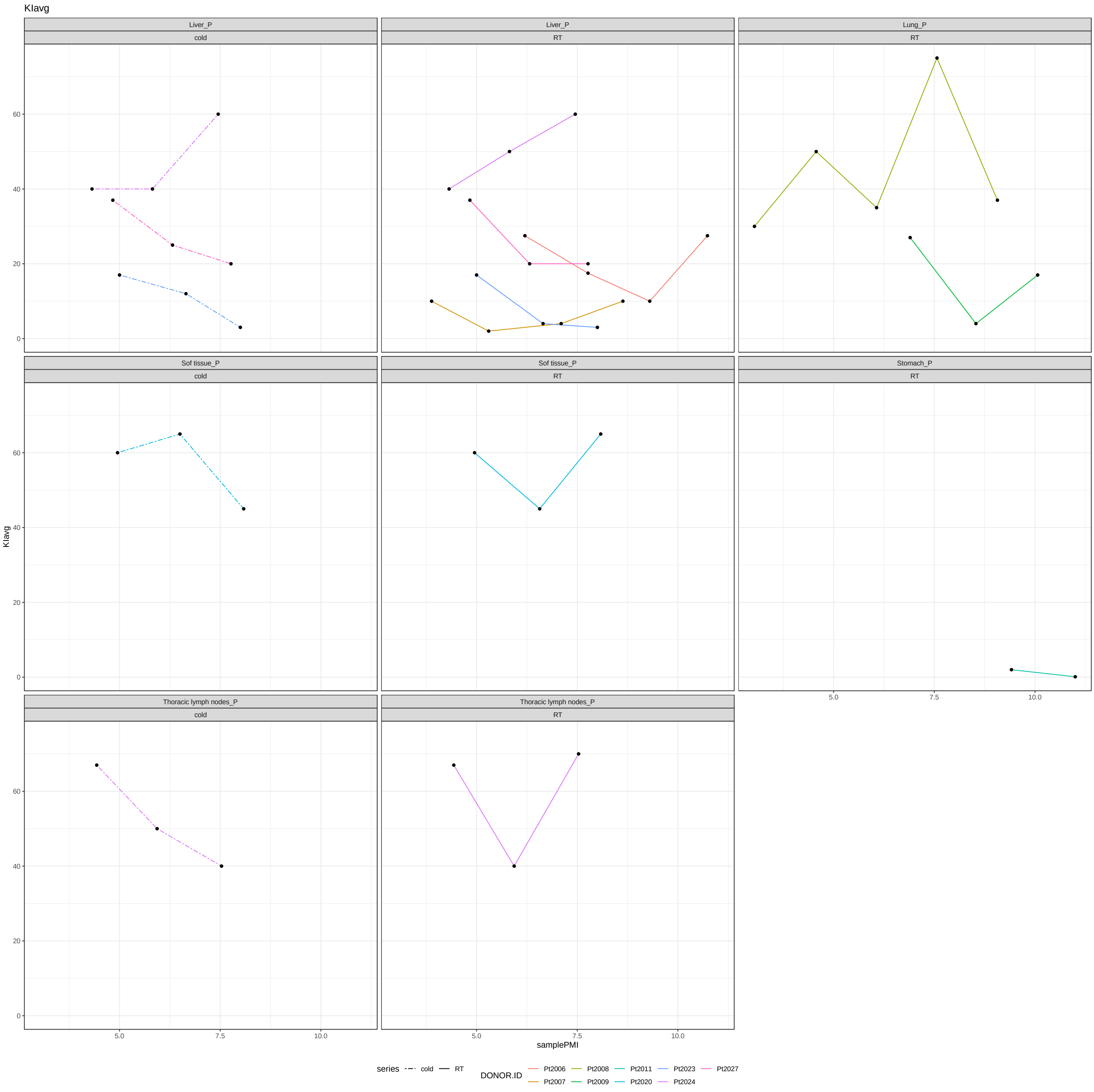

Klavg
Liver_P
cold
Liver_P
RT
Lung_P
RT
Sof tissue_P
cold
Sof tissue_P
RT
Stomach_P
RT
Thoracic lymph nodes_P
cold
Thoracic lymph nodes_P
RT
Klavg
0
20
40
60
5.0
7.5
10.0
samplePMI
series
cold
RT
DONOR.ID
Pt2006
Pt2007
Pt2008
Pt2009
Pt2011
Pt2020
Pt2023
Pt2024
Pt2027

# Supplementary Tables

## Supplementary Table 1

Clinicopathological characteristics of the patients included in this study up until January 15th 2023 (n=28), excluding the two patients who withdrew consent. IQR = Interquartile range, ER = Estrogen Receptor, PR = Progesterone Receptor. *One patient was ER-negative on all primary and metastatic samples during life and at the autopsy, except for one sample from the resection specimen of the primary tumor. She was regarded as ER-negative for the analyses in this manuscript.

| **Variable** | |
|---|---|
| **Gender** - n (%) | |
| Female | 26 (100) |
| **Age at first invasive diagnosis** - in years | |
| Median | 49,5 |
| IQR | 44,25-64,75 |
| Range | 35-80 |
| **Age at first metastasis** - in years | |
| Median | 58 |
| IQR | 47,25-69,5 |
| Range | 37-80 |
| **Time between first invasive breast cancer diagnosis and first metastasis** - in years | |
| Median | 3 |
| IQR | 1-6,75 |
| Range | 0-29 |
| **Predictive markers of the primary tumor** – n (%) | |
| ER-positive | 20 (77)* |
| PR-positive | 19 (73) |
| HER2-positive | 1 (4) |
| **Histological subtype of the primary tumor** – n (%) | |
| No Special Type (NST) | 14 (54) |
| Invasive Lobular Carcinoma (ILC) | 6 (23) |
| Mixed (NST and ILC either synchronous or metachronous) | 5 (19) |
| Metaplastic carcinoma | 1 (4) |
| **Clinical type of the primary tumor** – n (%) | |
| Inflammatory breast cancer (cT4d) | 3 (12) |

## Supplementary Table 2

Clinicopathological characteristics of the patients that underwent an autopsy within the UPTIDER program up until January 15th 2022. IQR = Interquartile range, ER = Estrogen Receptor, PR = Progesterone Receptor. *One patient was ER-negative on all primary and metastatic samples during life and at the autopsy, except for one sample from the resection specimen of the primary tumor. She was regarded as ER-negative for the analyses in this manuscript.

| **Variable** | |
|---|---|
| **Gender** - n (%) | |
| Female | 20 (100) |
| **Age at first invasive diagnosis** - in years | |
| Median | 50 |
| IQR | 44,75-64,25 |
| Range | 36-80 |
| **Age at first metastasis** - in years | |
| Median | 58 |
| IQR | 47,75-70,25 |
| Range | 37-80 |
| **Age at death** – in years | |
| Median | 60 |
| IQR | 56-77 |
| Range | 39-88 |
| **Time between first invasive diagnosis and first metastasis** - in years | |
| Median | 3 |
| IQR | 1-7 |
| Range | 0-29 |
| **Predictive markers of the primary tumor** – n (%) | |
| ER-positive | 16 (80)* |
| PR-positive | 16 (80) |
| HER2-positive | 1 (5) |
| **Histological subtype of the primary tumor** – n (%) | |
| No Special Type (NST) | 9 (45) |
| Invasive Lobular Carcinoma (ILC) | 5 (25) |
| Mixed (NST and ILC either synchronous or metachronous) | 5 (25) |
| Metaplastic carcinoma | 1 (5) |
| **Clinical type of the primary tumor** – n (%) | |
| Inflammatory breast cancer (cT4d) | 2 (10) |

## Supplementary Table 3

Details of the 15 gene signatures selected for the repeated sampling experiment. PMID = Pubmed ID of the related publication, coef = coefficient.

| **Signature:** | **ESR1_signature** | |
|---|---|---|
| PMID | 18698033 | |
| Comments | NA | |
| Genes/coef: | ESR1 | 1 |
| | TBC1D9 | 0,82 |
| | GATA3 | 0,81 |
| | CA12 | 0,77 |
| | FOXA1 | 0,75 |
| | MYB | 0,72 |
| | DNALI1 | 0,72 |
| | ABAT | 0,68 |
| | XBP1 | 0,71 |
| | SCUBE2 | 0,71 |
| | ERBB4 | 0,71 |
| | NAT1 | 0,69 |
| | AGR2 | 0,68 |
| | LRBA | 0,67 |
| | DNAJC12 | 0,65 |
| | FBP1 | 0,67 |
| | EVL | 0,65 |
| | VGLL1 | -0,66 |
| | MKL2 | 0,65 |
| | TFF1 | 0,64 |
| | CIRBP | 0,64 |
| | PHGDH | -0,65 |
| | CYP2B6 | 0,63 |
| | SOD2 | -0,63 |
| | NA | 0,63 |
| | C10orf38 | -0,62 |
| | TFF3 | 0,62 |
| | BCL11A | -0,62 |
| | ZNF552 | 0,61 |
| | KIAA1467 | 0,59 |
| | ANXA9 | 0,6 |
| | BBS1 | 0,61 |
| | NA | 0,6 |
| | HHAT | 0,58 |
| | GFRA1 | 0,58 |
| | MSN | -0,59 |
| | SCCPDH | 0,59 |
| | NA | 0,6 |
| | LRIG1 | 0,59 |

| **Signature:** | **ERBB2_signature** | |
|---|---|---|
| PMID | 18698033 | |
| Comments | NA | |
| Genes/coef: | ERBB2 | 1 |
| | PERLD1 | 0,91 |
| | PSMD3 | 0,68 |
| | PNMT | 0,65 |
| | GSDML | 0,55 |
| | CASC3 | 0,48 |
| | LASP1 | 0,47 |
| | WIPF2 | 0,44 |
| | EPN3 | 0,4 |
| | PHB | 0,4 |
| | CLCA2 | 0,36 |
| | HOXC11 | 0,31 |
| | ORMDL2 | 0,35 |
| | RAP1GAP | 0,34 |
| | CYP2J2 | 0,31 |
| | ABCA12 | 0,29 |
| | HGD | 0,3 |
| | CREG1 | -0,3 |
| | ATP2C2 | 0,29 |
| | PCTK3 | -0,29 |
| | KCTD9 | -0,29 |
| | CUEDC1 | 0,32 |
| | ITGA3 | 0,27 |
| | TMEM16K | 0,26 |
| | FJX1 | -0,25 |
| | CEACAM5 | 0,26 |
| | NR1D1 | 0,24 |
| | SNX7 | -0,23 |
| **Signature:** | **AURKA_proliferation** | |
| PMID | 18698033 | |
| Comments | NA | |
| Genes/coef: | AURKA | 1 |
| | UBE2C | 0,82 |
| | CCNB2 | 0,79 |
| | CENPA | 0,79 |
| | BIRC5 | 0,79 |
| | KIF2C | 0,78 |
| | KIF20A | 0,76 |
| | CDC20 | 0,74 |

| | | |
|---|---|---|
| | FAM63A | 0,59 |
| | KRT16 | -0,55 |
| | SSH3 | 0,58 |
| | ELOVL5 | 0,58 |
| | IRS1 | 0,57 |
| | TCF7L1 | -0,58 |
| | BTG3 | -0,58 |
| | IL6ST | 0,57 |
| | NFIL3 | -0,55 |
| | C3orf18 | 0,55 |
| | FOXC1 | -0,56 |
| | SOX11 | -0,58 |
| | PRKX | -0,56 |
| | LMO4 | -0,57 |
| | MREG | 0,57 |
| | IFT88 | 0,55 |
| | GARS | -0,56 |
| | LDHB | -0,56 |
| | NME5 | 0,56 |
| | HEXIM1 | 0,55 |
| | MTL5 | 0,56 |
| | GABRP | -0,56 |
| | MAN2B2 | 0,56 |
| | C1orf106 | -0,54 |
| | SERPINA5 | 0,55 |
| | PDZK1 | 0,55 |
| | TMEM9B | 0,53 |
| | CEBPG | -0,55 |
| | SLC16A6 | 0,55 |
| | ROGDI | 0,55 |
| | KIF13B | 0,54 |
| | FABP7 | -0,53 |
| | GPD1L | 0,54 |
| | KIAA0040 | 0,53 |
| | TJP3 | 0,54 |
| | TCEAL4 | 0,54 |
| | ELOVL2 | 0,53 |
| | COX6C | 0,54 |
| | RBMS1 | -0,54 |
| | NA | 0,54 |
| | LAD1 | -0,54 |
| | FUT8 | 0,51 |
| | C5orf5 | 0,53 |
| | RAB26 | 0,53 |
| | MAPRE2 | -0,52 |

| | | |
|---|---|---|
| | FOXM1 | 0,74 |
| | CCNB1 | 0,75 |
| | TPX2 | 0,75 |
| | PKMYT1 | 0,7 |
| | FAM64A | 0,69 |
| | NEK2 | 0,72 |
| | KIF4A | 0,71 |
| | NCAPH | 0,72 |
| | TRIP13 | 0,71 |
| | MAD2L1 | 0,7 |
| | EXO1 | 0,67 |
| | SPAG5 | 0,67 |
| | TK1 | 0,64 |
| | STIL | 0,68 |
| | RRM2 | 0,66 |
| | CENPN | 0,67 |
| | TYMS | 0,66 |
| | BLM | 0,65 |
| | MCM2 | 0,64 |
| | CKS2 | 0,61 |
| | MLF1IP | 0,62 |
| | FRY | -0,59 |
| | GINS2 | 0,58 |
| | DDX39 | 0,57 |
| | STMN1 | 0,59 |
| | SHCBP1 | 0,59 |
| | H2AFX | 0,58 |
| | RNASEH2A | 0,58 |
| | RFC4 | 0,58 |
| | GNG12 | -0,56 |
| | CDKN3 | 0,58 |
| | MCM10 | 0,57 |
| | C20orf20 | 0,55 |
| | CKS1B | 0,55 |
| | TIMELESS | 0,56 |
| | NA | 0,51 |
| | TENC1 | -0,54 |
| | PIR | 0,52 |
| | CDC7 | 0,52 |
| | FMOD | -0,5 |
| | GTSE1 | 0,52 |
| | SFRP4 | -0,5 |
| | LAPTM4B | 0,51 |
| | SPARCL1 | -0,51 |
| | CDC6 | 0,5 |

| | | |
|---|---|---|
| | DCI | 0,52 |
| | REEP5 | 0,53 |
| | CHAD | 0,53 |
| | APBB2 | 0,51 |
| | CCDC56 | 0,52 |
| | CSTB | -0,52 |
| | CHST2 | -0,52 |
| | UCK2 | -0,52 |
| | GLI3 | 0,52 |
| | MARCO | -0,52 |
| | HSD17B4 | 0,5 |
| | TMSL8 | -0,48 |
| | PIGT | 0,51 |
| | SOX10 | -0,52 |
| | MICALL1 | -0,52 |
| | OVOL2 | 0,51 |
| | CHI3L1 | -0,51 |
| | SLC27A2 | 0,5 |
| | ABTB2 | -0,5 |
| | RBKS | 0,5 |
| | SFT2D2 | -0,49 |
| | SLC9A6 | -0,46 |
| | SLC22A18 | 0,5 |
| | KCNK5 | -0,51 |
| | HDAC11 | 0,5 |
| | SLC6A14 | -0,47 |
| | C14orf79 | 0,51 |
| | C16orf57 | -0,51 |
| | TRIM2 | -0,51 |
| | NEDD4L | 0,5 |
| | AKR7A3 | 0,5 |
| | LY6D | -0,5 |
| | PROM1 | -0,5 |
| | ODC1 | -0,5 |
| | RBM38 | -0,49 |
| | ZNF446 | 0,5 |
| | PIB5PA | 0,49 |
| | SRD5A1 | -0,5 |
| | GALNT7 | 0,49 |
| | C16orf45 | 0,49 |
| | DSCR1 | -0,45 |
| | CYB5R1 | 0,48 |
| | ICAM1 | -0,49 |
| | PTX3 | -0,5 |
| | RPH3AL | 0,49 |

| | | |
|---|---|---|
| | TGFB3 | -0,5 |
| | ADRM1 | 0,48 |
| | CTDSP1 | -0,49 |
| | TMC5 | -0,49 |
| | LMNB2 | 0,49 |
| | C5orf21 | -0,49 |
| | DOCK1 | -0,48 |
| | ITGBL1 | -0,44 |
| | GGH | 0,48 |
| | PLSCR4 | -0,48 |
| | SNX1 | -0,47 |
| | OGN | -0,47 |
| | STARD13 | -0,48 |
| | XPOT | 0,47 |
| | NA | 0,48 |
| | CKAP5 | 0,47 |
| | GNG11 | -0,44 |
| | NEIL3 | 0,39 |
| | LRRC17 | -0,47 |
| | SLIT2 | -0,45 |
| | DTYMK | 0,45 |
| | NUP155 | 0,46 |
| | POLD1 | 0,44 |
| | SNRPC | 0,44 |
| | LHFP | -0,45 |
| | NUDT1 | 0,45 |
| | IGF1 | -0,45 |
| | MCM3 | 0,45 |
| | FCER1A | -0,45 |
| | C1orf112 | 0,43 |
| | DIRAS3 | -0,45 |
| | PRIM1 | 0,45 |
| | UCKL1 | 0,44 |
| | EXOSC4 | 0,44 |
| | CYBRD1 | -0,44 |
| | P2RY5 | -0,44 |
| | CNIH4 | 0,41 |
| | SLC2A1 | 0,44 |
| | ZNF706 | 0,43 |
| | CAV1 | -0,42 |
| | LACTB2 | 0,38 |
| | CCDC44 | 0,41 |
| | RBM35A | 0,4 |
| | ANG | -0,41 |
| | C16orf30 | -0,41 |

| | | |
|---|---|---|
| | IMPA2 | -0,49 |
| | ZNF20 | 0,47 |
| | S100A9 | -0,49 |
| | SEPHS1 | -0,49 |
| | C1orf21 | 0,49 |
| | CREBL2 | 0,47 |
| | CRYAB | -0,49 |
| | MRPS30 | 0,48 |
| | C20orf23 | 0,49 |
| | DSC2 | -0,49 |
| | MALL | -0,49 |
| | GLRB | 0,48 |
| | ASAH1 | 0,47 |
| | PGR | 0,51 |
| | ZMYND10 | 0,47 |
| | TBX3 | 0,47 |
| | PEX12 | 0,47 |
| | CSDA | -0,48 |
| | ABCF1 | -0,46 |
| | ZIC1 | -0,48 |
| | CAMLG | 0,47 |
| | GSTM3 | 0,48 |
| | ABCD3 | 0,48 |
| | QDPR | 0,47 |
| | SCPEP1 | -0,47 |
| | CALML5 | -0,44 |
| | LASS4 | 0,44 |
| | ABCA3 | 0,48 |
| | SIDT1 | 0,46 |
| | BCAS1 | 0,47 |
| | NMU | -0,41 |
| | C21orf91 | -0,46 |
| | JOSD1 | -0,46 |
| | PKP1 | -0,48 |
| | UQCRH | -0,46 |
| | CREB3L2 | -0,45 |
| | ZNF263 | 0,46 |
| | COASY | 0,44 |
| | C19orf21 | 0,45 |
| | HEBP1 | 0,45 |
| | AFTPH | 0,46 |
| | MCCC2 | 0,46 |
| | SLC5A6 | -0,44 |
| | SERPINB6 | 0,46 |
| | MMP12 | -0,44 |

| | | |
|---|---|---|
| | OLFML3 | -0,4 |
| | HSF1 | 0,39 |
| | COQ2 | 0,39 |
| | FRZB | -0,4 |
| | HPRT1 | 0,4 |
| | PCOLN3 | 0,4 |
| | SUV39H1 | 0,4 |
| | RBMS3 | -0,38 |
| | FST | -0,38 |
| | AK5 | -0,4 |
| | CDCA4 | 0,39 |
| | TUBG1 | 0,38 |
| | RRS1 | 0,38 |
| | JMJD4 | 0,39 |
| | LRRC59 | 0,37 |
| | NA | -0,3 |
| | GLRX2 | 0,37 |
| | YTHDF1 | 0,37 |
| | SNRK | -0,37 |
| | C1orf135 | 0,35 |
| | ZFHX4 | -0,38 |
| | GPR177 | -0,34 |
| | ZNF165 | 0,34 |
| | STRA13 | 0,36 |
| | DUSP6 | -0,34 |
| | SEMA5A | -0,38 |
| | POLR2D | 0,39 |
| | THYN1 | -0,32 |
| | C11orf63 | -0,36 |
| | AQP1 | -0,32 |
| | SNRPD3 | 0,36 |
| | GAS6 | -0,35 |
| | WDR45L | 0,34 |
| | PER1 | -0,36 |
| | ESD | -0,33 |
| | LAS1L | 0,36 |
| | SLC26A3 | -0,32 |
| | CCHCR1 | 0,3 |
| | DHTKD1 | 0,3 |
| | PELI2 | -0,34 |
| | FOS | -0,35 |
| | C1RL | -0,35 |
| | TGIF2 | 0,35 |
| | HSD17B10 | 0,34 |
| | TIMM10 | 0,34 |

| | | |
|---|---|---|
| | MIA | -0,43 |
| | STAC | -0,46 |
| | TEX264 | 0,44 |
| | SEC14L2 | 0,45 |
| | REPS2 | 0,44 |
| | AGTR1 | 0,45 |
| | UGT8 | -0,47 |
| | FAM102A | 0,43 |
| | SERPINA3 | 0,43 |
| | KLHL7 | -0,45 |
| | ABHD14A | 0,43 |
| | NOTCH1 | -0,45 |
| | PIGQ | 0,45 |
| | COL9A3 | -0,43 |
| | GOLGA1 | 0,43 |
| | FZD9 | -0,47 |
| | CX3CL1 | -0,45 |
| | PLA2G10 | 0,44 |
| | PLAT | 0,45 |
| | ABLIM3 | 0,45 |
| | C9orf7 | 0,44 |
| | PLA2G4A | -0,42 |
| | TTYH1 | -0,45 |
| | NEK4 | 0,44 |
| | LRRC50 | 0,42 |
| | CD2BP2 | 0,44 |
| | RELB | -0,42 |
| | ABCC8 | 0,43 |
| | PTPRT | 0,44 |
| | TRIM29 | -0,41 |
| | OCEL1 | 0,4 |
| | CTSF | 0,44 |
| | HRASLS | -0,43 |
| | SPR | 0,37 |
| | CXCL1 | -0,43 |
| | PDCD4 | 0,42 |
| | ASTN2 | 0,43 |
| | IRX5 | 0,44 |
| | GPM6B | -0,43 |
| | IGF2BP2 | -0,4 |
| | VLDLR | -0,41 |
| | BACE2 | -0,43 |
| | C5orf3 | 0,43 |
| | NMB | -0,43 |
| | SREBF1 | 0,42 |

| | | |
|---|---|---|
| | GPR124 | -0,34 |
| | SPRY1 | -0,35 |
| | RPS6KB2 | 0,32 |
| | ARHGAP11A | 0,27 |
| | SLC39A4 | 0,35 |
| | TGFBR3 | -0,33 |
| | RUVBL1 | 0,27 |
| | GALC | -0,34 |
| | RUNX1T1 | -0,35 |
| | TTF2 | 0,33 |
| | EIF4A3 | 0,33 |
| | HNRPA2B1 | 0,33 |
| | SS18L1 | 0,32 |
| | SORBS1 | -0,34 |
| | THBD | -0,31 |
| | FZD4 | -0,35 |
| | CDH5 | -0,33 |
| | F3 | -0,33 |
| | NA | -0,3 |
| | GINS3 | 0,35 |
| | TSEN34 | 0,32 |
| | TRIM24 | 0,32 |
| | CSTF2 | 0,32 |
| | DLX2 | -0,32 |
| | PLXDC1 | -0,28 |
| | LIMCH1 | -0,31 |
| | C17orf41 | 0,3 |
| | NR2F2 | -0,32 |
| | WNT5A | -0,31 |
| | C20orf19 | -0,33 |
| | ERMAP | -0,29 |
| | FCGBP | -0,31 |
| | STEAP1 | -0,3 |
| | TRPM2 | 0,29 |
| | TFPT | 0,27 |
| | NDUFS1 | 0,3 |
| | LOH11CR2A | -0,3 |
| | ICT1 | 0,29 |
| | ARHGDIB | -0,28 |
| | EMG1 | 0,3 |
| | AZIN1 | 0,29 |
| | AHSA1 | 0,29 |
| | APLP1 | 0,27 |
| | CHCHD2 | 0,29 |
| | DKK2 | -0,29 |

| | | |
|---|---|---|
| | UBE2E3 | -0,42 |
| | HDAC2 | -0,43 |
| | ETNK2 | 0,4 |
| | CBS | -0,36 |
| | KRT17 | -0,4 |
| | C18orf1 | 0,42 |
| | ADORA2B | -0,42 |
| | EMP2 | 0,42 |
| | EEF1A2 | 0,43 |
| | IL8 | -0,42 |
| | ART3 | -0,43 |
| | PIGV | 0,42 |
| | MRPS27 | 0,41 |
| | KLK5 | -0,41 |
| | ACOX2 | 0,41 |
| | CLGN | 0,37 |
| | NR2E3 | 0,41 |
| | TRMT11 | -0,41 |
| | KHDRBS3 | -0,4 |
| | STEAP3 | -0,41 |
| | HSPB1 | 0,41 |
| | STUB1 | 0,41 |
| | FABP5 | -0,41 |
| | C20orf12 | 0,4 |
| | PTPN13 | 0,39 |
| | E4F1 | 0,4 |
| | PRSS23 | 0,41 |
| | DHRS2 | 0,39 |
| | RAB11FIP1 | 0,41 |
| | OGFRL1 | -0,41 |
| | TCN2 | -0,4 |
| | HIVEP2 | -0,4 |
| | PLOD3 | -0,41 |
| | KRT86 | -0,41 |
| | CCT4 | -0,4 |
| | COQ6 | 0,4 |
| | TM4SF1 | -0,4 |
| | DHCR24 | 0,38 |
| | CRABP1 | -0,4 |
| | SLC9A3R1 | 0,41 |
| | TTC30A | 0,4 |
| | GDF15 | 0,4 |
| | CCL20 | -0,36 |
| | HSPA2 | 0,4 |
| | PGBD5 | -0,41 |

| | | |
|---|---|---|
| | NA | -0,28 |
| | ABCA6 | -0,27 |
| | SOX17 | -0,28 |
| | TLR3 | -0,27 |
| | SCNN1B | 0,29 |
| | ITGB4BP | 0,26 |
| | SPRY2 | -0,29 |
| | GEM | -0,28 |
| | VTCN1 | -0,26 |
| | HMBOX1 | -0,27 |
| | FADD | 0,27 |
| | RCE1 | 0,26 |
| | ZNF250 | 0,25 |
| | KERA | -0,32 |
| | THOP1 | 0,21 |
| | CACNA2D3 | -0,26 |
| | ZNF291 | -0,26 |
| | DDX56 | 0,2 |
| | TPD52L1 | 0,26 |
| | TMEM177 | 0,27 |
| | DST | -0,25 |
| | GNAZ | 0,26 |
| | GCAT | 0,25 |
| | ISOC2 | 0,26 |
| | MYO9A | -0,25 |
| | DSCR6 | 0,23 |
| | INSIG1 | 0,28 |
| | STRAP | 0,25 |
| | SF3B2 | 0,25 |
| | SUPV3L1 | 0,25 |
| | NKRF | 0,24 |
| | RPP38 | 0,27 |
| | HOXB6 | -0,25 |
| | C17orf59 | -0,24 |
| | EEF1B2 | -0,24 |
| | COIL | 0,23 |
| | BCKDHB | -0,26 |
| | S100P | 0,23 |
| | LETM1 | 0,23 |
| | YBX2 | 0,2 |
| | TOMM34 | 0,2 |
| **Signature:** | **AURKA_proliferation** | |
| PMID | 18698033 | |
| Comments | NA | |
| Genes/coef: | AURKA | 1 |

| | | |
|---|---|---|
| | DMN | -0,39 |
| | CP | -0,4 |
| | CCNJ | -0,38 |
| | PTPLA | -0,4 |
| | CYP39A1 | -0,34 |
| | PEX7 | 0,4 |
| | TSPO | -0,39 |
| | TP53BP2 | -0,4 |
| | EXDL2 | 0,4 |
| | C3orf52 | 0,39 |
| | TOB1 | 0,37 |
| | GALNT6 | 0,4 |
| | SORD | 0,39 |
| | IDH2 | -0,4 |
| | MPHOSPH6 | -0,4 |
| | UCHL1 | -0,38 |
| | SLC15A1 | -0,34 |
| | PRR13 | 0,39 |
| | NDUFAF1 | 0,35 |
| | NA | 0,39 |
| | TOM1L1 | 0,38 |
| | CHI3L2 | -0,38 |
| | EGLN2 | 0,39 |
| | MFHAS1 | -0,32 |
| | AREG | 0,38 |
| | GUCY1A3 | -0,38 |
| | KLF5 | -0,39 |
| | EGR3 | 0,37 |
| | UBXD6 | 0,38 |
| | TPSG1 | 0,37 |
| | MST1 | 0,36 |
| | ZNF281 | 0,38 |
| | DEFB1 | -0,39 |
| | DCPS | -0,38 |
| | SERPINB5 | -0,36 |
| | CD24 | -0,36 |
| | CAMK2N1 | 0,37 |
| | RYR1 | -0,35 |
| | GATA6 | -0,37 |
| | ACTR3B | -0,39 |
| | LRP2 | 0,35 |
| | MYLIP | 0,37 |
| | GPR126 | -0,38 |
| | CITED1 | 0,38 |
| | RPP25 | -0,37 |

| | | |
|---|---|---|
| | UBE2C | 0,82 |
| | CCNB2 | 0,79 |
| | CENPA | 0,79 |
| | BIRC5 | 0,79 |
| | KIF2C | 0,78 |
| | KIF20A | 0,76 |
| | CDC20 | 0,74 |
| | FOXM1 | 0,74 |
| | CCNB1 | 0,75 |
| | TPX2 | 0,75 |
| | PKMYT1 | 0,7 |
| | FAM64A | 0,69 |
| | NEK2 | 0,72 |
| | KIF4A | 0,71 |
| | NCAPH | 0,72 |
| | TRIP13 | 0,71 |
| | MAD2L1 | 0,7 |
| | EXO1 | 0,67 |
| | SPAG5 | 0,67 |
| | TK1 | 0,64 |
| | STIL | 0,68 |
| | RRM2 | 0,66 |
| | CENPN | 0,67 |
| | TYMS | 0,66 |
| | BLM | 0,65 |
| | MCM2 | 0,64 |
| | CKS2 | 0,61 |
| | MLF1IP | 0,62 |
| | FRY | -0,59 |
| | GINS2 | 0,58 |
| | DDX39 | 0,57 |
| | STMN1 | 0,59 |
| | SHCBP1 | 0,59 |
| | H2AFX | 0,58 |
| | RNASEH2A | 0,58 |
| | RFC4 | 0,58 |
| | GNG12 | -0,56 |
| | CDKN3 | 0,58 |
| | MCM10 | 0,57 |
| | C20orf20 | 0,55 |
| | CKS1B | 0,55 |
| | TIMELESS | 0,56 |
| | NA | 0,51 |
| | TENC1 | -0,54 |
| | PIR | 0,52 |

| | | |
|---|---|---|
| | FGFBP1 | -0,33 |
| | FAM107A | -0,36 |
| | HSD17B2 | -0,38 |
| | SLC22A4 | 0,32 |
| | ATAD4 | 0,37 |
| | CLIP4 | -0,28 |
| | GP2 | 0,35 |
| | SRM | -0,35 |
| | CPB1 | 0,35 |
| | OVGP1 | 0,34 |
| | SERPINB8 | -0,36 |
| | AMIGO2 | 0,36 |
| | NA | 0,36 |
| | NA | 0,36 |
| | THOC5 | -0,36 |
| | APH1B | 0,38 |
| | PRAME | -0,35 |
| | SURF1 | 0,36 |
| | RLN2 | 0,34 |
| | ALCAM | 0,36 |
| | SYNJ2BP | 0,35 |
| | HYOU1 | -0,35 |
| | FDXR | 0,36 |
| | BIN1 | -0,36 |
| | APBB3 | 0,35 |
| | RPS6KA4 | -0,34 |
| | ARNTL2 | -0,35 |
| | ADAMTS1 | -0,32 |
| | GNAI1 | -0,34 |
| | MPG | 0,34 |
| | CBFA2T3 | 0,34 |
| | GRIA2 | 0,33 |
| | UNG2 | 0,34 |
| | TFRC | -0,34 |
| | IL7 | -0,34 |
| | UEVLD | 0,34 |
| | GLS2 | 0,25 |
| | RIC8B | 0,34 |
| | KLK8 | -0,36 |
| | DCXR | 0,3 |
| | CAPN6 | -0,33 |
| | RND3 | -0,34 |
| | GFOD1 | -0,34 |
| | ATP9A | 0,33 |
| | MIPEP | 0,36 |

| | | |
|---|---|---|
| | CDC7 | 0,52 |
| | FMOD | -0,5 |
| | GTSE1 | 0,52 |
| | SFRP4 | -0,5 |
| | LAPTM4B | 0,51 |
| | SPARCL1 | -0,51 |
| | CDC6 | 0,5 |
| | TGFB3 | -0,5 |
| | ADRM1 | 0,48 |
| | CTDSP1 | -0,49 |
| | TMC5 | -0,49 |
| | LMNB2 | 0,49 |
| | C5orf21 | -0,49 |
| | DOCK1 | -0,48 |
| | ITGBL1 | -0,44 |
| | GGH | 0,48 |
| | PLSCR4 | -0,48 |
| | SNX1 | -0,47 |
| | OGN | -0,47 |
| | STARD13 | -0,48 |
| | XPOT | 0,47 |
| | NA | 0,48 |
| | CKAP5 | 0,47 |
| | GNG11 | -0,44 |
| | NEIL3 | 0,39 |
| | LRRC17 | -0,47 |
| | SLIT2 | -0,45 |
| | DTYMK | 0,45 |
| | NUP155 | 0,46 |
| | POLD1 | 0,44 |
| | SNRPC | 0,44 |
| | LHFP | -0,45 |
| | NUDT1 | 0,45 |
| | IGF1 | -0,45 |
| | MCM3 | 0,45 |
| | FCER1A | -0,45 |
| | C1orf112 | 0,43 |
| | DIRAS3 | -0,45 |
| | PRIM1 | 0,45 |
| | UCKL1 | 0,44 |
| | EXOSC4 | 0,44 |
| | CYBRD1 | -0,44 |
| | P2RY5 | -0,44 |
| | CNIH4 | 0,41 |
| | SLC2A1 | 0,44 |

| | | |
|---|---|---|
| | FZD7 | -0,33 |
| | GPRC5A | 0,35 |
| | ADAMTS3 | -0,29 |
| | CIB1 | 0,32 |
| | TNNT1 | 0,33 |
| | RAPGEF6 | 0,33 |
| | TTC12 | 0,29 |
| | GALNT3 | -0,34 |
| | FOLR1 | -0,33 |
| | GSTZ1 | 0,33 |
| | ZCCHC11 | -0,29 |
| | RAMP1 | 0,33 |
| | KRT23 | -0,34 |
| | SIX3 | -0,26 |
| | CACNG1 | 0,33 |
| | PAXIP1 | 0,31 |
| | TMEM80 | 0,33 |
| | CFB | 0,33 |
| | TUFT1 | 0,32 |
| | PRKAA1 | -0,27 |
| | KIAA0753 | 0,34 |
| | C16orf33 | 0,31 |
| | RSAD1 | 0,33 |
| | S100A1 | -0,33 |
| | BEX1 | 0,32 |
| | IL12RB2 | -0,34 |
| | PCDH8 | -0,36 |
| | GPR37 | -0,32 |
| | SACS | -0,3 |
| | H2AFY2 | -0,31 |
| | DNAJC1 | 0,31 |
| | HIVEP1 | -0,3 |
| | CLUAP1 | 0,31 |
| | ADIPOR2 | 0,29 |
| | C21orf59 | 0,3 |
| | DKK1 | -0,32 |
| | S100A6 | -0,31 |
| | GRAMD3 | -0,31 |
| | MGMT | 0,31 |
| | WFDC2 | 0,31 |
| | KCNJ3 | 0,29 |
| | CCRK | 0,28 |
| | NOS1AP | 0,23 |
| | PRKCBP1 | 0,3 |
| | AIM1 | -0,28 |

| | | |
|---|---|---|
| | ZNF706 | 0,43 |
| | CAV1 | -0,42 |
| | LACTB2 | 0,38 |
| | CCDC44 | 0,41 |
| | RBM35A | 0,4 |
| | ANG | -0,41 |
| | C16orf30 | -0,41 |
| | OLFML3 | -0,4 |
| | HSF1 | 0,39 |
| | COQ2 | 0,39 |
| | FRZB | -0,4 |
| | HPRT1 | 0,4 |
| | PCOLN3 | 0,4 |
| | SUV39H1 | 0,4 |
| | RBMS3 | -0,38 |
| | FST | -0,38 |
| | AK5 | -0,4 |
| | CDCA4 | 0,39 |
| | TUBG1 | 0,38 |
| | RRS1 | 0,38 |
| | JMJD4 | 0,39 |
| | LRRC59 | 0,37 |
| | NA | -0,3 |
| | GLRX2 | 0,37 |
| | YTHDF1 | 0,37 |
| | SNRK | -0,37 |
| | C1orf135 | 0,35 |
| | ZFHX4 | -0,38 |
| | GPR177 | -0,34 |
| | ZNF165 | 0,34 |
| | STRA13 | 0,36 |
| | DUSP6 | -0,34 |
| | SEMA5A | -0,38 |
| | POLR2D | 0,39 |
| | THYN1 | -0,32 |
| | C11orf63 | -0,36 |
| | AQP1 | -0,32 |
| | SNRPD3 | 0,36 |
| | GAS6 | -0,35 |
| | WDR45L | 0,34 |
| | PER1 | -0,36 |
| | ESD | -0,33 |
| | LAS1L | 0,36 |
| | SLC26A3 | -0,32 |
| | CCHCR1 | 0,3 |

| | | |
|---|---|---|
| | DUSP13 | 0,3 |
| | NCALD | -0,28 |
| | CXCL2 | -0,23 |
| | IER3 | 0,29 |
| | C20orf44 | 0,29 |
| | SOX12 | 0,29 |
| | CDK5RAP3 | 0,3 |
| | DDC | -0,26 |
| | POLR2L | 0,29 |
| | CLIC3 | -0,29 |
| | ZNF226 | 0,29 |
| | GPR162 | 0,27 |
| | CNNM4 | 0,3 |
| | ID4 | -0,3 |
| | DIO1 | 0,28 |
| | C3orf60 | 0,28 |
| | CXADR | -0,29 |
| | CSTA | -0,3 |
| | F7 | 0,29 |
| | MDFI | -0,29 |
| | ING2 | 0,29 |
| | C20orf4 | 0,2 |
| | ACOX3 | 0,29 |
| | NDE1 | 0,28 |
| | PVALB | 0,23 |
| | C14orf93 | 0,25 |
| | TNFRSF11A | -0,3 |
| | RNF43 | 0,28 |
| | PTGFR | -0,22 |
| | CTNND2 | 0,27 |
| | ZNF217 | 0,28 |
| | SPOP | 0,27 |
| | DUSP5 | 0,28 |
| | MSX2 | 0,3 |
| | TPD52 | 0,26 |
| | CCDC19 | 0,29 |
| | PTPRZ1 | -0,26 |
| | SSBP2 | 0,26 |
| | SLC9A1 | 0,27 |
| | ZNF239 | 0,27 |
| | GALNS | -0,23 |
| | MKS1 | 0,25 |
| | PNPO | 0,26 |
| | NA | 0,27 |
| | NUBP1 | 0,24 |

| | | |
|---|---|---|
| | DHTKD1 | 0,3 |
| | PELI2 | -0,34 |
| | FOS | -0,35 |
| | C1RL | -0,35 |
| | TGIF2 | 0,35 |
| | HSD17B10 | 0,34 |
| | TIMM10 | 0,34 |
| | GPR124 | -0,34 |
| | SPRY1 | -0,35 |
| | RPS6KB2 | 0,32 |
| | ARHGAP11A | 0,27 |
| | SLC39A4 | 0,35 |
| | TGFBR3 | -0,33 |
| | RUVBL1 | 0,27 |
| | GALC | -0,34 |
| | RUNX1T1 | -0,35 |
| | TTF2 | 0,33 |
| | EIF4A3 | 0,33 |
| | HNRPA2B1 | 0,33 |
| | SS18L1 | 0,32 |
| | SORBS1 | -0,34 |
| | THBD | -0,31 |
| | FZD4 | -0,35 |
| | CDH5 | -0,33 |
| | F3 | -0,33 |
| | NA | -0,3 |
| | GINS3 | 0,35 |
| | TSEN34 | 0,32 |
| | TRIM24 | 0,32 |
| | CSTF2 | 0,32 |
| | DLX2 | -0,32 |
| | PLXDC1 | -0,28 |
| | LIMCH1 | -0,31 |
| | C17orf41 | 0,3 |
| | NR2F2 | -0,32 |
| | WNT5A | -0,31 |
| | C20orf19 | -0,33 |
| | ERMAP | -0,29 |
| | FCGBP | -0,31 |
| | STEAP1 | -0,3 |
| | TRPM2 | 0,29 |
| | TFPT | 0,27 |
| | NDUFS1 | 0,3 |
| | LOH11CR2A | -0,3 |
| | ICT1 | 0,29 |

| | | |
|---|---|---|
| | KCNMB1 | -0,22 |
| | SLC13A3 | -0,27 |
| | NCL | -0,26 |
| | NARFL | 0,2 |
| | LPHN2 | -0,25 |
| | N6AMT1 | 0,22 |
| | DYNC1LI2 | -0,25 |
| | NA | 0,24 |
| | ABHD9 | -0,27 |
| | SYT13 | 0,24 |
| | MATN4 | -0,21 |
| | B3GNT3 | -0,24 |
| | PLS1 | 0,25 |
| | BCOR | 0,23 |
| | NA | -0,25 |
| | MARK1 | -0,24 |
| | SLC38A1 | 0,24 |
| | WASF3 | -0,18 |
| | CD8B | -0,24 |
| | IRX4 | -0,23 |
| | EPS15L1 | 0,23 |
| | SATB1 | -0,24 |
| | WDR25 | 0,21 |
| | NA | 0,23 |
| | IARS2 | 0,23 |
| **Signature:** | **GENE21** | |
| PMID | 15591335 | |
| Comments | NA | |
| Genes/coef: | MKI67 | 1 |
| | AURKA | 1 |
| | BIRC5 | 1 |
| | CCNB1 | 1 |
| | MYBL2 | 1 |
| | MMP11 | 1 |
| | CTSL2 | 1 |
| | GRB7 | 1 |
| | ERBB2 | 1 |
| | ESR1 | -1 |
| | PGR | -1 |
| | BCL2 | -1 |
| | SCUBE2 | -1 |
| | GSTM1 | -1 |
| | CD68 | 1 |
| | BAG1 | -1 |
| **Signature:** | **GENE70** | |

| | | |
|---|---|---|
| | ARHGDIB | -0,28 |
| | EMG1 | 0,3 |
| | AZIN1 | 0,29 |
| | AHSA1 | 0,29 |
| | APLP1 | 0,27 |
| | CHCHD2 | 0,29 |
| | DKK2 | -0,29 |
| | NA | -0,28 |
| | ABCA6 | -0,27 |
| | SOX17 | -0,28 |
| | TLR3 | -0,27 |
| | SCNN1B | 0,29 |
| | ITGB4BP | 0,26 |
| | SPRY2 | -0,29 |
| | GEM | -0,28 |
| | VTCN1 | -0,26 |
| | HMBOX1 | -0,27 |
| | FADD | 0,27 |
| | RCE1 | 0,26 |
| | ZNF250 | 0,25 |
| | KERA | -0,32 |
| | THOP1 | 0,21 |
| | CACNA2D3 | -0,26 |
| | ZNF291 | -0,26 |
| | DDX56 | 0,2 |
| | TPD52L1 | 0,26 |
| | TMEM177 | 0,27 |
| | DST | -0,25 |
| | GNAZ | 0,26 |
| | GCAT | 0,25 |
| | ISOC2 | 0,26 |
| | MYO9A | -0,25 |
| | DSCR6 | 0,23 |
| | INSIG1 | 0,28 |
| | STRAP | 0,25 |
| | SF3B2 | 0,25 |
| | SUPV3L1 | 0,25 |
| | NKRF | 0,24 |
| | RPP38 | 0,27 |
| | HOXB6 | -0,25 |
| | C17orf59 | -0,24 |
| | EEF1B2 | -0,24 |
| | COIL | 0,23 |
| | BCKDHB | -0,26 |
| | S100P | 0,23 |

| | | |
|---|---|---|
| PMID | 11823860 | |
| Comments | NA | |
| Genes/coef: | TSPYL5 | -0,53 |
| | NA | -0,47 |
| | NA | -0,43 |
| | NUSAP1 | -0,42 |
| | NA | -0,42 |
| | NA | -0,41 |
| | NA | -0,41 |
| | DIAPH3 | -0,4 |
| | C16orf61 | -0,4 |
| | EXT1 | -0,4 |
| | FLT1 | -0,4 |
| | GNAZ | -0,4 |
| | OXCT1 | -0,39 |
| | MMP9 | -0,39 |
| | NA | -0,39 |
| | NA | -0,39 |
| | GMPS | -0,39 |
| | NDC80 | -0,39 |
| | CDC42BPA | -0,38 |
| | SERF1A | -0,38 |
| | AYTL2 | -0,38 |
| | NA | -0,38 |
| | RAB6A | -0,38 |
| | UCHL5 | -0,37 |
| | MTDH | -0,37 |
| | NA | -0,37 |
| | MELK | -0,37 |
| | COL4A2 | -0,37 |
| | DTL | -0,37 |
| | DCK | -0,37 |
| | NA | -0,37 |
| | GPR126 | -0,37 |
| | SLC2A3 | -0,37 |
| | ORC6L | -0,37 |
| | RFC4 | -0,37 |
| | NA | -0,37 |
| | NA | -0,36 |
| | MCM6 | -0,36 |
| | NA | -0,36 |
| | IGFBP5 | -0,36 |
| | HRASLS | -0,36 |
| | PITRM1 | -0,36 |
| | IGFBP5 | -0,36 |

| | | |
|---|---|---|
| | LETM1 | 0,23 |
| | YBX2 | 0,2 |
| | TOMM34 | 0,2 |
| **Signature:** | **GGI_grading** | |
| PMID | 16478745 | |
| Comments | NA | |
| Genes/coef: | UBE2C | 2,12 |
| | RACGAP1 | 1,9 |
| | KPNA2 | 1,73 |
| | CEP55 | 1,73 |
| | PTTG1 | 1,73 |
| | KIF4A | 1,71 |
| | TPX2 | 1,66 |
| | FOXM1 | 1,66 |
| | AURKA | 1,65 |
| | AURKA | 1,65 |
| | KIF20A | 1,65 |
| | DDX39 | 1,64 |
| | DLG7 | 1,63 |
| | MELK | 1,62 |
| | CCNA2 | 1,62 |
| | NUDT1 | 1,61 |
| | KIAA0186 | 1,61 |
| | BIRC5 | 1,6 |
| | MYBL2 | 1,6 |
| | KPNA2 | 1,59 |
| | KIFC1 | 1,58 |
| | KIF2C | 1,58 |
| | ASPM | 1,57 |
| | SPAG5 | 1,57 |
| | CENPA | 1,56 |
| | CDC20 | 1,56 |
| | ESPL1 | 1,54 |
| | MCM2 | 1,54 |
| | FEN1 | 1,52 |
| | TIMELESS | 1,52 |
| | DONSON | 1,52 |
| | CDC2 | 1,51 |
| | CDCA8 | 1,5 |
| | CCNB1 | 1,5 |
| | CDKN3 | 1,49 |
| | KIF11 | 1,49 |
| | CENPA | 1,49 |
| | DKFZp762E1312 | 1,49 |
| | MCM10 | 1,49 |

| | | |
|---|---|---|
| | NMU | -0,36 |
| | PALM2-AKAP2 | -0,36 |
| | PRC1 | -0,36 |
| | NA | -0,36 |
| | CENPA | -0,36 |
| | NA | -0,36 |
| | NA | -0,36 |
| | ESM1 | -0,36 |
| | C20orf46 | -0,36 |
| | NA | 0,359 |
| | AP2B1 | 0,363 |
| | MS4A7 | 0,364 |
| | PECI | 0,366 |
| | STK32B | 0,368 |
| | TGFB3 | 0,372 |
| | PECI | 0,373 |
| | NA | 0,374 |
| | NA | 0,375 |
| | GSTM3 | 0,381 |
| | WISP1 | 0,384 |
| | NA | 0,391 |
| | SCUBE2 | 0,4 |
| | NA | 0,402 |
| | BBC3 | 0,407 |
| | NA | 0,409 |
| | FGF18 | 0,411 |
| | ALDH4A1 | 0,421 |
| **Signature:** | **Immune_Perez** | |
| PMID | 25605861 | |
| Comments | NA | |
| Genes/coef: | AFAP1L2 | 1 |
| | AMICA1 | 1 |
| | CCL21 | 1 |
| | CCR4 | 1 |
| | CD1E | 1 |
| | CD40LG | 1 |
| | CXCL12 | 1 |
| | FYN | 1 |
| | HLA-DOB | 1 |
| | IGFBP4 | 1 |
| | IRF8 | 1 |
| | PTGDR | 1 |
| | PTGER4 | 1 |
| | TLR10 | 1 |
| **Signature:** | **IRM_immune** | |

| | | |
|---|---|---|
| | MARS | 1,49 |
| | TRIP13 | 1,47 |
| | CCNB2 | 1,47 |
| | TROAP | 1,46 |
| | FLJ20641 | 1,46 |
| | SLC7A5P1 | 1,45 |
| | LMNB1 | 1,45 |
| | CCNE2 | 1,45 |
| | CDC2 | 1,45 |
| | AURKB | 1,44 |
| | CENPE | 1,44 |
| | BUB1B | 1,44 |
| | CDC2 | 1,43 |
| | CENPI | 1,42 |
| | NCAPH | 1,42 |
| | GTSE1 | 1,41 |
| | HMMR | 1,41 |
| | MKI67 | 1,41 |
| | CCNA2 | 1,41 |
| | POLQ | 1,39 |
| | ZWINT | 1,39 |
| | TMPO | 1,39 |
| | FEN1 | 1,39 |
| | RRM2 | 1,39 |
| | GMPS | 1,38 |
| | MKI67 | 1,38 |
| | MLF1IP | 1,38 |
| | KIF2C | 1,35 |
| | PLK1 | 1,35 |
| | BLM | 1,35 |
| | LOC146909 | 1,35 |
| | BUB1 | 1,34 |
| | OIP5 | 1,34 |
| | SHMT2 | 1,34 |
| | TUBA1B | 1,33 |
| | UBE2S | 1,32 |
| | CMC2 | 1,32 |
| | H2AFZ | 1,32 |
| | MCM4 | 1,32 |
| | FLJ10156 | 1,32 |
| | TTK | 1,31 |
| | TUBA1C | 1,31 |
| | C20orf24 | 1,31 |
| | RRM2 | 1,31 |
| | MARS | 1,31 |

| | | |
|---|---|---|
| PMID | 17683518 | |
| Comments | NA | |
| Genes/coef: | XCL2 | 1 |
| | HLA-F | 1 |
| | C1QA | 1 |
| | TNFRSF17 | 1 |
| | SPP1 | -1 |
| | LY9 | 1 |
| | IGLC2 | 1 |
| **Signature:** | **STAT1_immune** | |
| PMID | 18698033 | |
| Comments | NA | |
| Genes/coef: | STAT1 | 1 |
| | CXCL10 | 0,79 |
| | TAP1 | 0,77 |
| | CXCL11 | 0,73 |
| | INDO | 0,69 |
| | CXCL9 | 0,71 |
| | MX1 | 0,7 |
| | LAMP3 | 0,69 |
| | ISG15 | 0,69 |
| | RTP4 | 0,67 |
| | HERC6 | 0,68 |
| | IFI44L | 0,68 |
| | MX2 | 0,68 |
| | IFIT3 | 0,68 |
| | HERC5 | 0,65 |
| | RSAD2 | 0,65 |
| | DDX58 | 0,64 |
| | CCL5 | 0,66 |
| | ADAMDEC1 | 0,64 |
| | CD2 | 0,64 |
| | NA | 0,61 |
| | HCP5 | 0,61 |
| | NMI | 0,6 |
| | SPOCK2 | 0,58 |
| | CCL8 | 0,57 |
| | TRIM22 | 0,59 |
| | LYZ | 0,54 |
| | IRF1 | 0,59 |
| | LAG3 | 0,54 |
| | PSCDBP | 0,57 |
| | TFEC | 0,6 |
| | UBD | 0,58 |
| | SP140 | 0,58 |

| | | |
|---|---|---|
| | PRC1 | 1,31 |
| | CENPF | 1,3 |
| | CENPN | 1,3 |
| | CDC25A | 1,3 |
| | MKI67 | 1,3 |
| | TUBA1B | 1,29 |
| | NUSAP1 | 1,29 |
| | EXO1 | 1,29 |
| | MAD2L1 | 1,28 |
| | BIRC5 | 1,28 |
| | NDC80 | 1,28 |
| | MCM4 | 1,28 |
| | CDK2 | 1,28 |
| | DCC1 | 1,28 |
| | UBE2N | 1,28 |
| | ESPL1 | 1,27 |
| | HCAP-G | 1,27 |
| | KIF14 | 1,26 |
| | CDCA3 | 1,26 |
| | SLC7A5 | 1,26 |
| | CCT5 | 1,26 |
| | ORMDL2 | 1,26 |
| | TUBA1B | 1,25 |
| | JMJD6 | 1,25 |
| | RNASEH2A | 1,24 |
| | BIRC5 | 1,24 |
| | HMGB3 | 1,24 |
| | KIF15 | 1,24 |
| | STARD13 | -1,47 |
| | IFT88 | -1,46 |
| | SESN1 | -1,42 |
| | BBS1 | -1,41 |
| | CX3CR1 | -1,38 |
| | FRY | -1,35 |
| | FLJ21062 | -1,32 |
| | CYBRD1 | -1,28 |
| | FLJ20477 | -1,28 |
| | LAMB2 | -1,27 |
| | SIRT3 | -1,27 |
| | TPT1 | -1,26 |
| | FLJ23554 | -1,26 |
| | FLJ21827 | -1,26 |
| | MPHOSPH8 | -1,25 |
| | WDR19 | -1,24 |
| **Signature:** | **STROMA_DCN.up** | |

| | | |
|---|---|---|
| | CTSC | 0,56 |
| | IFI6 | 0,56 |
| | PLA2G7 | 0,56 |
| | CD3G | 0,56 |
| | ECGF1 | 0,55 |
| | PLAC8 | 0,54 |
| | FGL2 | 0,52 |
| | GZMK | 0,53 |
| | CD48 | 0,53 |
| | STAT4 | 0,55 |
| | GPR18 | 0,52 |
| | P2RX5 | 0,5 |
| | IFI30 | 0,51 |
| | SH2D1A | 0,47 |
| | LAPTM5 | 0,5 |
| | CD69 | 0,47 |
| | PTPN7 | 0,5 |
| | IRF8 | 0,49 |
| | PIM2 | 0,48 |
| | ETV7 | 0,53 |
| | GPR171 | 0,47 |
| | PSME1 | 0,46 |
| | BIRC3 | 0,47 |
| | FASLG | 0,52 |
| | IFITM1 | 0,47 |
| | IFIT5 | 0,47 |
| | ITGB2 | 0,46 |
| | BTN3A2 | 0,46 |
| | HCLS1 | 0,45 |
| | SECTM1 | 0,43 |
| | ARHGAP15 | 0,42 |
| | KLRK1 | 0,44 |
| | IGSF6 | 0,44 |
| | EBI2 | 0,4 |
| | NA | 0,41 |
| | SNX10 | 0,4 |
| | NA | 0,39 |
| | BST2 | 0,38 |
| | NA | 0,39 |
| | APOC1 | 0,36 |
| | NA | 0,37 |
| | NA | 0,31 |
| | ZC3HAV1 | 0,34 |
| | DDAH2 | -0,33 |
| | LILRA4 | 0,34 |

| PMID | 19122658 | |
|---|---|---|
| Comments | NA | |
| Genes/coef: | DCN | 1 |
| | VCAN | 1 |
| | CDH11 | 1 |
| | COL3A1 | 1 |
| | FAP | 1 |
| | SERPINF1 | 1 |
| | FBN1 | 1 |
| | PDGFRL | 1 |
| | CTSK | 1 |
| | HTRA1 | 1 |
| | ASPN | 1 |
| | SPARC | 1 |
| | COL5A2 | 1 |
| | LOXL1 | 1 |
| | MMP2 | 1 |
| | SPON1 | 1 |
| | SFRP4 | 1 |
| | ITGBL1 | 1 |
| | CALD1 | 1 |
| | COPZ2 | 1 |
| | MFAP2 | 1 |
| | ANGPTL2 | 1 |
| | PLAU | 1 |
| | COL1A2 | 1 |
| | LRRC17 | 1 |
| | C1QTNF3 | 1 |
| | SNAI2 | 1 |
| | PCOLCE | 1 |
| | POSTN | 1 |
| | ECM2 | 1 |
| | FBLN1 | 1 |
| | ADAM12 | 1 |
| | MMP11 | 1 |
| | AEBP1 | 1 |
| | PDGFRB | 1 |
| | GAS1 | 1 |
| | COL6A3 | 1 |
| | RARRES2 | 1 |
| | COL6A1 | 1 |
| | C1R | 1 |
| | NDN | 1 |
| | TGFB3 | 1 |
| | LRP1 | 1 |

| | | |
|---|---|---|
| | EBI3 | 0,28 |
| | KLRC3 | 0,27 |
| | CLEC4A | 0,35 |
| | CD40LG | 0,33 |
| | VAV1 | 0,35 |
| | GLRX | 0,31 |
| | ACP5 | 0,28 |
| | RFX5 | 0,29 |
| | CECR1 | 0,31 |
| | TRAF3 | 0,25 |
| | RAB8A | 0,27 |
| | IL18 | 0,27 |
| | EFNA1 | -0,26 |
| | RASGRP1 | 0,26 |
| | REC8L1 | 0,26 |
| | CCRL2 | 0,32 |
| | DNAL4 | -0,22 |
| **Signature:** | **STROMA_SDPP** | |
| PMID | 18438415 | |
| Comments | NA | |
| Genes/coef: | GZMA | 1 |
| | CD8A | 1 |
| | TRBV5-4 | 1 |
| | CD52 | 1 |
| | CD247 | 1 |
| | CD48 | 1 |
| | PLEK | 1 |
| | RUNX3 | 1 |
| | GIMAP5 | 1 |
| | LCP1 | 1 |
| | F2RL2 | 1 |
| | SLC40A1 | 1 |
| | FRZB | 1 |
| | RAI2 | 1 |
| | HOXA10 | 1 |
| | ITGBL1 | 1 |
| | OGN | 1 |
| | C21orf34 | 1 |
| | ADRA2A | 1 |
| | CXCL14 | 1 |
| | SPP1 | 1 |
| | HRASLS | 1 |
| | VGLL1 | 1 |
| | ADM | 1 |
| | C6orf168 | 1 |

| | | |
|---|---|---|
| | COL10A1 | 1 |
| | DPYSL3 | 1 |
| | OLFML2B | 1 |
| | MMP14 | 1 |
| | DACT1 | 1 |
| | MXRA8 | 1 |
| | THBS2 | 1 |
| **Signature:** | **PLAU_invasion** | |
| PMID | 18698033 | |
| Comments | NA | |
| Genes/coef: | PLAU | 1 |
| | BMP1 | 0,69 |
| | MMP14 | 0,67 |
| | THY1 | 0,61 |
| | COL5A2 | 0,57 |
| | ADAM12 | 0,55 |
| | ANGPTL2 | 0,57 |
| | MFAP2 | 0,57 |
| | SERPINH1 | 0,55 |
| | COL6A1 | 0,55 |
| | ISLR | 0,51 |
| | PDLIM7 | 0,53 |
| | PARVA | 0,48 |
| | OLFML2B | 0,52 |
| | TAGLN | 0,5 |
| | CTSA | 0,48 |
| | PDGFRB | 0,48 |
| | MXRA8 | 0,44 |
| | OSMR | 0,43 |
| | COL3A1 | 0,45 |
| | GREM1 | 0,43 |
| | FAP | 0,45 |
| | DBN1 | 0,43 |
| | BICD2 | 0,43 |
| | TNFRSF12A | 0,44 |
| | VDR | 0,42 |
| | SNAI2 | 0,41 |
| | EPB41L2 | 0,42 |
| | FKBP14 | 0,43 |
| | NBL1 | 0,41 |
| | CAP1 | 0,41 |
| | ATP6V1B2 | 0,39 |
| | EPHB4 | 0,34 |
| | TRAM2 | 0,37 |
| | DDR2 | 0,38 |

| | | |
|---|---|---|
| | SNTG2 | 1 |
| **Signature:** | **VEGF_angiogenesis** | |
| PMID | 18698033 | |
| Comments | NA | |
| Genes/coef: | VEGFA | 1 |
| | CD1C | -0,3 |
| | LMO2 | -0,35 |
| | MEOX1 | -0,35 |
| | SEC61A1 | 0,35 |
| | RPL36AL | -0,34 |
| | LY86 | -0,29 |
| | CARD8 | -0,3 |
| | DNASE1L3 | -0,3 |
| | CHKA | 0,29 |
| | ATF5 | 0,22 |
| | MLYCD | -0,24 |
| | LEMD3 | -0,27 |
| | KLF13 | 0,24 |
| **Signature:** | **Continuous Hypoxia** | |
| PMID | 25216520 | |
| Comments | upregulated genes | |
| Genes/coef: | C11orf10 | 1 |
| | KDM3A | 1 |
| | RPS28 | 1 |
| | SEC61G | 1 |
| | RPS28 | 1 |
| | RPS28 | 1 |
| | ANKZF1 | 1 |
| | ALDOA | 1 |
| | P4HA1 | 1 |
| | C4orf3 | 1 |
| | HIST1H4A | 1 |
| | DDT | 1 |
| | INSIG2 | 1 |
| | RPLP2 | 1 |
| | PFKFB4 | 1 |
| | BNIP3L | 1 |
| | P4HA2 | 1 |
| | ISCA1 | 1 |
| | KPNB1 | 1 |
| | DDIT4 | 1 |
| | RBX1 | 1 |
| | HIST1H1C | 1 |
| | PYCRL | 1 |
| | NA | 1 |

| | | |
|---|---|---|
| | GFPT2 | 0,42 |
| | NID1 | 0,43 |
| | OFD1 | -0,34 |
| | IGSF4 | 0,33 |
| | STAB1 | 0,35 |
| | TPST2 | 0,29 |
| | PPP1R15A | 0,33 |
| | PDLIM3 | 0,34 |
| | ATPIF1 | -0,33 |
| | TRIM33 | -0,33 |
| | MMP3 | 0,3 |
| | EPYC | 0,34 |
| | ANKRD46 | -0,32 |
| | CPNE1 | 0,32 |
| | BCL3 | 0,3 |
| | GLB1 | 0,32 |
| | UBL5 | -0,27 |
| | ULK1 | 0,27 |
| | NOL8 | -0,27 |
| | TGFB2 | 0,29 |
| | PDGFB | 0,27 |
| | BASP1 | 0,24 |
| | SDS | 0,25 |
| | RPS27A | -0,26 |
| | ENC1 | 0,23 |
| | AGC1 | 0,21 |
| | ZNF518 | -0,28 |
| | GPR89A | -0,25 |
| | RPL18 | -0,24 |
| | MEF2A | 0,21 |
| | DNASE1L1 | 0,23 |
| | MYO1B | 0,23 |
| | JPH2 | 0,16 |
| **Signature:** | **Hypoxia** | |
| PMID | 20087356 | |
| Comments | top 15 upregulated genes | |
| Genes/coef: | VEGFA | 1 |
| | SLC2A1 | 1 |
| | PGAM1 | 1 |
| | ENO1 | 1 |
| | LDHA | 1 |
| | TPI1 | 1 |
| | P4HA1 | 1 |
| | MRPS17 | 1 |
| | CDKN3 | 1 |

| | | |
|---|---|---|
| | ANP32D | 1 |
| | ANKRD37 | 1 |
| | PNRC1 | 1 |
| | GPATCH4 | 1 |
| | PPFIA4 | 1 |
| | ANP32C | 1 |
| | NDUFAF2 | 1 |
| | RPS2 | 1 |
| | CLK3 | 1 |
| | RPS13 | 1 |
| | ANP32A | 1 |
| | VEGFA | 1 |
| | ACAP1 | 1 |
| | PHPT1 | 1 |
| | PGK1 | 1 |
| | PRMT3 | 1 |
| | MXI1 | 1 |
| | MAP1LC3B | 1 |
| | KCTD11 | 1 |
| | RRP15 | 1 |
| | PTPRCAP | 1 |
| | HIST1H4B | 1 |
| | HIST1H4C | 1 |
| | C3orf26 | 1 |
| | RNASEH1 | 1 |
| | WSB1 | 1 |
| | AIDA | 1 |
| | NA | 1 |
| | HIST1H2AB | 1 |
| | SSNA1 | 1 |
| | UTP20 | 1 |
| | TIMM23 | 1 |
| | NDRG1 | 1 |
| | PFKFB3 | 1 |
| | FAM162A | 1 |
| | MRPL19 | 1 |
| | MRPL3 | 1 |
| | RPPH1 | 1 |
| | PSME3 | 1 |
| | HCFC1R1 | 1 |
| | HIST1H2AC | 1 |
| | PSMD7 | 1 |
| | EBNA1BP2 | 1 |
| | HINT1 | 1 |
| | PGAM1 | 1 |

| | | |
|---|---|---|
| | ADM | 1 |
| | NDRG1 | 1 |
| | TUBB6 | 1 |
| | ALDOA | 1 |
| | MIF | 1 |
| | ACOT7 | 1 |

| | | |
|---|---|---|
| | PSMC3 | 1 |
| | DDIT3 | 1 |
| | FUT11 | 1 |
| | TNFSF8 | 1 |
| | CCDC41 | 1 |
| | KLHDC10 | 1 |
| | CCNG2 | 1 |
| | RPL39 | 1 |
| | RPL39 | 1 |
| | HTR5A | 1 |
| | PGAM1 | 1 |
| | PNPT1 | 1 |
| | UBTF | 1 |
| | KRTAP10-12 | 1 |
| | PPP2R5B | 1 |
| | SNORA68 | 1 |
| | GPI | 1 |
| | TAGAP | 1 |
| | TMEM88 | 1 |
| | AK4 | 1 |
| | MAD1L1 | 1 |
| | HIST1H3B | 1 |
| | C3orf58 | 1 |
| | CHD1 | 1 |
| | NDUFA1 | 1 |
| | PDK1 | 1 |
| | WBP1 | 1 |
| | WBP1 | 1 |
| | POU5F1P3 | 1 |
| | MAP1LC3B2 | 1 |
| | ATF4 | 1 |
